# Analysis of growth factor signaling in genetically diverse breast cancer lines

**DOI:** 10.1186/1741-7007-12-20

**Published:** 2014-03-21

**Authors:** Mario Niepel, Marc Hafner, Emily A Pace, Mirra Chung, Diana H Chai, Lili Zhou, Jeremy L Muhlich, Birgit Schoeberl, Peter K Sorger

**Affiliations:** 1 HMS LINCS Center, Department of Systems Biology, Harvard Medical School, 200 Longwood Ave, WAB 444, Boston, MA 02115, USA; 2 Merrimack Pharmaceuticals, 1 Kendall Sq, Cambridge, MA 02139, USA

**Keywords:** Breast cancer, Microenvironment, Receptor tyrosine kinases, Signal transduction, Growth factors

## Abstract

**Background:**

Soluble growth factors present in the microenvironment play a major role in tumor development, invasion, metastasis, and responsiveness to targeted therapies. While the biochemistry of growth factor-dependent signal transduction has been studied extensively in individual cell types, relatively little systematic data are available across genetically diverse cell lines.

**Results:**

We describe a quantitative and comparative dataset focused on immediate-early signaling that regulates the AKT (AKT1/2/3) and ERK (MAPK1/3) pathways in a canonical panel of well-characterized breast cancer lines. We also provide interactive web-based tools to facilitate follow-on analysis of the data. Our findings show that breast cancers are diverse with respect to ligand sensitivity and signaling biochemistry. Surprisingly, triple negative breast cancers (TNBCs; which express low levels of ErbB2, progesterone and estrogen receptors) are the most broadly responsive to growth factors and HER2^amp^ cancers (which overexpress ErbB2) the least. The ratio of ERK to AKT activation varies with ligand and subtype, with a systematic bias in favor of ERK in hormone receptor positive (HR^+^) cells. The factors that correlate with growth factor responsiveness depend on whether fold-change or absolute activity is considered the key biological variable, and they differ between ERK and AKT pathways.

**Conclusions:**

Responses to growth factors are highly diverse across breast cancer cell lines, even within the same subtype. A simple four-part heuristic suggests that diversity arises from variation in receptor abundance, an ERK/AKT bias that depends on ligand identity, a set of factors common to all receptors that varies in abundance or activity with cell line, and an “indirect negative regulation” by ErbB2. This analysis sets the stage for the development of a mechanistic and predictive model of growth factor signaling in diverse cancer lines. Interactive tools for looking up these results and downloading raw data are available at http://lincs.hms.harvard.edu/niepel-bmcbiol-2014/.

## Background

Receptor tyrosine kinase (RTK) signaling is an important form of cell-cell communication involving 58 human receptors falling into 20 families [1]. Each RTK binds to one or more soluble or membrane-bound ligands (growth factors) that promote the formation of receptor homo- and hetero-oligomers and assembly of multi-component signaling complexes (except that the ErbB2 RTK has no known ligand and normally functions as a hetero-oligomer). Receptor-bound signaling proteins activate ‘immediate-early’ signaling by MAPK, PI3K/AKT, and other kinase cascades and regulate motility, differentiation, adhesion, proliferation and cell survival. The biochemistry of RTK signaling proteins has been characterized extensively, but relatively little systematic data are available on the diversity of signaling responses mediated by these proteins across cell lines.

Many RTKs are mutated, over-expressed, or dysregulated in cancer and a large number of anti-cancer drugs targeting RTKs are in use or in development [2]. In some cases these drugs bind primary oncogenic drivers such as ErbB2, which is overexpressed in the HER2^amp^ breast cancer subtype [3], or ErbB1, which is mutated in non-small cell lung cancer [4]. In other cases, RTKs promote oncogenesis or alter drug sensitivity by responding to paracrine and autocrine ligands present in the microenvironment, produced either by the tumor itself or by the surrounding stroma. For example, the presence of ErbB ligands in colorectal cancer is correlated with increased survival following treatment with cetuximab, a therapeutic antibody targeting EGFR [5,6]. Conversely, the presence of ErbB ligands promotes resistance to ErbB therapy in other cancers [7-9] and HGF production by stromal cells is a factor in the preexisting resistance of BRAF-V600E melanomas to vemurafinib [10]. The latter observation is one motivation for clinical development of drugs inhibiting the HGF receptor cMet [10].

To date, systems-level studies of immediate-early signal transduction have focused primarily on increasing the number of proteins analyzed. For example, mass spectrometry has revealed the kinetics of phosphorylation of approximately 1,000 substrates in EGF-treated HeLa cells [11], and reverse phase lysate arrays have provided data on approximately 50 substrates in five cell lines exposed to seven growth factors [12], and on six isogenic lines ectopically over-expressing individual RTKs [13]. Analysis of receptor-mediated signal transduction in a particular tumor cell line is subject to the criticism that no line is representative of human cancer. However, generalizing across cell lines is complicated by the occurrence of large, but poorly characterized, variability. Recent genomic and expression profiling experiments have demonstrated the value of systematically analyzing such variability and it appears that a significant fraction of the complexity of specific human cancers can be captured using banks of genetically diverse cell lines [14,15].

In this paper we analyzed growth factor responsiveness in a canonical collection of 39 breast cancer cell lines of the NCI-ICBP43 set, whose genotypes span many of the mutations observed in primary disease [16]. The data comprised the phosphorylation levels of ERK (MAPK1/3) and AKT (AKT1/2/3) kinases following exposure to 15 different growth factors at two doses and three time points, as well as the abundance and phosphorylation levels of over 20 RTKs [17]. The goals of the analysis were to: (i) characterize the diversity of response and determine how it mapped to clinical subtypes, (ii) identify factors that controlled the magnitude and duration of ligand response, and (iii) generate a simple means to look up and compare ligand-response data that have hitherto been unavailable or scattered across the literature. We found that breast cancer cell lines exhibit highly diverse responses to growth factors with lines belonging to the “triple negative” (TNBC) subtype being the most broadly responsive and HER2^amp^ lines the least. The magnitude of ligand responses appeared to be determined by four primary factors: the identity of the ligand, the abundance of the cognate RTK, regulators common to all receptors but different for AKT and ERK and differing in levels or activity with cell type, and indirect negative regulation mediated by the ErbB2 receptor. The significance of these factors varies depending on whether fold-change or absolute response is regarded as the biologically significant feature.

## Results

We generated a multidimensional growth factor response dataset with axes corresponding to cell line, ligand identity, ligand dose, exposure time, and downstream target (see Additional file 1: Table S1). To facilitate visualization of the data we constructed a set of node-edge graphs (one per cell line) in which the diameter and shading of a node is proportional to the logarithm of the steady state protein abundance and basal phosphorylation level, respectively. The weight of an arrow connecting ligands to pAKT (red) or pERK (blue) denotes the magnitude of the response based on the maximal value across time points at the highest dose. Under the starvation conditions used for these assays, basal phosphorylation levels are likely to reflect autocrine signaling, the presence of activating mutations in signaling proteins, or, in the case of cells expressing high levels of ErbB2, receptor auto-activation [18]. Edges linking ligands to receptors are a representation of published binding data (see Additional file 2: Table S2) and differences in affinity are not depicted.

**Figure 1:**
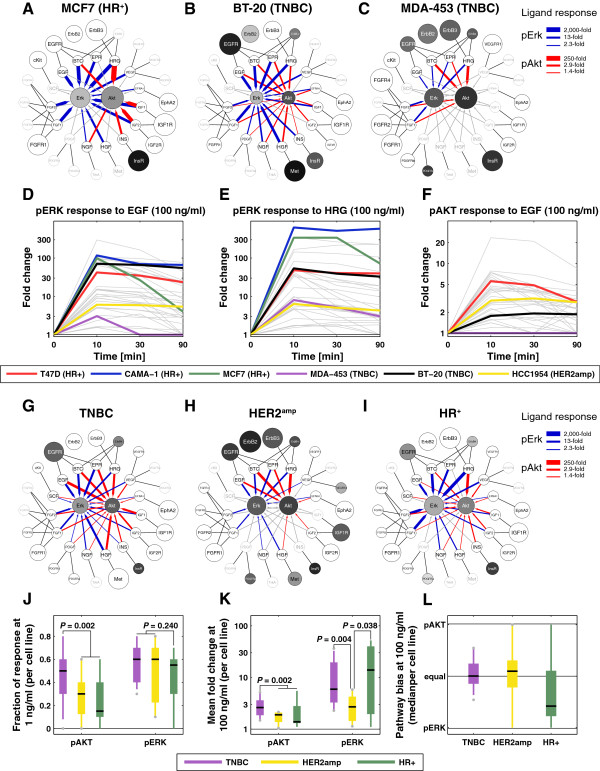
**Landscape of signaling responses across breast cancer cell lines. (A-C)** Node-edge graphs of **(A)** MCF7 **(A)**, BT-20 **(B)**, and MDA-MB-453 **(C)** depicting induction of pERK (blue arrows) and pAKT (red arrows) by different ligands. The arrows’ thickness denotes the maximum induction across all time points and doses, normalized for each target across all cell lines and ligands. Gray arrows denote non-significant responses (less than two standard deviations above the control). Outer and inner circles represent basal measurements where the diameter is proportional to the logarithm of the expression level and shading to the logarithm of the phosphorylation level; data were normalized across all cell lines. pFGFR1-4 were not measured. Gray outer circles and labels denote expression levels below the detection threshold and their diameter denotes the minimal circle size. Lines show which ligands bind which receptors (Additional file 2: Table S2). **(D-F)** Fold-change in pERK **(D-E)** or pAKT **(F)** levels following exposure of cells to EGF **(D, F)** or HRG **(E)**. Trajectories for six specific cell lines are highlighted in color; all other trajectories are represented in gray. **(G-I)** Average node-edge graphs for each breast cancer subtypes: TNBC **(G)**, HER2^amp^**(H)**, and HR ^+^ **(I)**. Edges and nodes have the same meaning as in **(A)** except that circle size, intensity of shading, and edge width are proportional to the interquartile mean across lines of the subtype. **(J)** Fraction of cells of each subtype showing significant pERK or pAKT responses following exposure to ErbB, FGF, IGF/INS, or HGF ligands at 1 ng/ml. Wilcoxon’s rank-sum *P*-values are shown. **(K)** Distribution of the cell line mean fold-change following stimulation at 100 ng/ml of the same ligands as in **(J)**. **(L)** Distribution of cell line median pathway bias of the same ligands as in **(J)**.

Comparing MCF7, BT-20, and MDA-MB-453 cells (Figure 1A-C; node-edge graphs for all lines are found in Additional file 3 and at a dedicated website [19]), we see that pERK was induced by many more ligands than pAKT in MCF7 cells, pAKT and pERK were induced to a similar extent by multiple ligands in BT-20 cells, and only FGF-1/2 and ErbB ligands (EGF, BTC, HRG and BTC) elicited a significant yet muted response in MDA-MB-453 cells. Thus, breast cancer cell lines are broadly responsive to growth factors, but the extent of pERK and pAKT induction varied dramatically with the line. The connection between RTK level and ligand responsiveness was not immediately obvious (compare the size of the RTK nodes to the thickness of blue and red edges): for example, MCF7 and MDA-MB-453 expressed similar levels of insulin receptor, but only MCF7 cells had a significant response to insulin at the level of pAKT induction.

Overall, approximately 60% of cell-ligand combinations resulted in significant induction of pERK (where significant means a signal two standard deviation above the mean of untreated control cells) and approximately 50% of combinations resulted in pAKT induction. Ligands that bind to ErbB receptors were the most broadly active (every tested ErbB ligand elicited a significant response in at least 35 lines), followed by FGF-1/2, HGF, and IGF-1/2, consistent with the known importance of these ligands in breast cancer biology. pERK induction ranged from statistically insignificant to more than 600-fold in CAMA-1 cells exposed to HRG. Moreover, induction of pERK was transient in some cell lines (for example, MCF-7, MDA-MB-453; Figure 1D) and sustained in others (for example, CAMA-1, BT20). Even within a single ligand family, substantial differences in relative responsiveness were observed: HRG and EGF induced pERK to the same degree in BT-20 and T47D cells, but HRG was much more potent than EGF in CAMA-1 and MCF-7 cells (Figure 1D, E). Differences were also observed with respect to which downstream kinases were activated: BT-20 and T47D cells were highly responsive to EGF at the level of pERK and pAKT, whereas EGF induced only pERK in CAMA-1 and MCF-7 cells (Figure 1D, F). This did not arise because the AKT pathway is defective in CAMA-1 and MCF-7 cells since HRG, another ErbB ligand, induced pAKT in both cell lines.

### Relating signaling features to cancer subtype

In the clinic, breast cancers are classified into HER2^amp^, hormone receptor positive (HR^+^), or TNBC subtypes based on the expression levels of the ErbB2 tyrosine kinase and the estrogen and progesterone nuclear hormone receptors (ER/PR). We found that TNBC cell lines as a group (Figure 1G) exhibited relatively low expression of receptors other than EGFR and had low basal pAKT or pERK activity compared to the other subtypes (Figure 1H, I). However, in these cells, pAKT and pERK were more responsive to a wider range of growth factors than in any other subtype (Figure 1J). This included responsiveness to HRG, despite the fact that HRG functions via ErbB2-containing receptor heterodimers and ErbB2 is notionally absent in TNBCs (in fact, ErbB2 is present at 10^4^ to 10^5^ molecules per cell, a high level for an RTK, albeit lower than the 10^6^ to 10^7^ molecules per cell observed in HER2^amp^ cells). As expected, HER2^amp^ cell lines exhibited the highest expression of ErbB2 and this correlated with high basal pErbB1-4, pERK and pAKT (Figure 1H, Additional file 4: Figure S1). In cells of this subtype, only ErbB ligands induced a significant response, and this was generally modest, relative to TNBC cells, as measured by fold-change in activity (Figure 1K). Finally, HR^+^ cell lines were characterized by higher levels of ErbB3, FGFR4, and IGF1R than cell lines from other subtypes (Wilcoxon’s *P* = 0.0015, *P* = 0.014 and *P* = 0.021, respectively), strong responsiveness to FGF1/2 and HRG (Figure 1I), and by a bias of ligand responses toward pERK (Figure 1L).

### Time and dose-dependence of response

**Figure 2:**
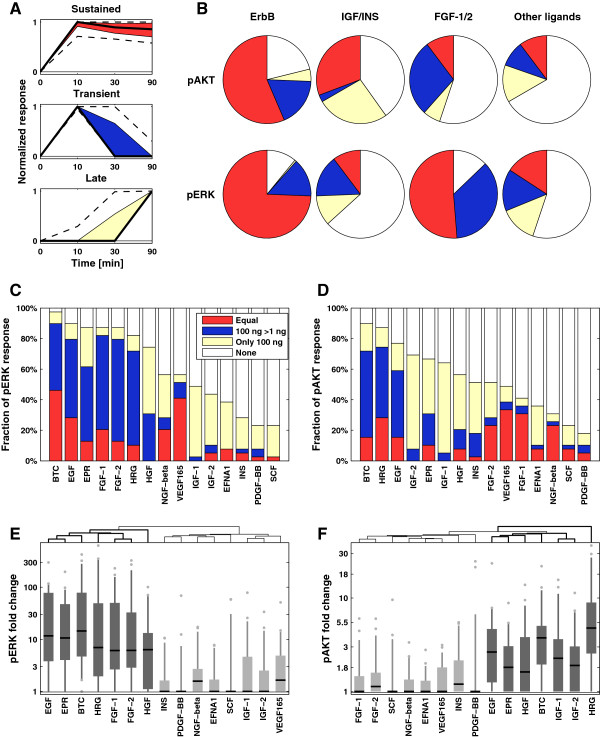
**Ligands elicit different response kinetics and exhibit distinct dose-response behavior. (A)** Clustered response trajectories (red: sustained; blue: transient; off-white: late). Black lines represent the median response and colored zones the interquartile range; dashed lines depict 10% and 90% quantiles. **(B)** Distribution of all responses by ligand family and downstream target for ErbB ligands, IGF/INS ligands, FGF-1/2 and all remaining ligands (data for 100 ng/ml are shown). **(C-D)** Distribution of sensitivity classes by ligands for pERK **(C)** and pAKT **(D)**: ‘Equal’ means that the fold-change response at low dose is at least 75% of the high dose response; ‘100 ng >1 ng’ means that low dose response is statistically significant but less than 75% of the high dose response; off-white means only the high dose response is significant. **(E-F)** Distribution of fold-change response by ligand, clustered by strength of the pERK **(E)** or pAKT **(F)** response at high ligand levels. Black lines represent the median, shaded zones the interquartile range, dashed lines the 5% and 95% quantiles, and dots the outliers. Darker gray is used for the clusters with the strongest responses.

We used clustering to determine which characteristics of immediate-early signaling are most variable across our dataset, and whether they correlate with cell line, ligand identity, or receptor family. Unsupervised k-means clustering (with k = 4) of the time-series data identified trajectories that correspond to sustained, transient, late, or no response (Figure 2A; Additional file 5). These clusters are similar to trajectories previously identified by analysis of phospho-mass spectrometry, ELISA, and reverse phase lysate array data obtained from one or a few cell lines [11-13,20]. We found that approximately 50% of all significant ligand responses were sustained and the remainders were split equally between transient and late responses (Additional file 6: Figure S2A). The responses in the sustained cluster were significantly stronger than those in other clusters (Additional file 6: Figure S2B, C). Neither dose nor downstream target (that is, pERK or pAKT) were major determinants of temporal class, but the identity of the ligand was: induction of pERK and pAKT by ErbB ligands was usually sustained (Figure 2B, first column) whereas induction of pAKT by IGF/INS ligands was either sustained or late (Figure 2B, second column). In contrast, FGF-1/2 ligands were most likely to induce transient responses (Figure 2B, third column). Fewer cell lines were responsive to the other ligands tested, and significant responses were split roughly equally among sustained, transient, and late (Figure 2B, fourth column).

**Figure 3:**
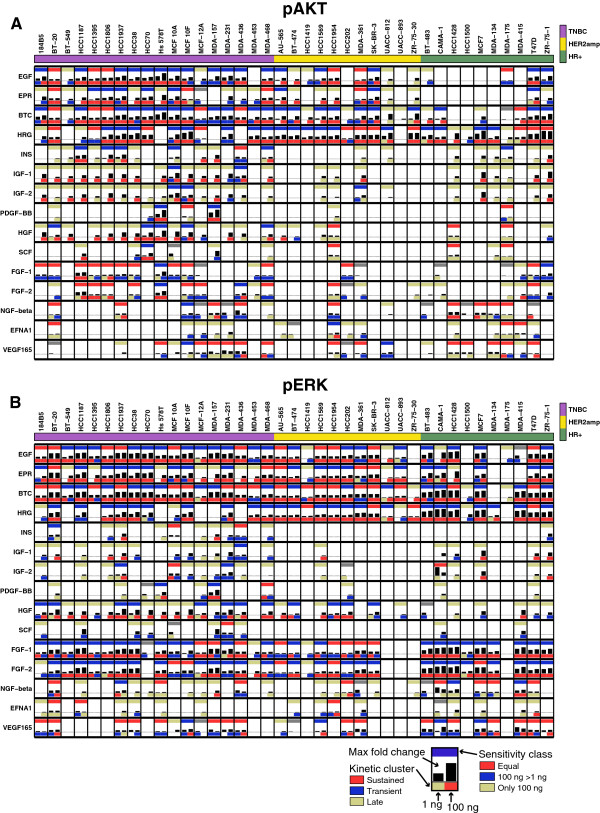
**Summary of ligand responses across all cell lines.** Data for pAKT **(A)** and pERK **(B)** in which each subplot shows the results by ligand and cell line. The upper bar is colored according to the sensitivity class (as in Figure 2C, D), the black bars show the maximal fold-change response for 1 ng/ml (left) and 100 ng/ml (right), and the color of the lower bar corresponds to the kinetic cluster for 1 ng/ml (left) and 100 ng/ml responses (right; color coded as in Figure 2A, B). Cell lines are grouped by subtype and ligands by family.

Comparing response data between the two doses tested, we found that pERK was maximally responsive to 1 ng/ml EGF in some cell lines (for example, BT20, MCF7, T47D and HCC-1954; Additional file 6: Figure S2D), but in other lines responses increased between 1 ng/ml and 100 ng/ml EGF (MDA-MB-453), and in yet other cases (CAMA-1) responses were observed only at the higher ligand concentration. Classifying response into four groups based on dose (Additional file 6: Figure S2E-F; Additional file 5) revealed that ErbB and FGF ligands were split between the groups ‘equal’ response and ‘increased at the highest dose’ , whereas HGF, IGFs, and insulin were generally active at ‘100 ng/ml only’ (Figure 2C, D). Sensitivity to low ligand doses did not necessarily coincide with strong activation at high dose, but it was strongly correlated with ligand identity. This implies that ligand identity, rather than cell type or downstream pathway, is the main determinant of low-dose responsiveness (Additional file 6: Figure S2G). This contrasts with data on the magnitude of the response, in which there is substantial variability from line to line (Figure 2E-F). For each downstream target we identified ligands that generally show strong (dark gray) or weak (light gray) responses. ErbB ligands and HGF elicit strong responses for both the ERK and AKT pathways. In contrast, IGF-1 elicited a strong response only at the level of pAKT and FGF-1 at the level of pERK. We summarize this data in reference tables that encode the magnitude of response, the kinetic clusters and the sensitivity classes for pAKT (Figure 3A) and pERK (Figure 3B).

### Factors controlling the relative magnitude of pERK and pAKT responses

**Figure 4:**
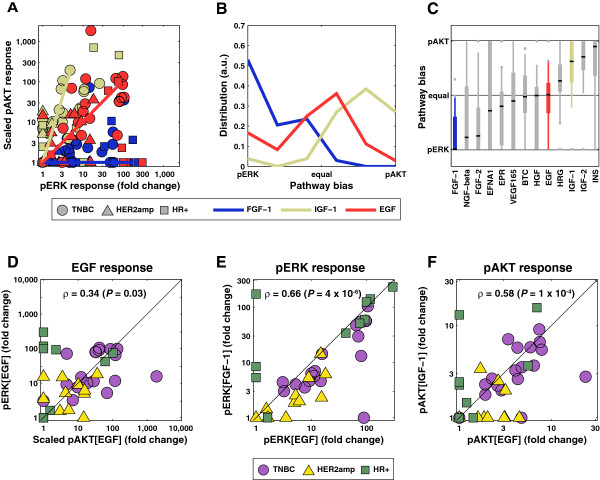
**Bias in response towards pERK or pAKT varies with ligand and cell line. (A)** Scaled pAKT (vertical axis) and pERK (horizontal axis) responses in cells exposed to 100 ng/ml IGF-1 (off-white), EGF (red), or FGF-1 (blue) with shape denoting subtype. **(B)** Distribution of the “pathway bias” for the three ligands in **(A)** where the bias is defined as the arctangent of the scaled pAKT to pERK fold-change ratio. **(C)** Distribution of the pathway bias for all ligands that exhibited at least 50% significant responses across all cell lines (at 100 ng/ml). Ligands are sorted according to the median of their pathway bias. **(D-F)** Scatter plot of pAKT and pERK responses to EGF **(D)**, pERK responses to EGF and FGF-1 **(E)**, and pAKT responses to EGF and IGF-1 **(F)**. Spearman’s correlation and *P*-value are reported.

To characterize the selectivity of receptors for downstream pathways, we computed a “pathway bias” based on the maximum pAKT and pERK responses for each ligand (note that responses for the two kinases were scaled so they could be compared directly; in the absence of scaling, pERK exhibited higher fold-changes). We found that whereas EGF elicited symmetric pERK or pAKT induction, the FGF-1 response was heavily biased towards pERK, and the response of IGF-1 was biased to pAKT (solid lines in Figure 4A and distributions in Figure 4B). On average, ligands from the same family exhibited a similar pERK-pAKT bias (with HRG being an exception among ErbB ligands; Figure 4C), but these averages obscure the fact that pERK and pAKT responses were only weakly correlated across cell lines (Spearman’s ρ = 0.30, *P* = 0.03, Figure 4D), making the pathway bias for a given ligand remarkably variable. In contrast, fold-change pERK activation by different ligands was highly correlated within a cell line (Spearman’s ρ = 0.66, *P* = 4.0 · 10^−6^, Figure 4E) and the same was true of pAKT (Spearman’s ρ = 0.58, *P* = 1.2 · 10^−4^, Figure 4F). Thus, one or more factors common to all RTKs within a given cell line appear to play a significant role in pERK inducibility and a different set of factors control pAKT.

**Figure 5:**
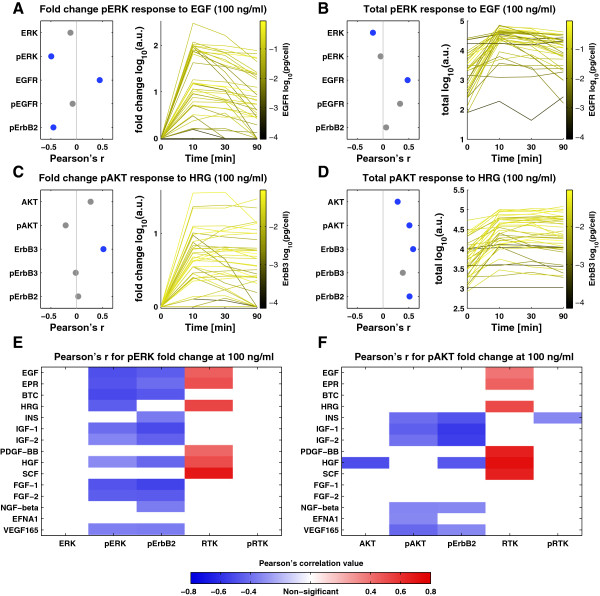
**Expression levels of target receptors correlate with response. (A)** The left panel depicts Pearson’s correlation of pERK fold-change in response to 100 ng/ml EGF and basal measures of the expression and phosphorylation levels of the downstream target, cognate RTK and pErbB2. Blue dots indicate correlations with *P* <0.05. The right panel shows the time course of pERK levels in response to EGF for individual cell lines with color denoting EGFR expression level (darker represents lower levels). **(B-D)** Plots as in **(A)** for absolute pERK levels in response to 100 ng/ml EGF **(B)**; fold-change pAKT **(C)** and absolute pAKT levels **(D)** in response 100 ng/ml HRG. **(E-F)** Pearson’s correlation between pERK **(E)** and pAKT **(F)** fold-change response and the basal expression and phosphorylation levels of the downstream kinases, the level of pErbB2, and the basal and phosphorylation levels of the cognate receptor for all tested growth factors. Red denotes significant positive correlation and blue denotes significant negative correlation (*P* <0.05).

Ligand responses are commonly reported as the ratio of the peak to basal levels and several studies have concluded that such “fold-change” response is the determining factor in cellular phenotype [21,22]. In other cases, however, the absolute signal strength following stimulation determines whether a threshold can be overcome [23]. In the case of pERK fold-change following EGF stimulation, we observed no correlation with ERK protein levels, but EGFR levels were well-correlated (Figure 5A; Pearson’s r = 0.45, *P* = 0.004). Unexpectedly, basal pErbB2 and pERK levels were negatively correlated with pERK fold-change (Pearson’s r = -0.45, *P* = 0.004). This suggests that, for fold-change induction of pERK by EGF, EGFR is a positive regulator, pErbB2 a negative regulator and ERK level does not matter. The absolute level of induced pERK behaves differently, exhibiting little correlation with pERK or pErbB2 levels, but strong positive correlation with total EGFR level (Figure 5B). These trends are observed across ligands, such as EGF, HRG, HGF, PDGF, and SCF, for which there is a single primary receptor (Figure 5E). The correlation is not significant in the case of ligands such as FGF-1/2, and we speculate that this reflects the binding of FGF ligands to multiple FGF receptors.

In the case of pAKT fold-change in cells exposed to the ErbB2/3 ligand HRG, no correlation was observed with AKT, pAKT, or pErbB2 levels, but ErbB3 levels were positively correlated (Figure 5C). Absolute levels of induced pAKT levels were positively correlated with ErbB3 levels, and negatively with pErbB2 and basal pAKT levels (Figure 5D). Basal pErbB2, therefore, appears to play a different role in regulation of ERK and AKT. In retrospect, this difference can be discerned by examining time course data (illustrated in Figure 5A, C for EGF and HRG). Basal pAKT levels were highly variable across cell lines and correlated with pAKT levels post-induction: the higher the baseline the higher the absolute induced response and vice versa. This pattern was not observed for pERK: basal levels of pERK are generally low and fold-change is high. Similar differences between pAKT and pERK were observed for other ligands that predominantly bind to a single RTK (Figure 5E, F). Thus, indirect negative regulation by ErbB2 is not as important for pAKT as for pERK and receptor level is the major factor determining pAKT fold-change.

**Figure 6:**
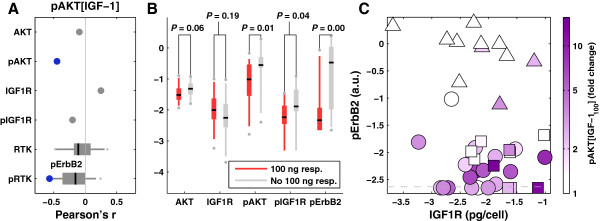
**pAKT response of IGF-1 is negatively correlated with pErbB2 basal level. (A)** Correlation of pAKT fold-change response in cells exposed to 100 ng/ml IGF-1 plotted as in 5A. **(B)** Distribution of relevant RTK expression or phosphorylation levels between lines exhibiting significant (red) or not significant (gray) pAKT response to IGF-1. Black lines represent the median, shaded zones the interquartile range, dashed lines the 5% and 95% quantiles, and dots the outliers. *P*-values were assessed by a Wilcoxon rank-sum test. **(C)** Projection of IGF1R and pErbB2 levels in each cell line overlaid with pAKT fold-change response in cells exposed to IGF-1.

The IGF/INS ligands are a striking exception to this rule. Fold-change of pAKT following exposure of cells to IGF-1 did not correlate with levels of IGF1R, but it was strongly negatively correlated with pErbB2 (Figure 6A). Even when cell lines were classified into IGF-1 responsive and non-responsive classes (independently of the magnitude of the response), no enrichment was observed with IGF1R levels (Figure 6B). This stands in contrast to most other ligands (Additional file 7: Figure S3) for which cells with high receptor levels are significantly more likely to respond. A possible explanation is the unusually strong negative correlation with basal pErbB2 levels, as illustrated in Figure 6C by a plot of pAKT induction by IGF-1 relative to IGF1R and basal pErbB2 levels (Figure 6C). Note that pAKT could be strongly induced in these cells by one or more other ligands, showing that there was no intrinsic defect in AKT signaling. We speculate that the connection between IGF responsiveness and IGF1R levels may be obscured by the interaction of IGF1R with ErbB2 [24,25] or by the presence of IGF binding proteins that are known to modify IGF responses (reviewed in [26]) and that have been linked to resistance to anti-ErbB therapy [24,27]. Strong cross-talk between IGF1R and ErbB2 may explain why over-expression of IGF1R induces resistance to the anti-ErbB2 antibody trastuzumab (Herceptin) [28].

## Discussion

In this paper we describe a systematic analysis of RTK inducibility in a collection of breast cancer cell lines previously shown to span a significant fraction of the genetic variation observed in human disease. Each examined cell line expresses multiple RTKs at levels that vary from the limit of detection (ca. 100 molecules per cell) to over 10^7^ molecules per cell. In general, the levels of RTKs are not highly correlated with each other and even HER2^amp^ cell lines, which are defined by over-expression of ErbB2, are divergent with respect to the expression and basal phosphorylation levels of other RTKs [17]. Thus, each cell line in our collection has a unique RTK fingerprint. These profiles as well as characteristics of the ligand responses are accessible on an interactive website along with the underlying data [19].

Sensitivity to ErbB1-4 ligands is a dominant feature of the breast cancer cell lines we examined: all 39 lines exhibited a statistically significant response to at least one ErbB ligand (EGF, EPR, BTC, or HRG) and 31 of the 39 lines responded to all four ligands. ErbB receptors are ubiquitously expressed in epithelial cells and breast cancers originate primarily from epithelial ductal or lobular linings [29]. However, it is striking that HER2^amp^ lines, which express the highest levels of ErbB2, are quite insensitive to exogenous ligand: high ErbB2 levels correlate negatively with fold-responsiveness of pERK to growth factors such as the ErbB2/3 ligand HRG. In addition, pAKT responsiveness to IGF ligands and insulin is strongly negatively correlated with ErbB2 levels. In marked contrast, TNBC cells, which are notionally “negative” for ErbB2, are highly responsive not only to HRG but to ErbB ligands in general.

Other ligands to which breast cancer cells commonly respond include FGFs, IGF/INS, and HGF. Responsiveness to SCF and PDGF is observed more sporadically, but can be as strong as responses to ErbB ligands when the cognate receptor is present. In general, responsiveness within the same family of ligands is highly correlated, likely because such ligands can bind overlapping sets of receptors. TNBC cells exhibit the greatest fold-change responsiveness to ErbB ligands, FGFs, IGF/INS, and HGF, and they also account for the preponderance of responsiveness to SCF and PDGF. It is tempting to speculate that one of the reasons TNBC tumors respond poorly to targeted therapies [30] is that they are sensitive to diverse ligands present in the microenvironment, many of which have been implicated in drug resistance [7-10]. In contrast, HER2^amp^ cancers are relatively insensitive to their microenvironment and are among the most treatable breast cancers. Few antibody therapeutics other than those against ErbB receptors have proven effective in breast cancer and this might be a matter not only of the importance of ErbB2 in HER2^amp^ cells, but also the down-regulation of other signaling pathways in these cells. It nonetheless seems plausible that identifying the subset of HR^+^ and TNBC cells responsive to IGFs, FGFs, and HGF might serve as means to identify those sensitive to therapeutic intervention with anti-receptor antibodies other than trastuzumab. For example, a subset of TNBC tumors might be sensitive to the inhibition of the c-Kit and PDGFR receptors.

### Determinants of ligand responsiveness

**Figure 7:**
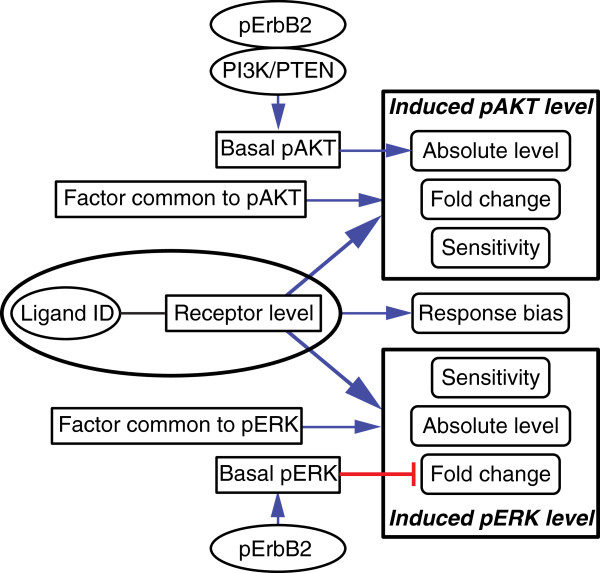
**Flow chart of dependencies between basal levels and induced levels.** This is a heuristic summarizing the primary determinants of cellular response to ligands at the level of pAKT and pERK. See text for details.

The available data is as of yet insufficient for construction of a mechanistic model of the responsiveness of breast cancer cell lines to growth factors. We therefore propose a four-factor heuristic (Figure 7) which serves as a compact summary of our findings and can be used to predict the probable behavior of cell lines not in our collection. For example, from applying this heuristic we would expect an HR^+^ cell line with a high level of cMet to have a dose-sensitive response to HGF that is strongly skewed toward pERK.

The first factor in the heuristic is the abundance of the cognate receptor: fold-change, absolute response levels, and likelihood of response all correlate positively with receptor abundance. The exceptions to this rule are IGF/INS ligands, for which receptor levels and ligand responsiveness do not correlate. The second factor is ligand and receptor identity, which appears to specify whether pERK or pAKT is most strongly induced: ErbB ligands and HGF generally induce both pERK and pAKT, whereas FGF1/2 are ERK specific and IGF/INS are AKT specific. However, ligand-induced pERK/pAKT ratios vary significantly from one cell line to the next. This appears to involve a third set of factors, which comprises response determinants that are common to all RTKs in a particular cell line, but act differently on ERK and AKT pathways. These factors are largely unknown, but might include adapter proteins, phosphatases and so on, but it appears that these factors vary in abundance with subtype (for example, HR^+^ lines are biased toward high pERK response). The fourth and most unexpected factor is an “indirect negative regulation” by ErbB2: expression levels of pErbB2 negatively correlate with pERK fold-change for all ligands examined and are a dominant factor for IGF/INS response at the level of pAKT. Ligand-induced absolute pERK levels are not correlated with pErbB2, however, so the impact of the indirect negative regulation by pErbB2 signaling depends on whether fold-change or absolute level is the critical biological feature. Moreover, for all but IGF/INS, the phenomenon is reversed for pAKT: pErbB2 and basal pAKT levels (the latter of which can also be increased by PI3K/PTEN mutations, Additional file 8: Figure S4) are positively correlated with absolute pAKT levels following ligand treatment, but not with fold-change. Several factors are noteworthy by their absence as response determinants: these include absolute levels of ERK or AKT (presumably they are present in excess), and basal RTK phosphorylation (except in the aforementioned case of pErbB2), implying that even high autocrine activation does not block responsiveness to additional, exogenous ligand.

## Conclusions

Looking forward, mechanistic analyses are needed to understand how the wide diversity of network activities observed in a panel of breast cancer cell lines arises from the action of common sets of signaling proteins. Ascertaining the biochemical basis of indirect negative regulation by ErbB2 is also likely to be worthwhile, since our data imply that the high responsiveness of HER2^amp^ tumors to anti-receptor therapy may be a function not only of the addiction to the ErbB2 oncogene, but also of the suppression of other signaling pathways that might function as resistance mechanisms in other tumor types. This would constitute a new concept in therapeutic design. Finally, further analysis of RTK expression, basal activity, and network inducibility, particularly in TNBC, may reveal possibilities for biomarker guided use of targeted drugs in blocking oncogenic pathways or resistance mechanisms in subsets of tumors.

## Methods

NCI-ICBP43 breast cancer cell lines were obtained directly from the ATCC (product 90-4300 K), confirmed to be *Mycoplasma*-free, and only early passage cultures were used. Only 39 cell lines were used for this work since four cell lines (DU-4475, HCC-2218, HCC-2157 and HCC-1599) were non-adherent and, therefore, not suited to our measurement methodology. Cells were plated to achieve approximately 75% confluence following growth in complete media for 24 h and then starved in serum-free media for 18 h (Additional file 1: Table S1). Cells were either lysed immediately or exposed to growth factors (diluted and stored according to the manufacturer’s recommendation; Additional file 1: Table S1) at a final concentration of 1 ng/ml or 100 ng/ml for 10, 30, and 90 minutes then lysed. Basal expression and phosphorylation levels of RTKs were measured using ELISA assays (Additional file 1: Table S1 for details). The levels of induced phospho-Erk1/2 (Thr202/Tyr204; pERK) and phospho-AKT (Ser473; pAKT) served as proxies for the activation of the MAPK and PI3K/AKT pathways and were also measured using ELISA assays.

Basal expression and phosphorylation levels were normalized using recombinant standards and all measurements below the level of detection were set to the value of the detection threshold. The ligand responses were measured in duplicate and averaged in the log_10_ domain. Responses with values less than two standard deviations above the mean of the controls were considered to be non-significant (corresponding to approximately 1.2-fold change). For the calculation of pathway bias, the pAKT response was linearly scaled by a factor of 2.386 to minimize the Cramér-Von Mises distance between the distribution of pERK and pAKT fold-change responses. A detailed description of data collection and processing can be found in Niepel *et al*. [17] and all data can be obtained at a dedicated website [19].

## Competing interests

PKS is a founder of Merrimack Pharmaceuticals and chair of its scientific advisory board; this relationship is actively managed by Harvard Medical School (HMS) in accordance with NIH regulations. BS is a shareholder in Merrimack Pharmaceuticals and a board member of SilverCreek Pharmaceuticals.

## Authors’ contributions

MN, EAP, MC, DHC, and LZ performed all experiments. MH performed all analysis. MN, MH, and JLM designed the website; PKS and BS supervised the work and preparation of the manuscript. All authors read and approved the final manuscript.

## Supplementary Material

Additional file 1: Table S1 Description of the dataset, cell lines, ligands and assays used in this study. Click here for file

Additional file 2: Table S2 Connections between ligands and receptors. Click here for file

Additional file 3 Network maps of all cell lines used in this study. Click here for file

Additional file 4: Figure S1 Correlation between selected basal measurements. Click here for file

Additional file 5 Results of kinetic clustering and dose-dependence classification. Click here for file

Additional file 6: Figure S2 Distribution of responses and their fold-change according to the kinetic clusters and dose-dependence and distribution among sensitivity classes of response fold-change. Click here for file

Additional file 7: Figure S3 Enrichment for basal RTK levels among cell lines with significant responses. Click here for file

Additional file 8: Figure S4 Relationship between basal pErbB2, PI3K/PTEN mutational status and the basal levels of pAKT and pERK. Click here for file
