# Supplementary figures and images for: Analysis of growth factor signaling in genetically diverse breast cancer lines
Source: BMC Biol. 2014 Mar 21;12:20. doi: 10.1186/1741-7007-12-20 (PMC4234128; doi:10.1186/1741-7007-12-20)

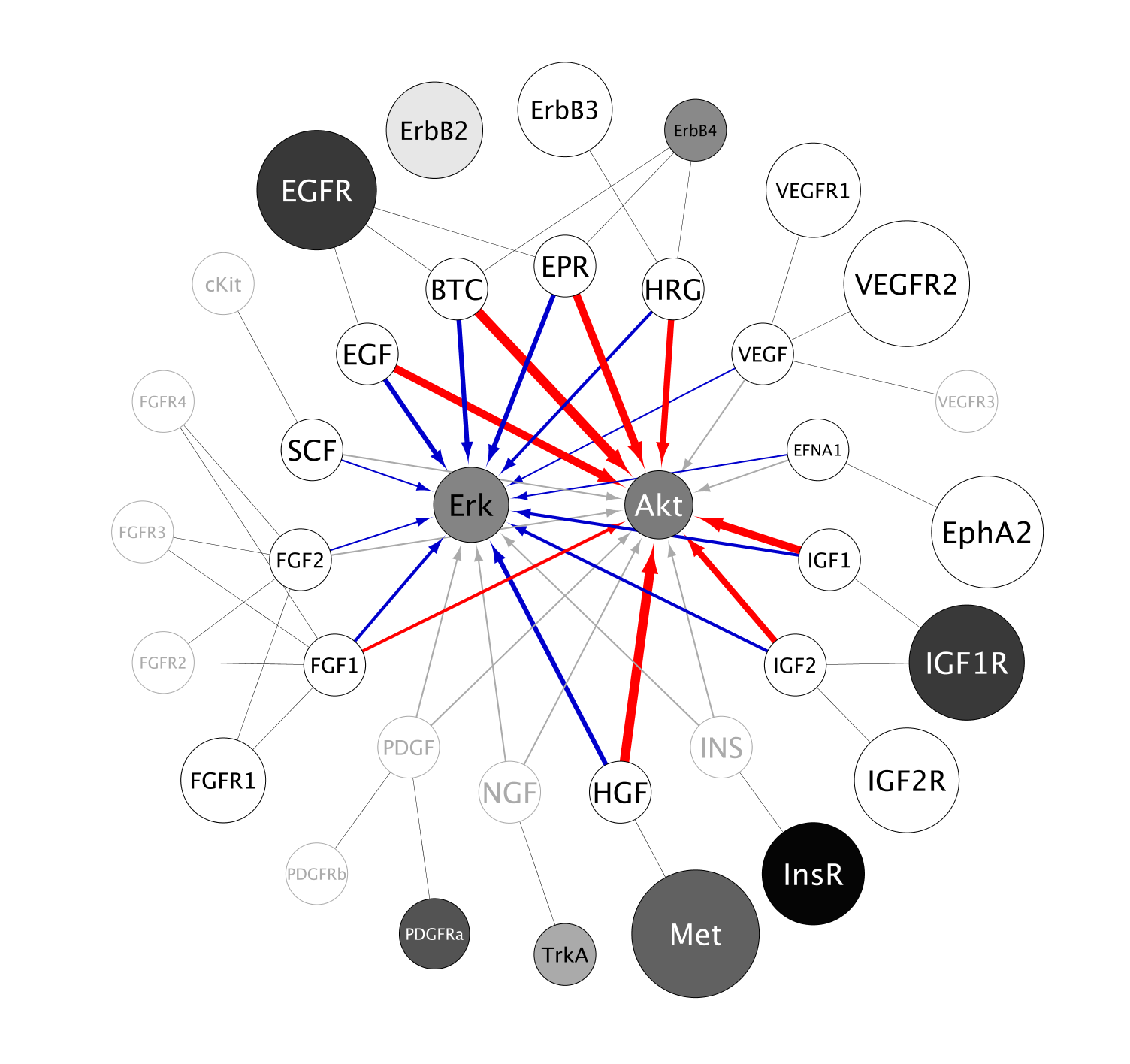

Supplement: Additional file 3 — Network maps of all cell lines used in this study. [file 1741-7007-12-20-S3.zip › 184B5.png]

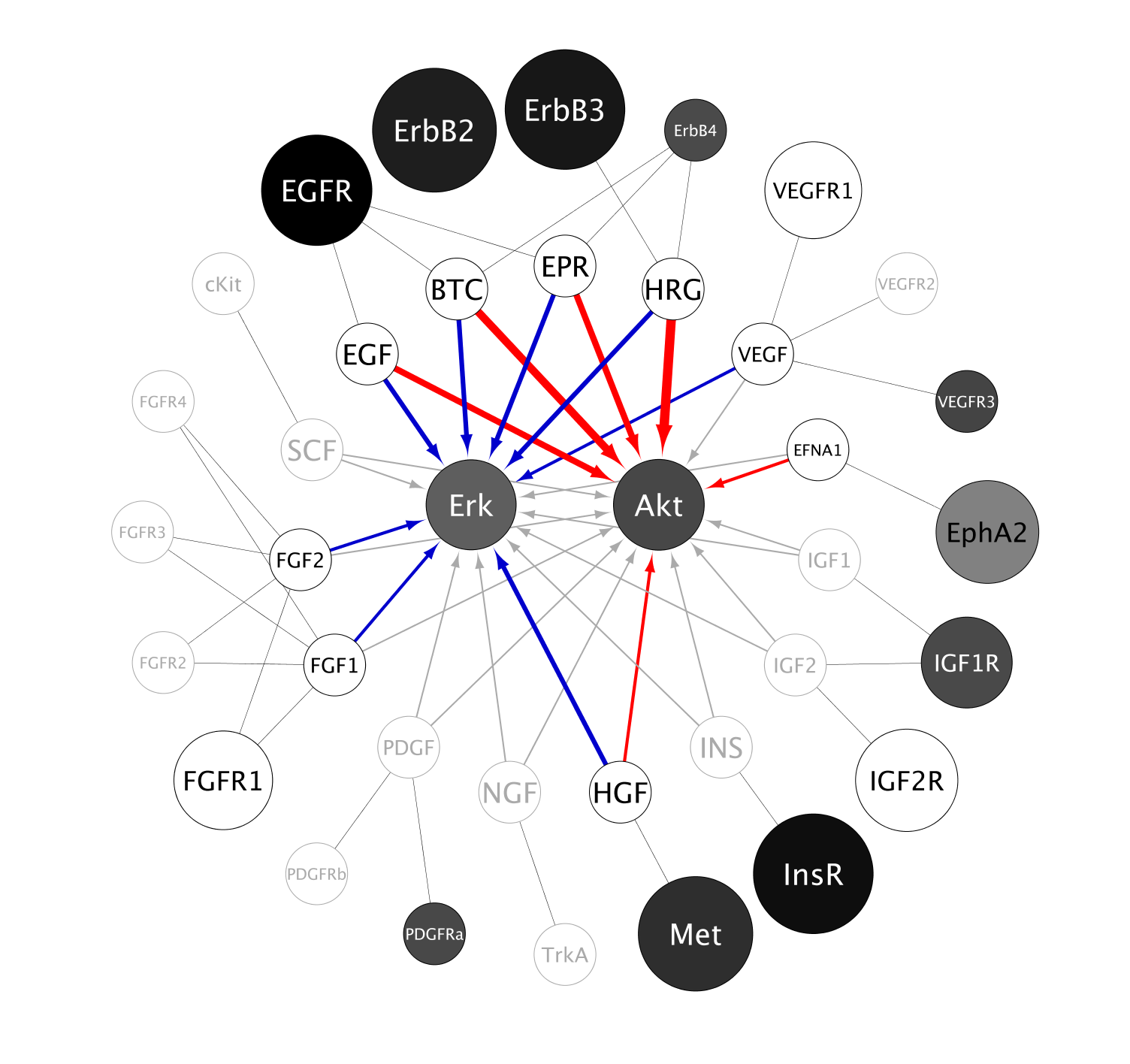

Supplement: Additional file 3 — Network maps of all cell lines used in this study. [file 1741-7007-12-20-S3.zip › AU-565.png]

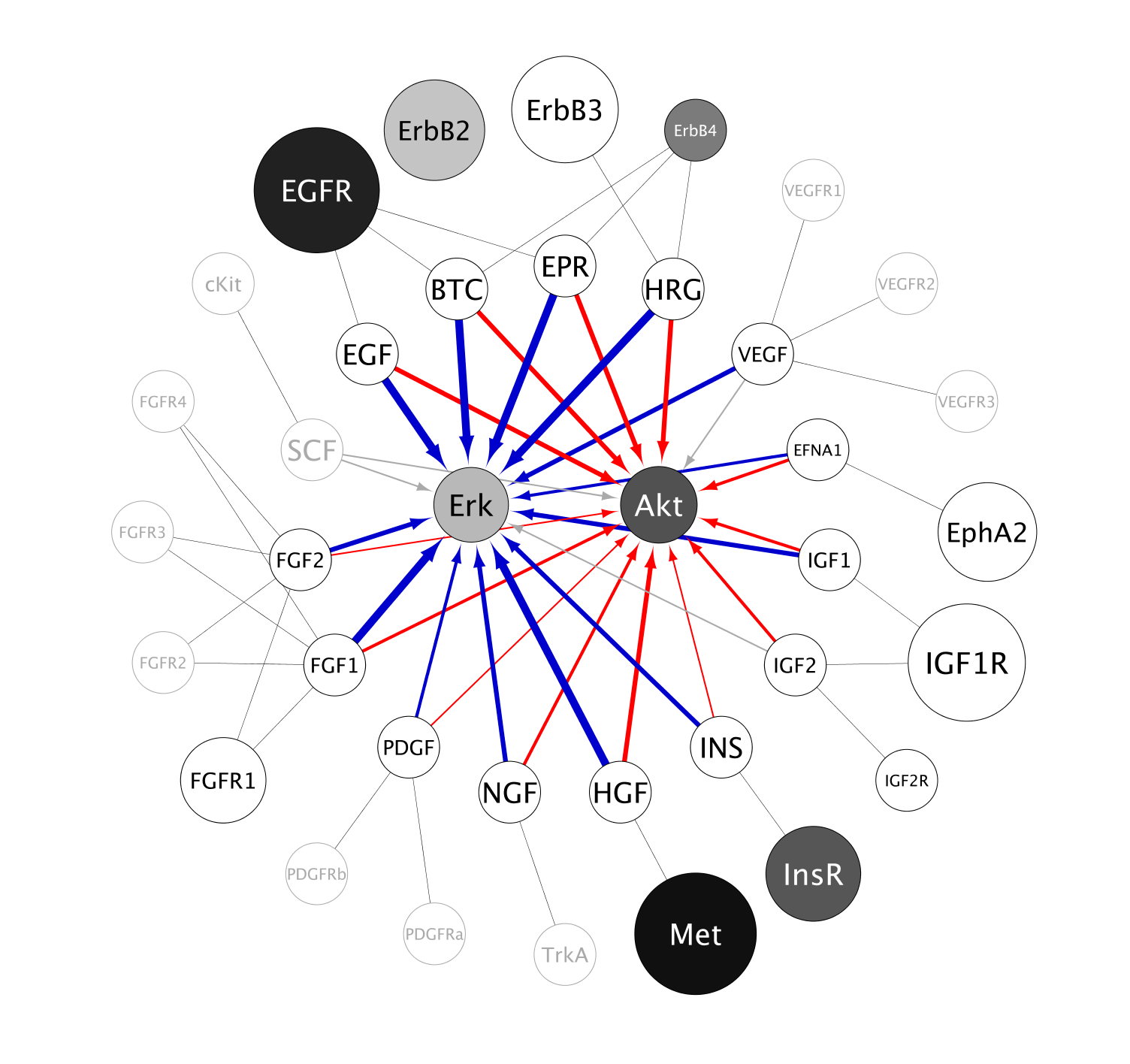

Supplement: Additional file 3 — Network maps of all cell lines used in this study. [file 1741-7007-12-20-S3.zip › BT-20.png]

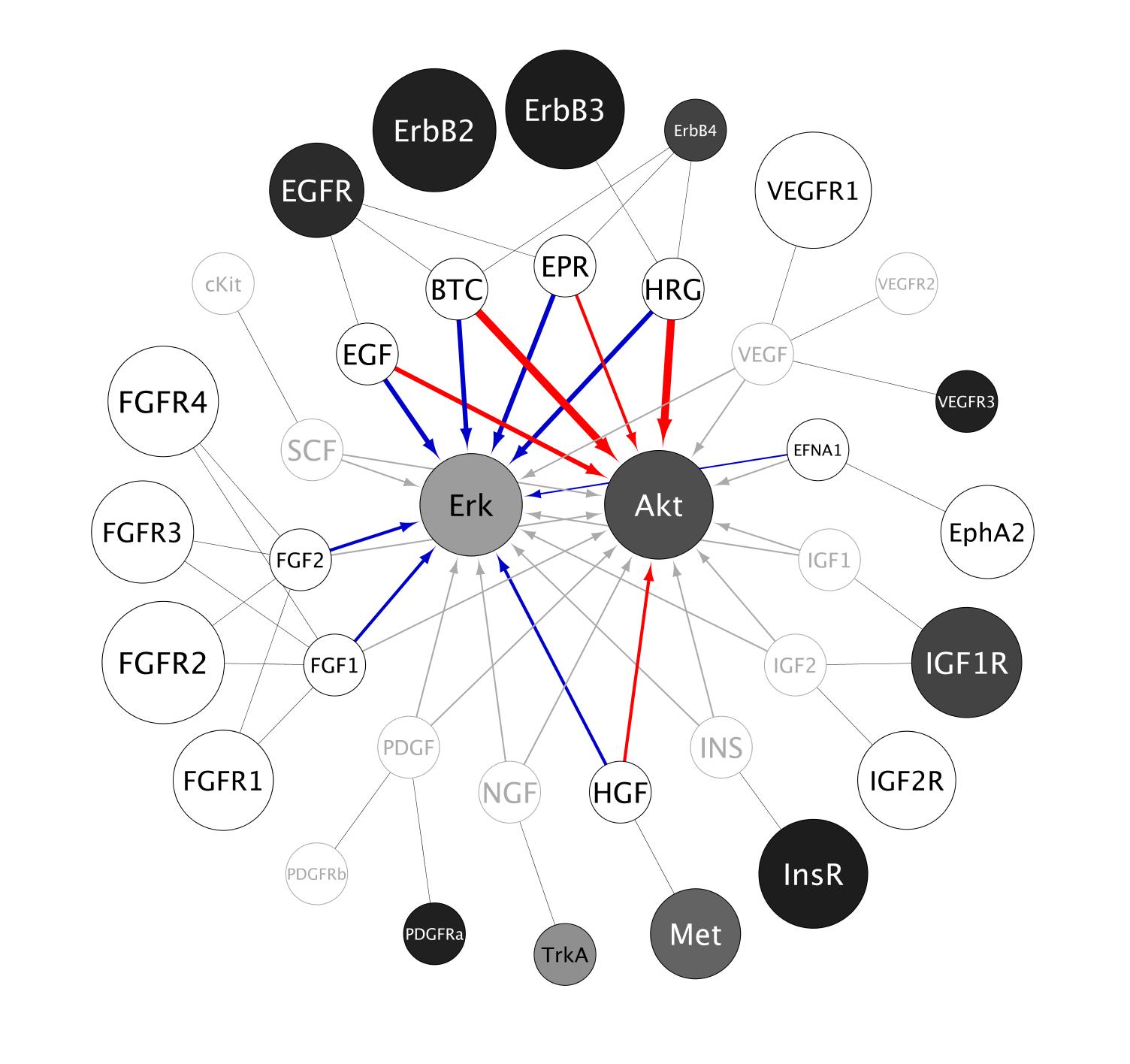

Supplement: Additional file 3 — Network maps of all cell lines used in this study. [file 1741-7007-12-20-S3.zip › BT-474.png]

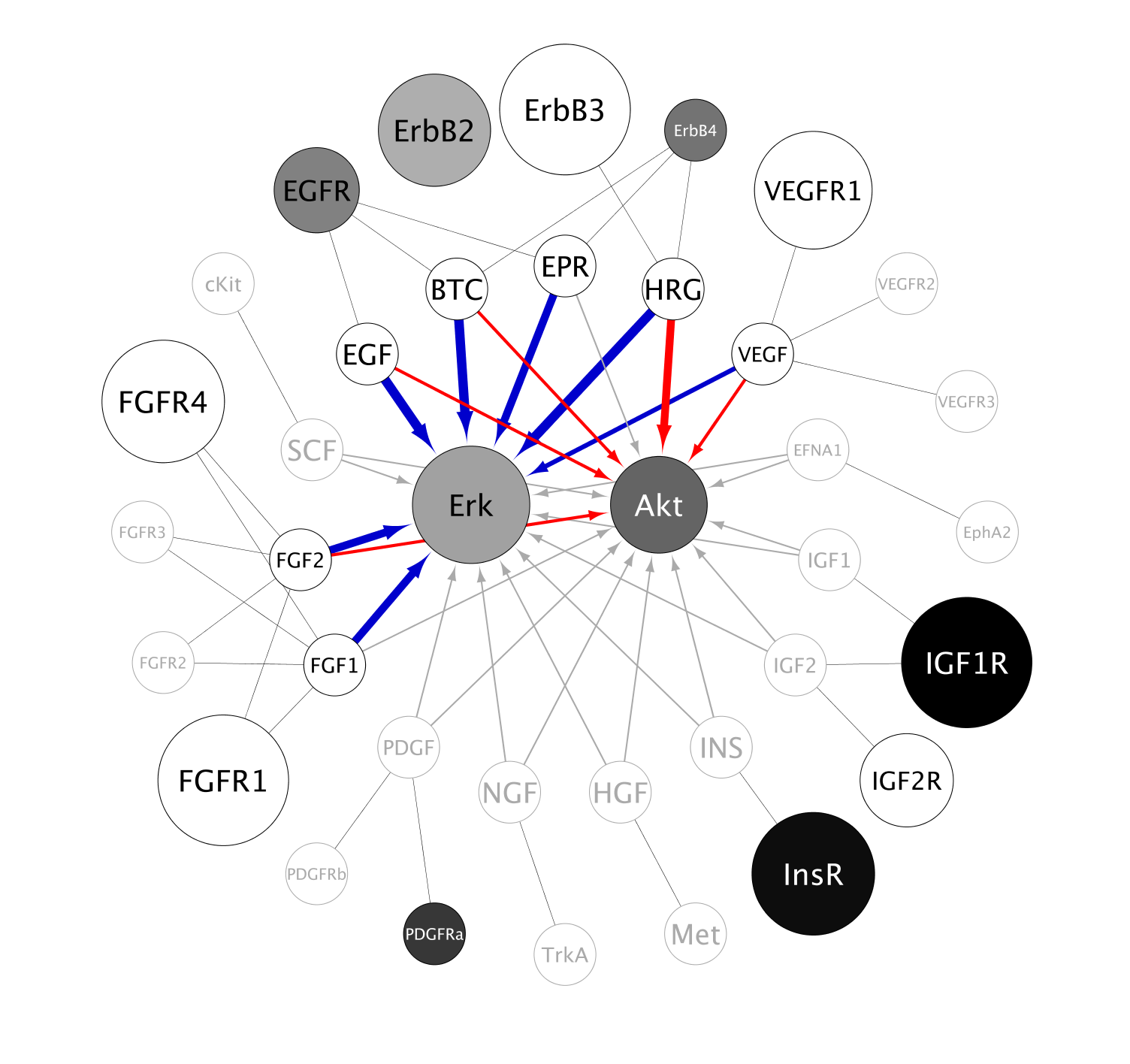

Supplement: Additional file 3 — Network maps of all cell lines used in this study. [file 1741-7007-12-20-S3.zip › BT-483.png]

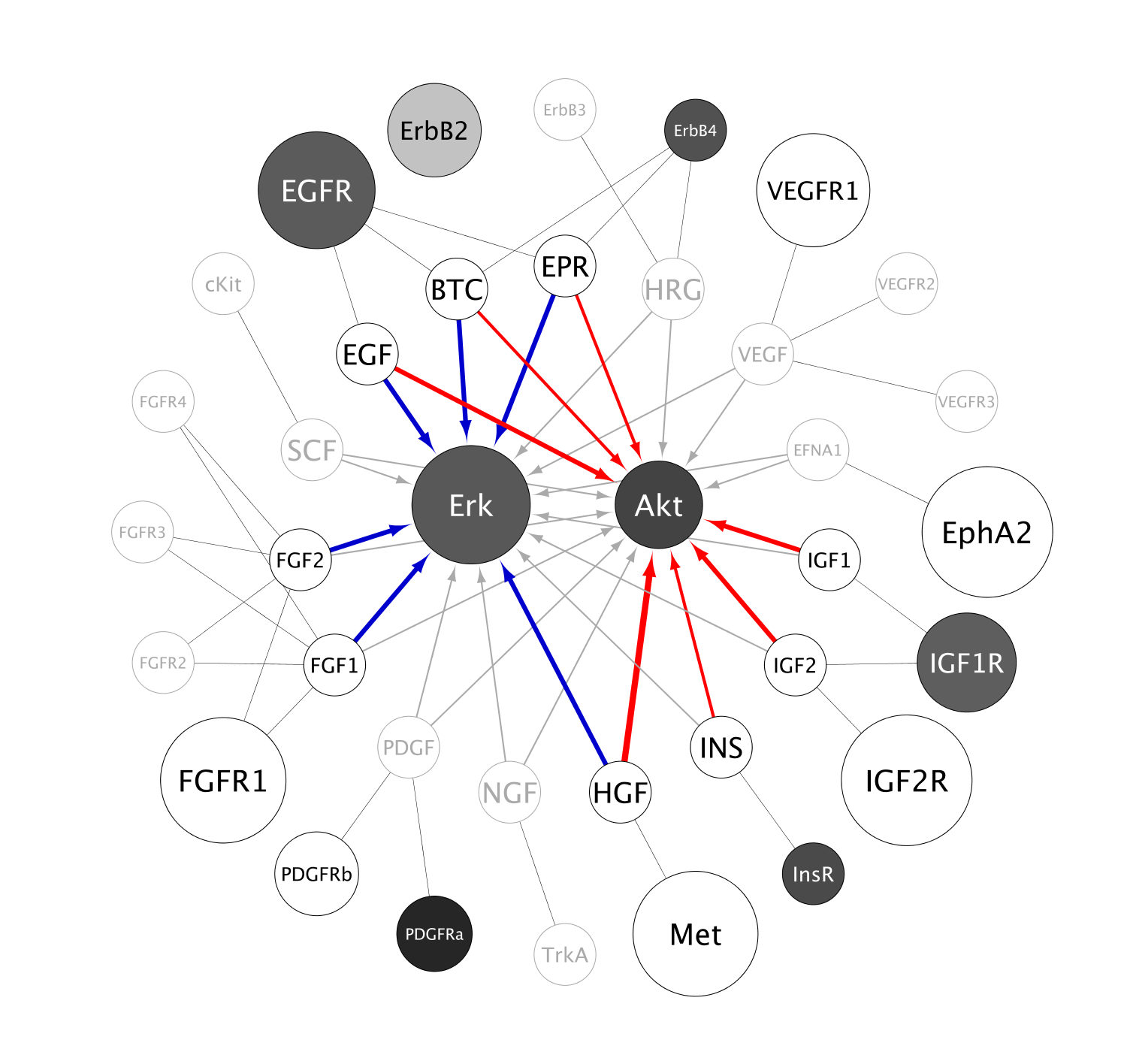

Supplement: Additional file 3 — Network maps of all cell lines used in this study. [file 1741-7007-12-20-S3.zip › BT-549.png]

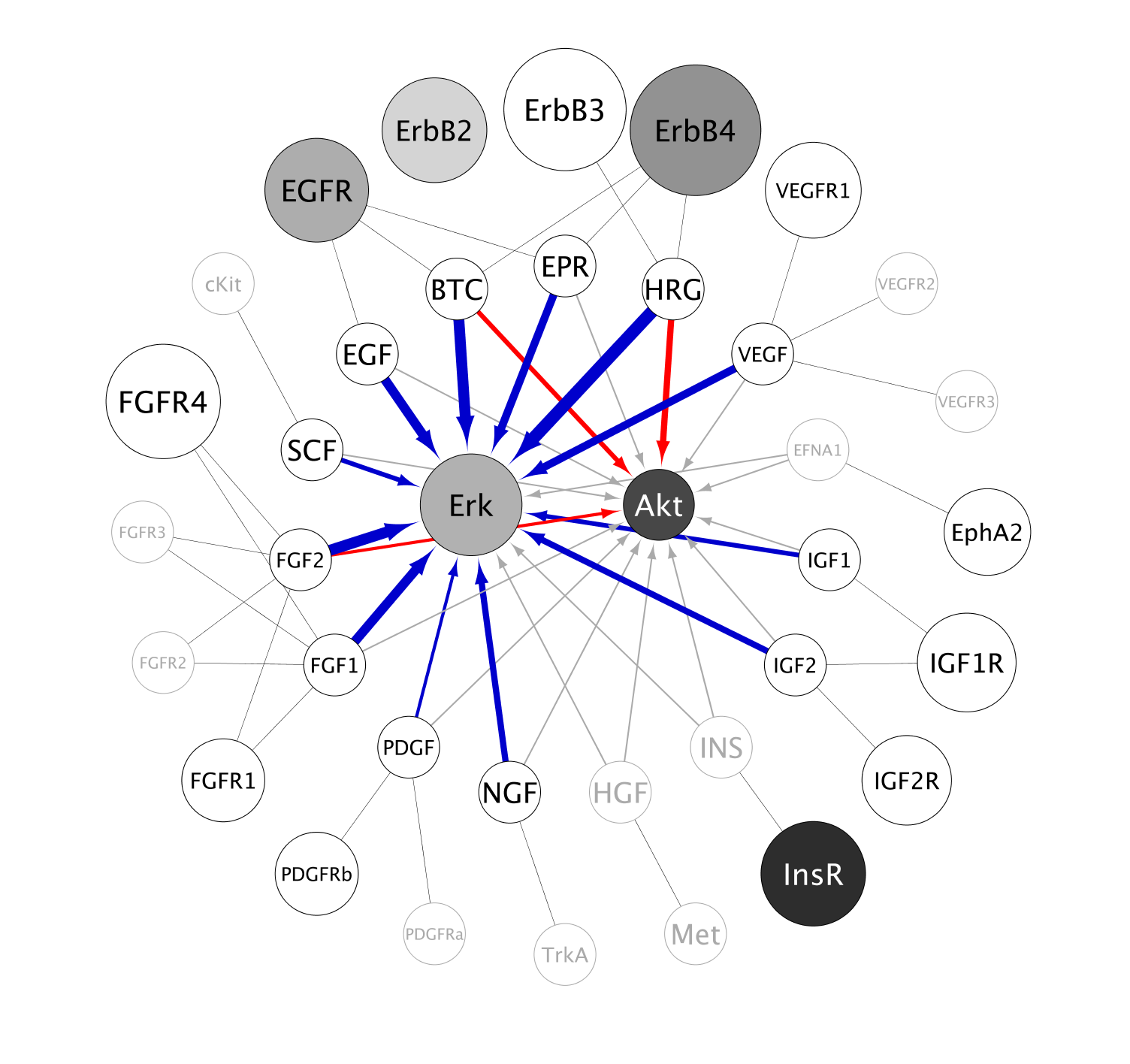

Supplement: Additional file 3 — Network maps of all cell lines used in this study. [file 1741-7007-12-20-S3.zip › CAMA-1.png]

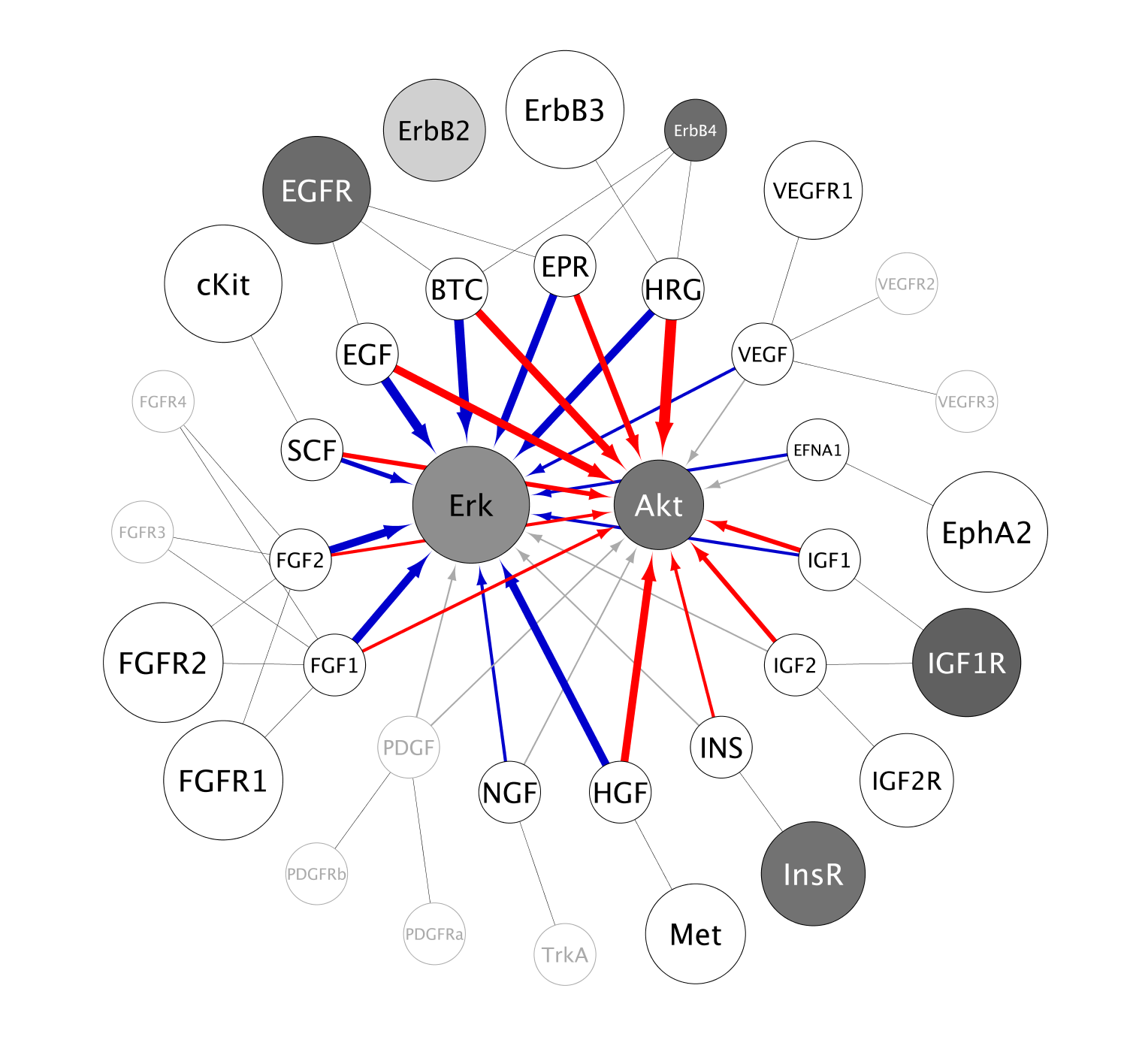

Supplement: Additional file 3 — Network maps of all cell lines used in this study. [file 1741-7007-12-20-S3.zip › HCC38.png]

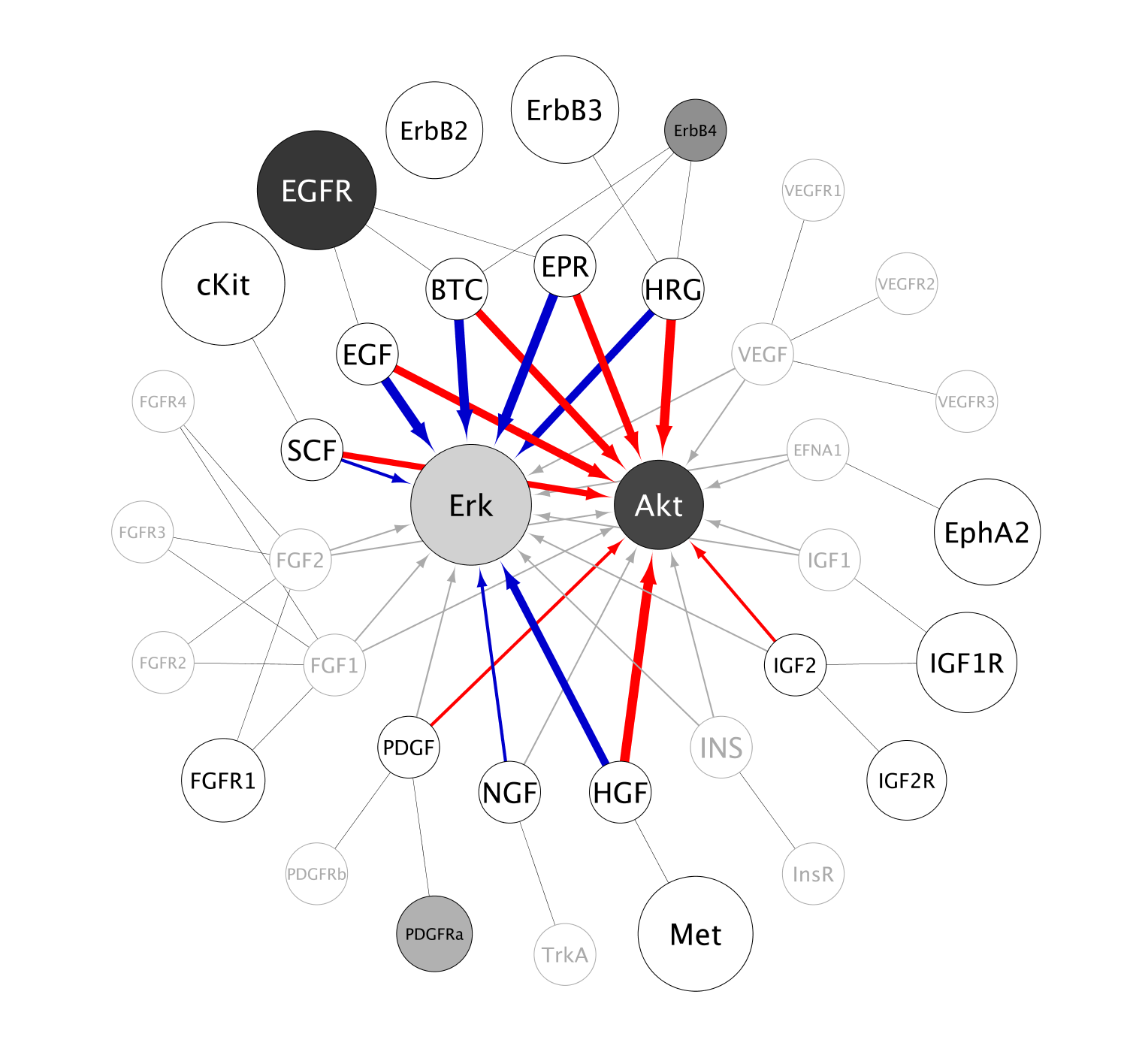

Supplement: Additional file 3 — Network maps of all cell lines used in this study. [file 1741-7007-12-20-S3.zip › HCC70.png]

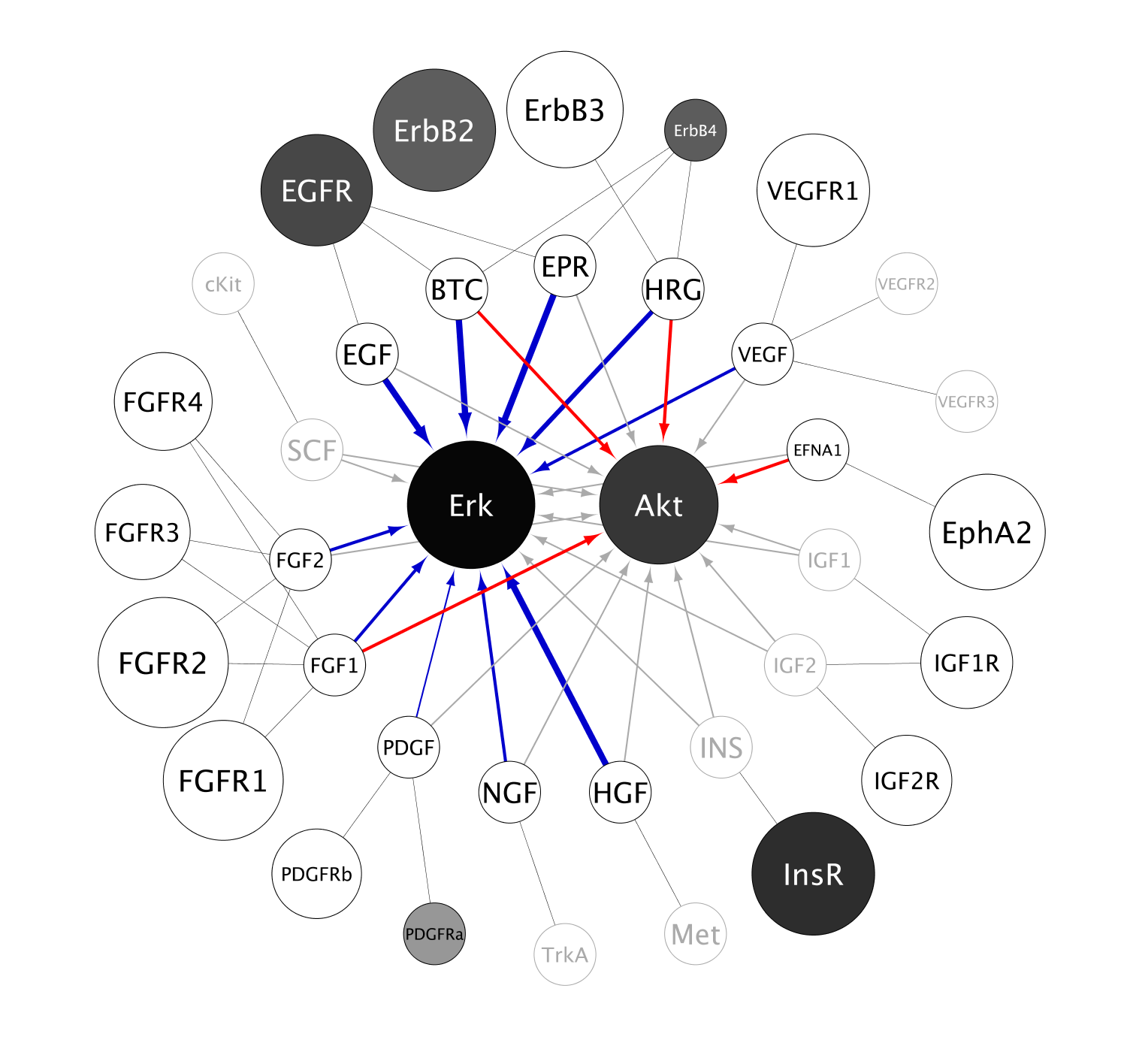

Supplement: Additional file 3 — Network maps of all cell lines used in this study. [file 1741-7007-12-20-S3.zip › HCC202.png]

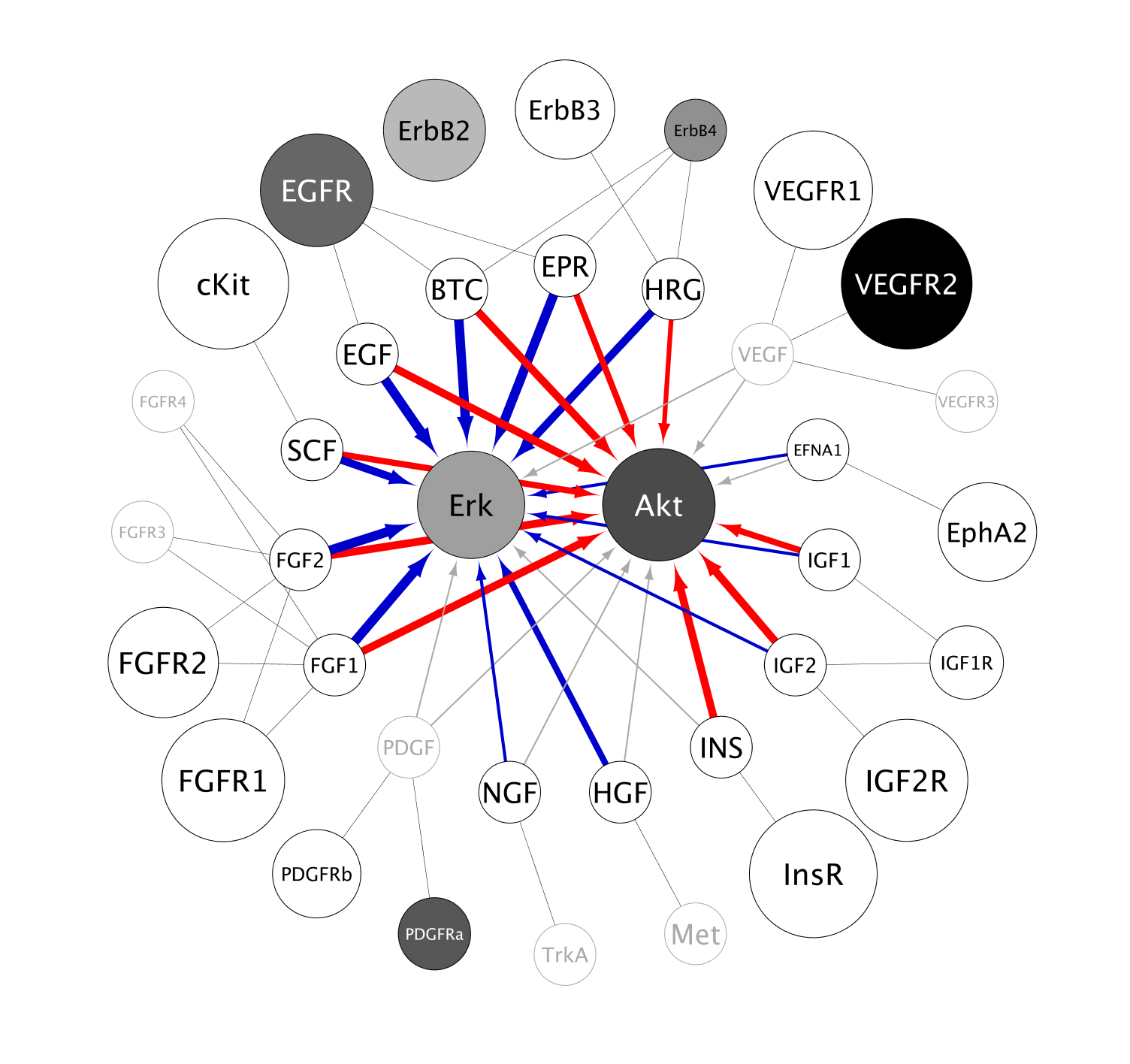

Supplement: Additional file 3 — Network maps of all cell lines used in this study. [file 1741-7007-12-20-S3.zip › HCC1187.png]

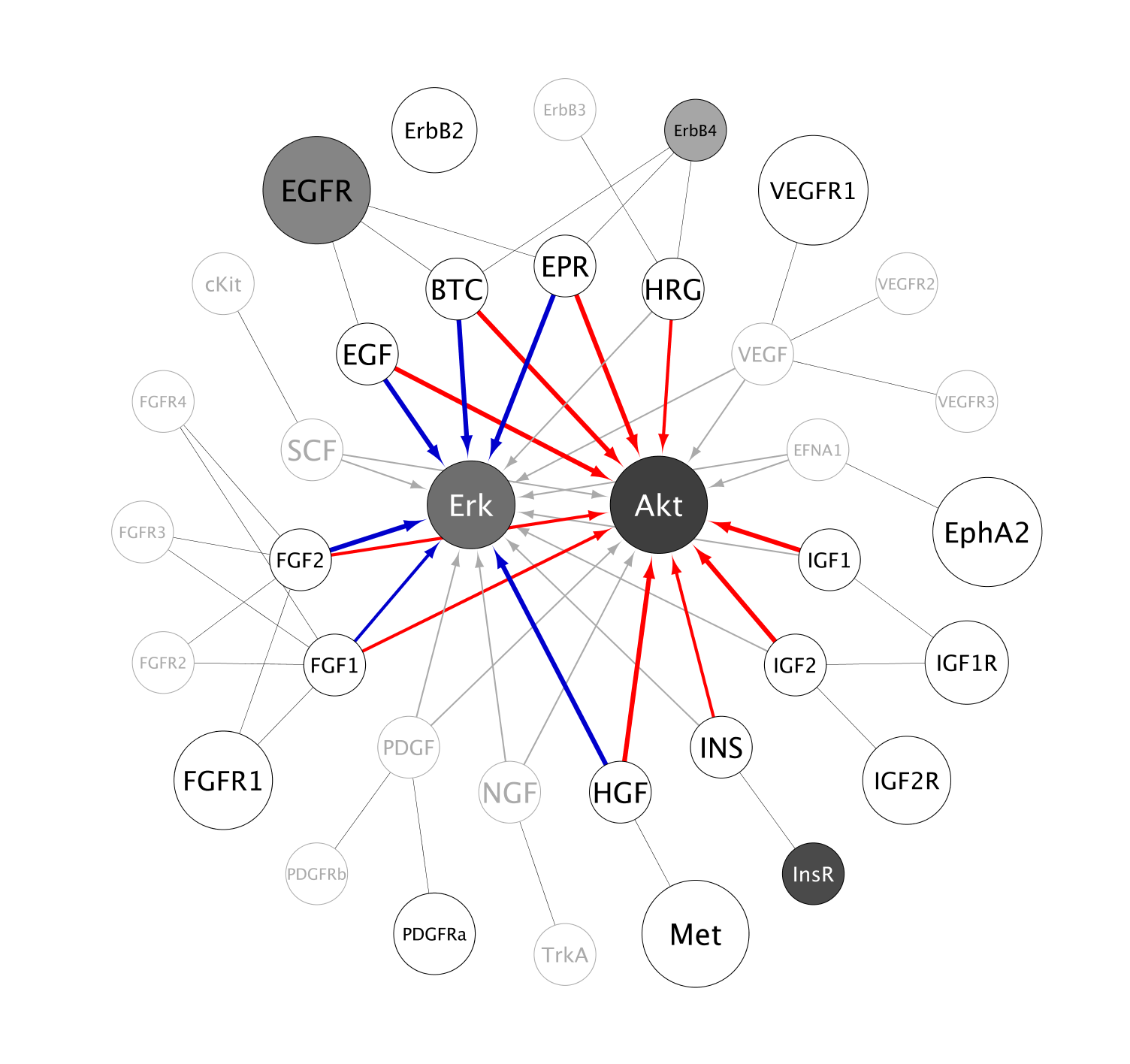

Supplement: Additional file 3 — Network maps of all cell lines used in this study. [file 1741-7007-12-20-S3.zip › HCC1395.png]

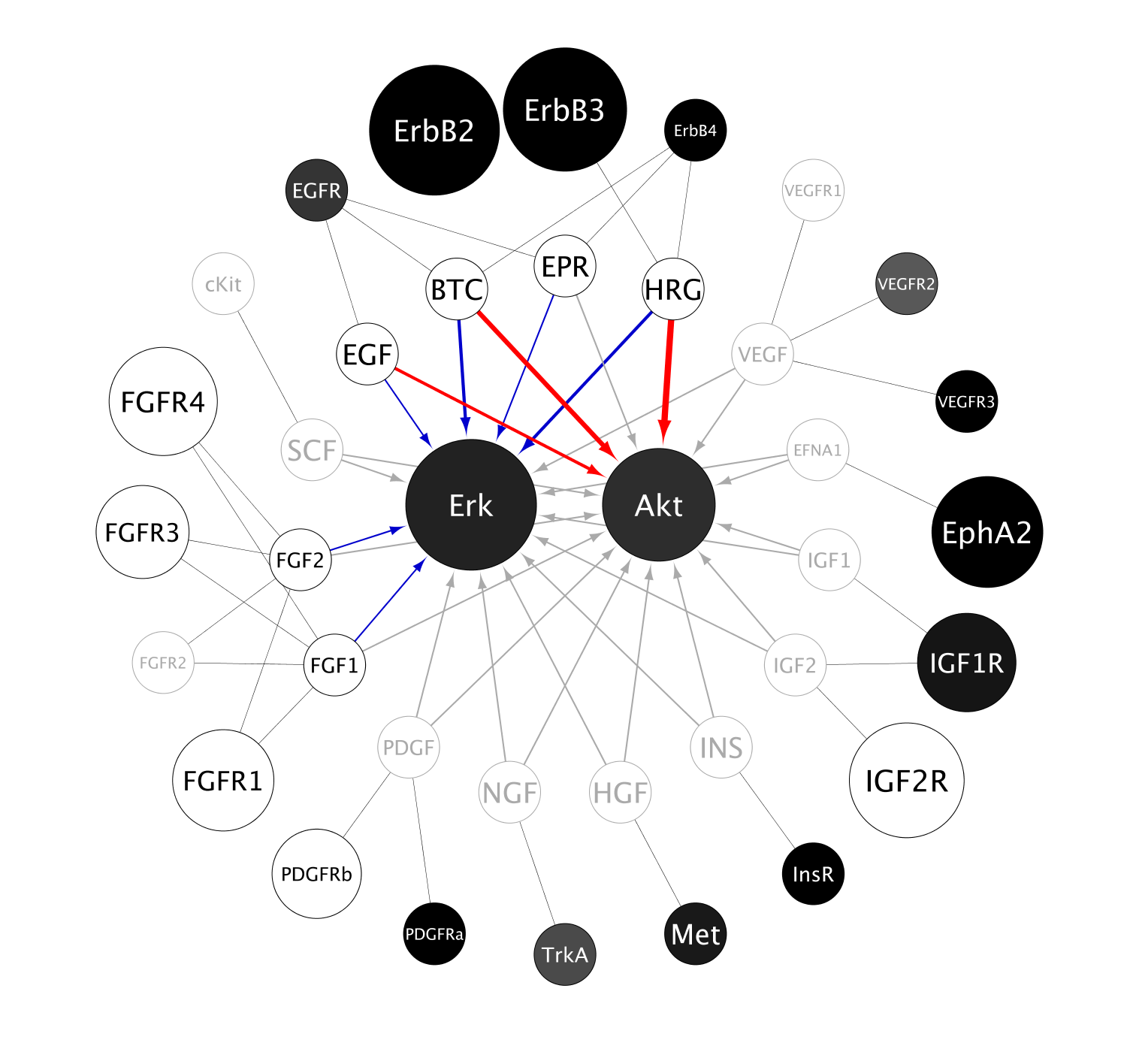

Supplement: Additional file 3 — Network maps of all cell lines used in this study. [file 1741-7007-12-20-S3.zip › HCC1419.png]

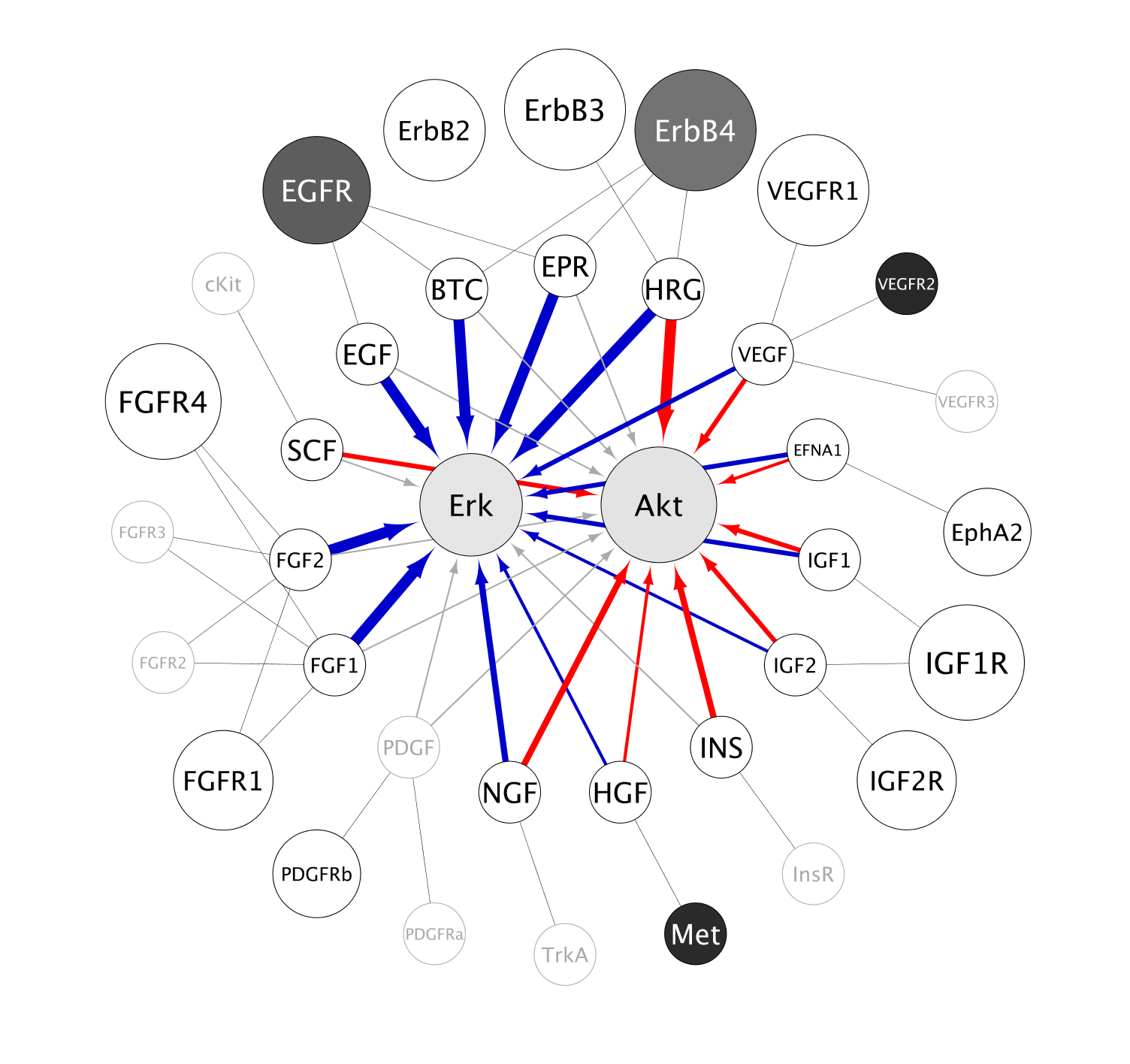

Supplement: Additional file 3 — Network maps of all cell lines used in this study. [file 1741-7007-12-20-S3.zip › HCC1428.png]

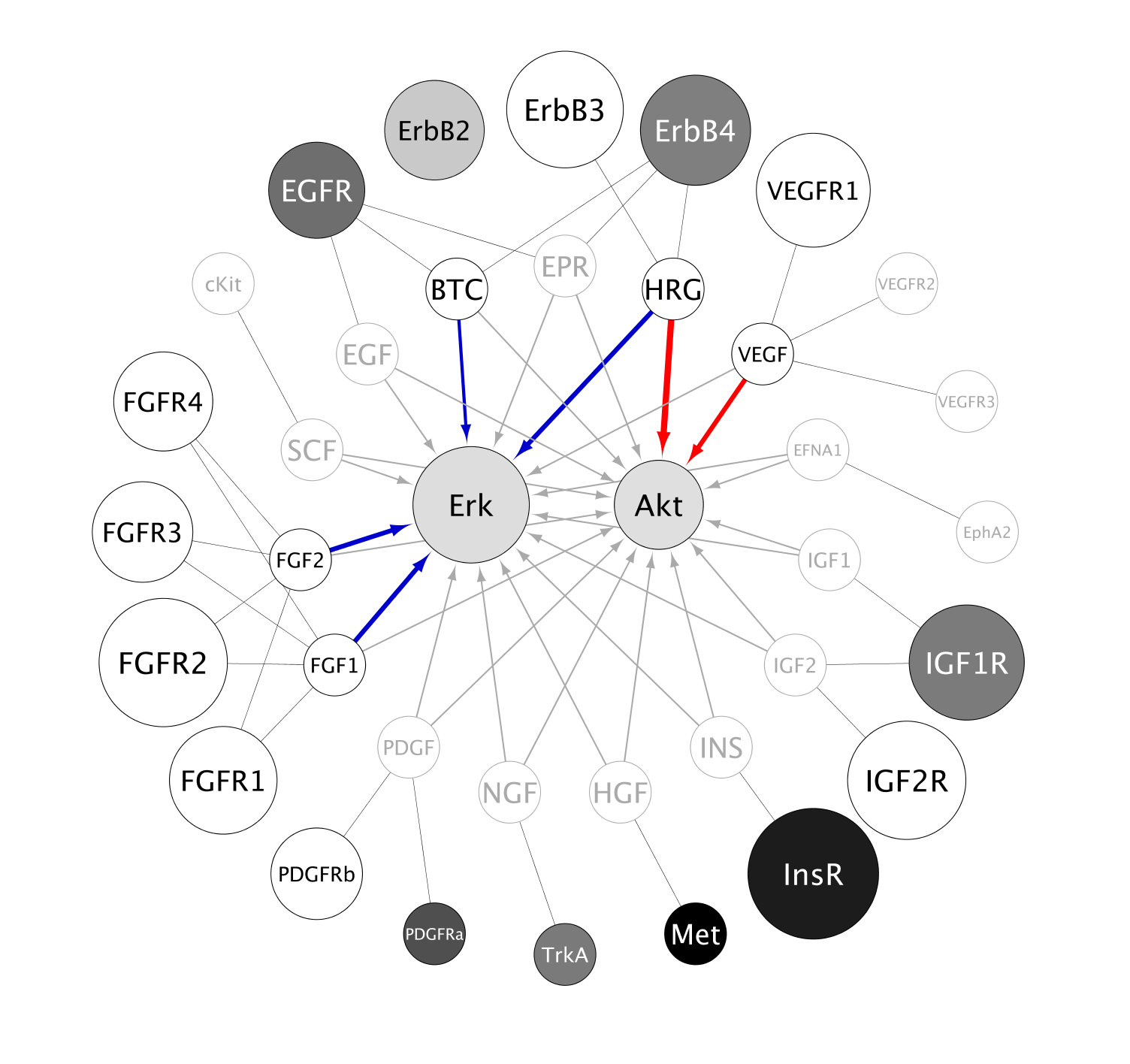

Supplement: Additional file 3 — Network maps of all cell lines used in this study. [file 1741-7007-12-20-S3.zip › HCC1500.png]

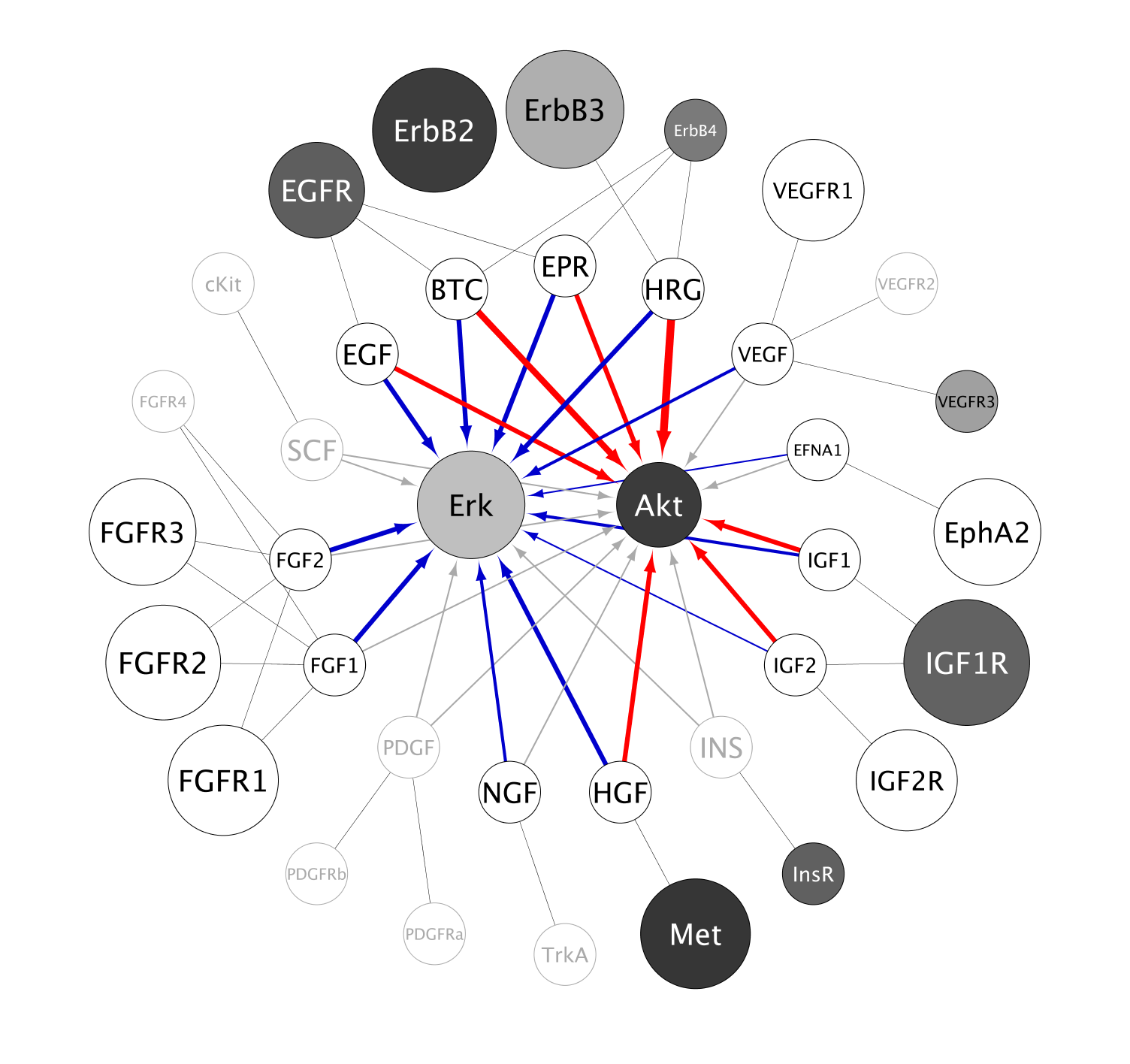

Supplement: Additional file 3 — Network maps of all cell lines used in this study. [file 1741-7007-12-20-S3.zip › HCC1569.png]

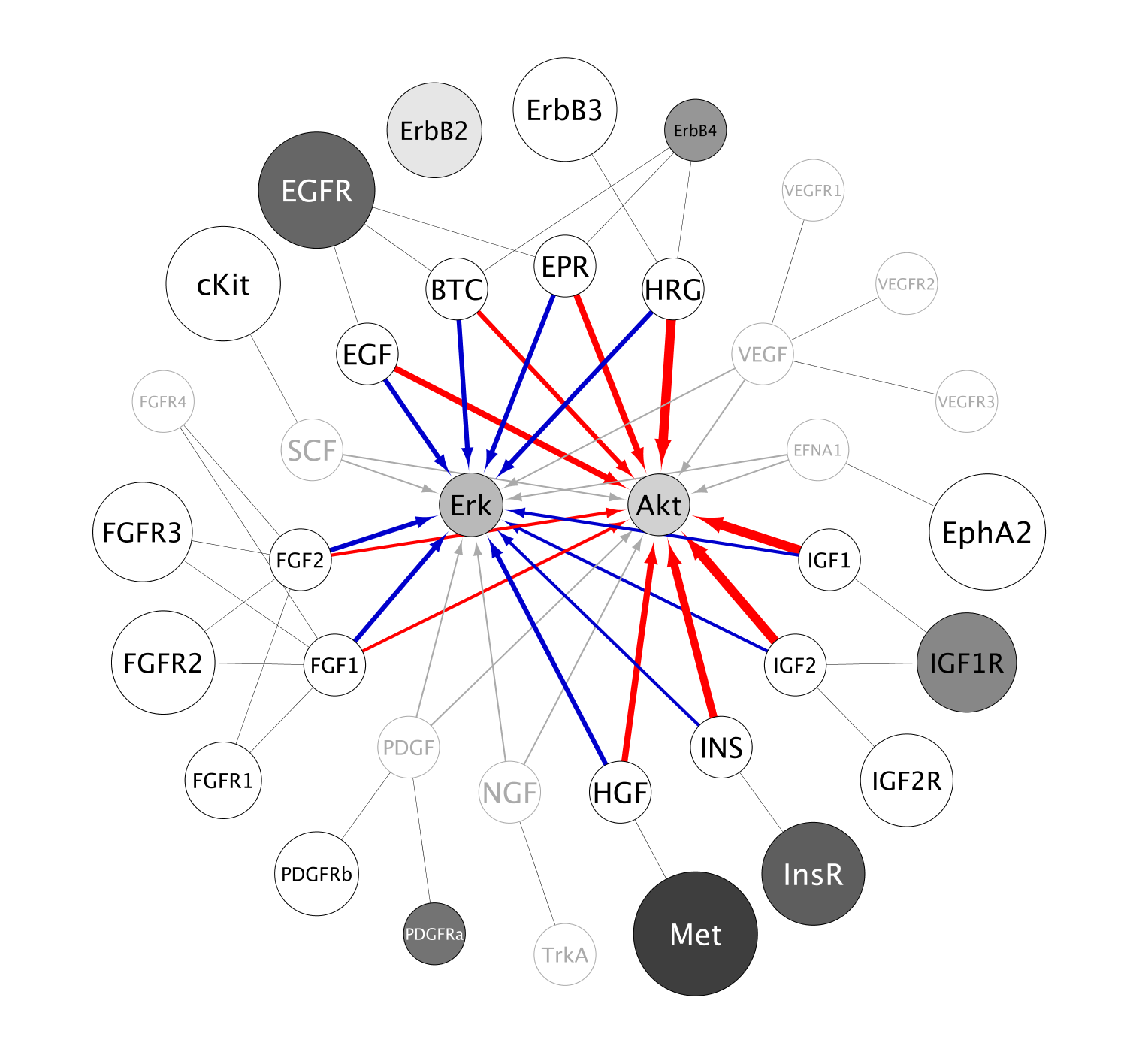

Supplement: Additional file 3 — Network maps of all cell lines used in this study. [file 1741-7007-12-20-S3.zip › HCC1806.png]

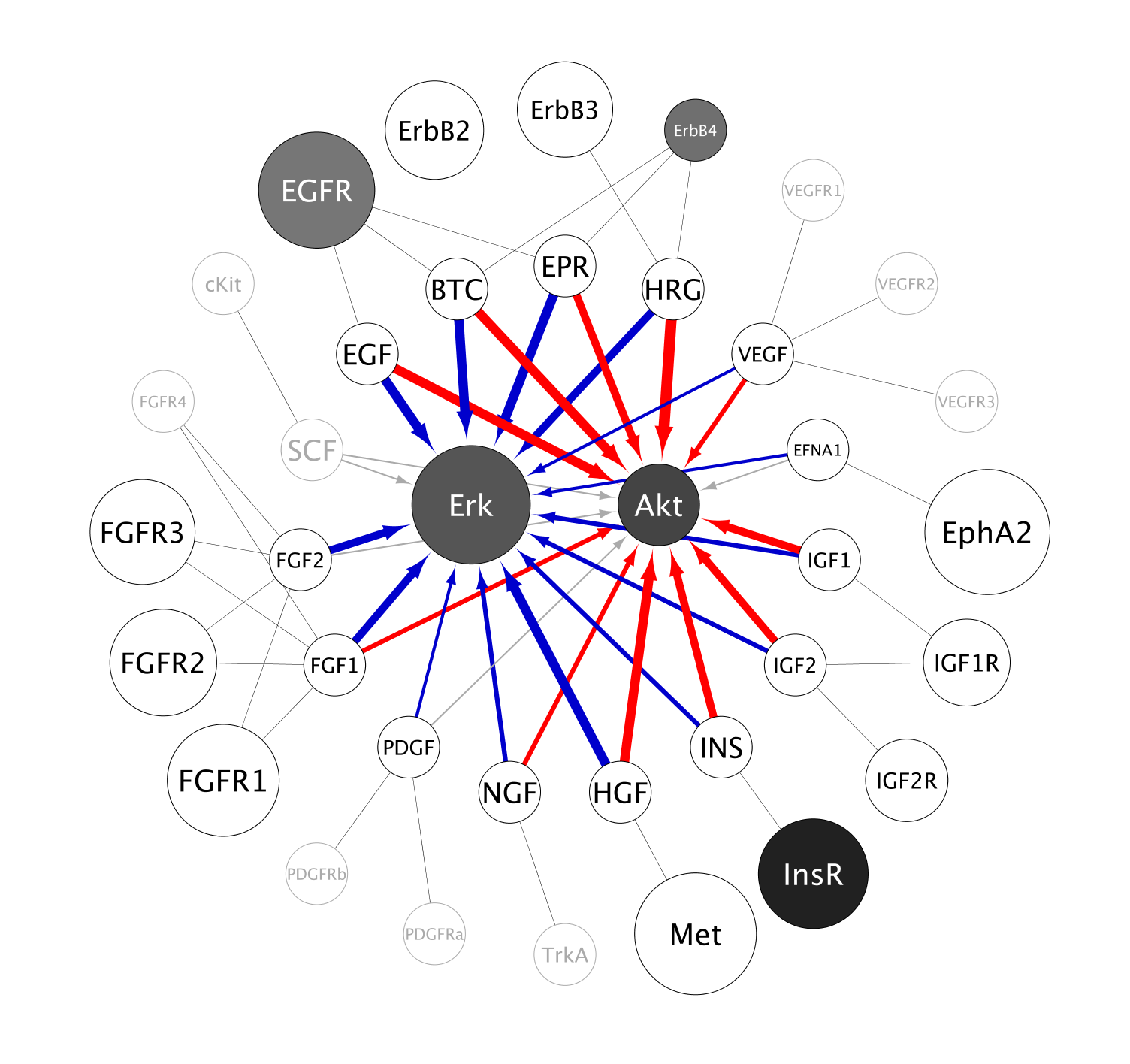

Supplement: Additional file 3 — Network maps of all cell lines used in this study. [file 1741-7007-12-20-S3.zip › HCC1937.png]

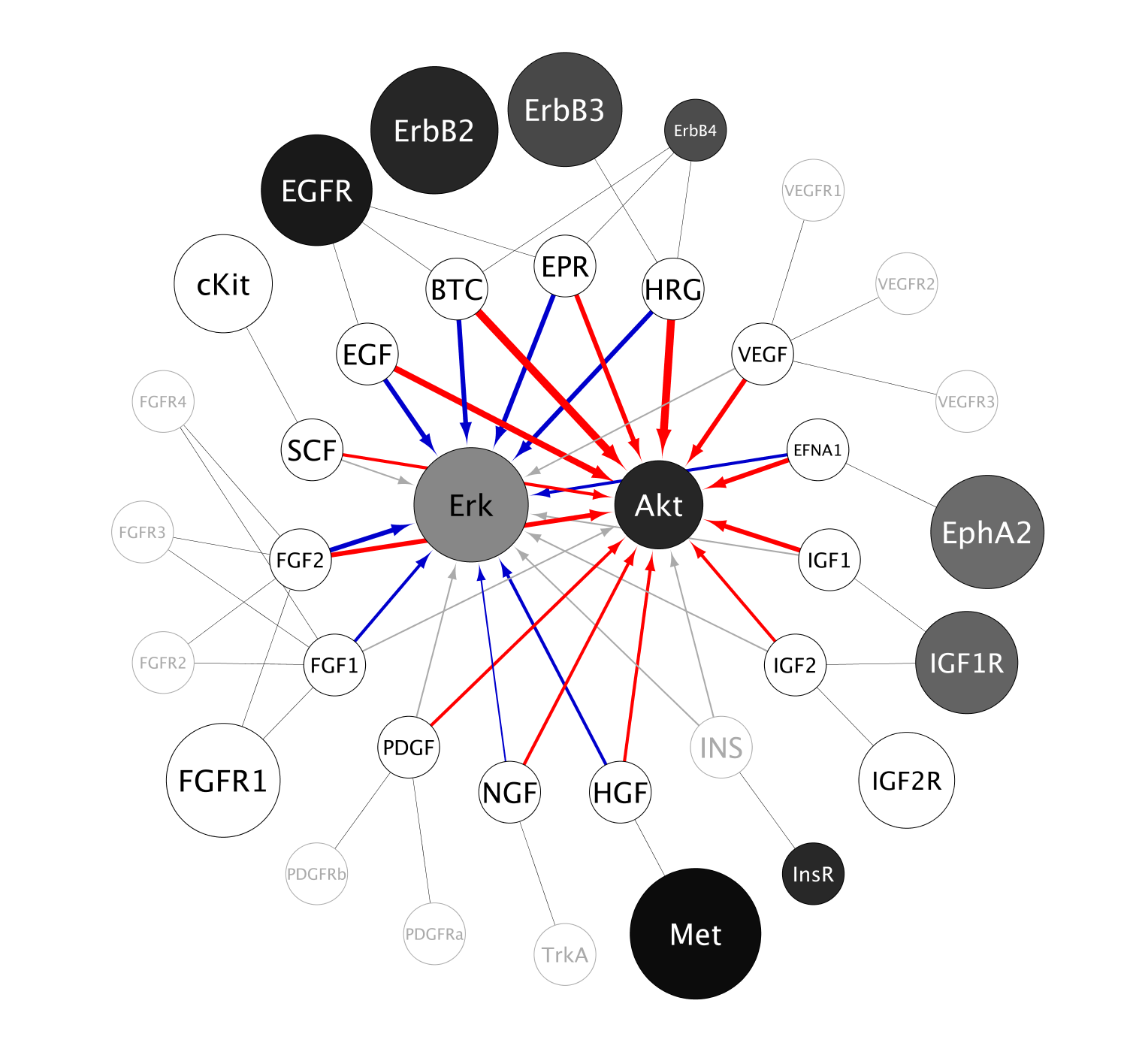

Supplement: Additional file 3 — Network maps of all cell lines used in this study. [file 1741-7007-12-20-S3.zip › HCC1954.png]

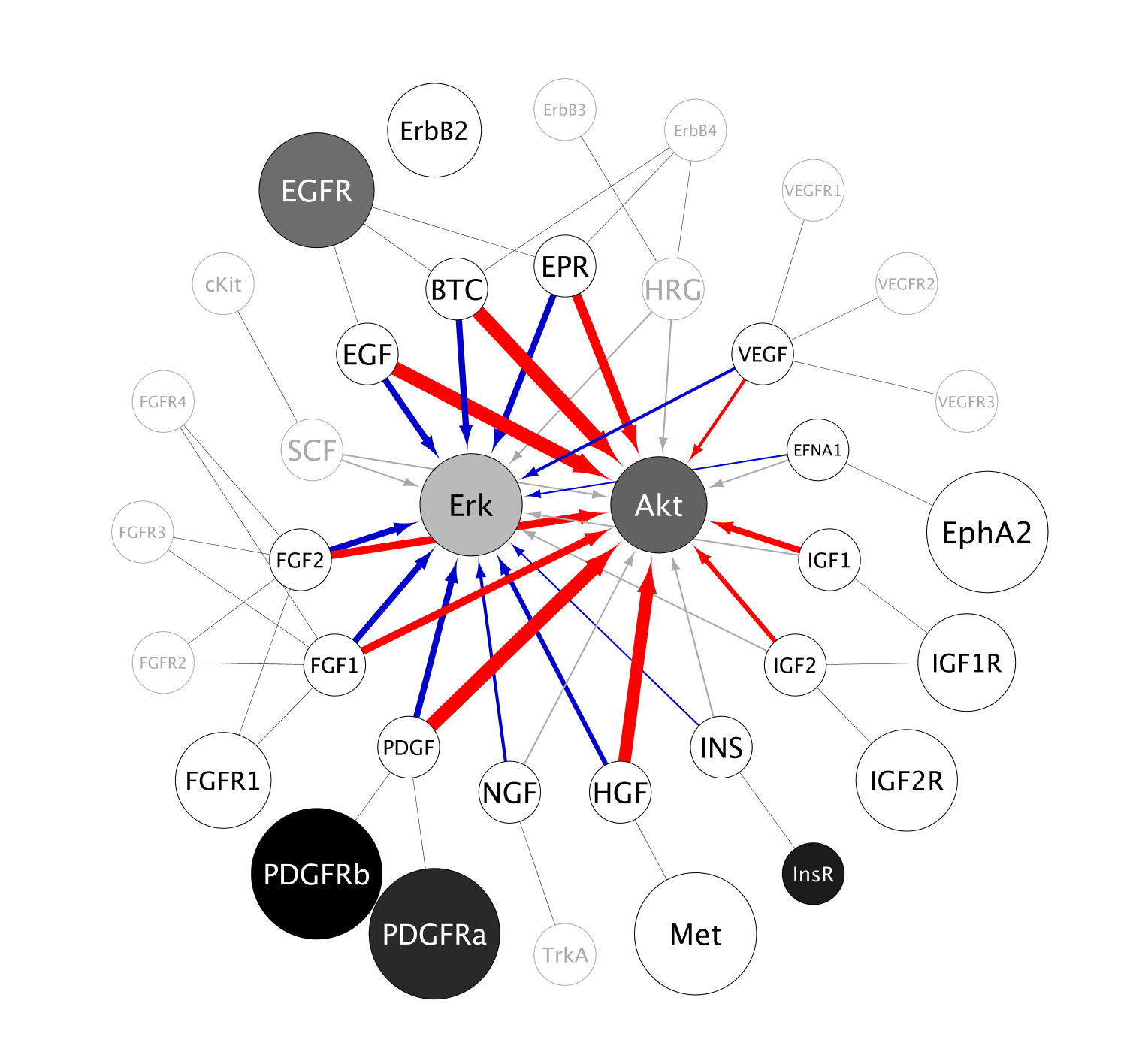

Supplement: Additional file 3 — Network maps of all cell lines used in this study. [file 1741-7007-12-20-S3.zip › Hs 578T.png]

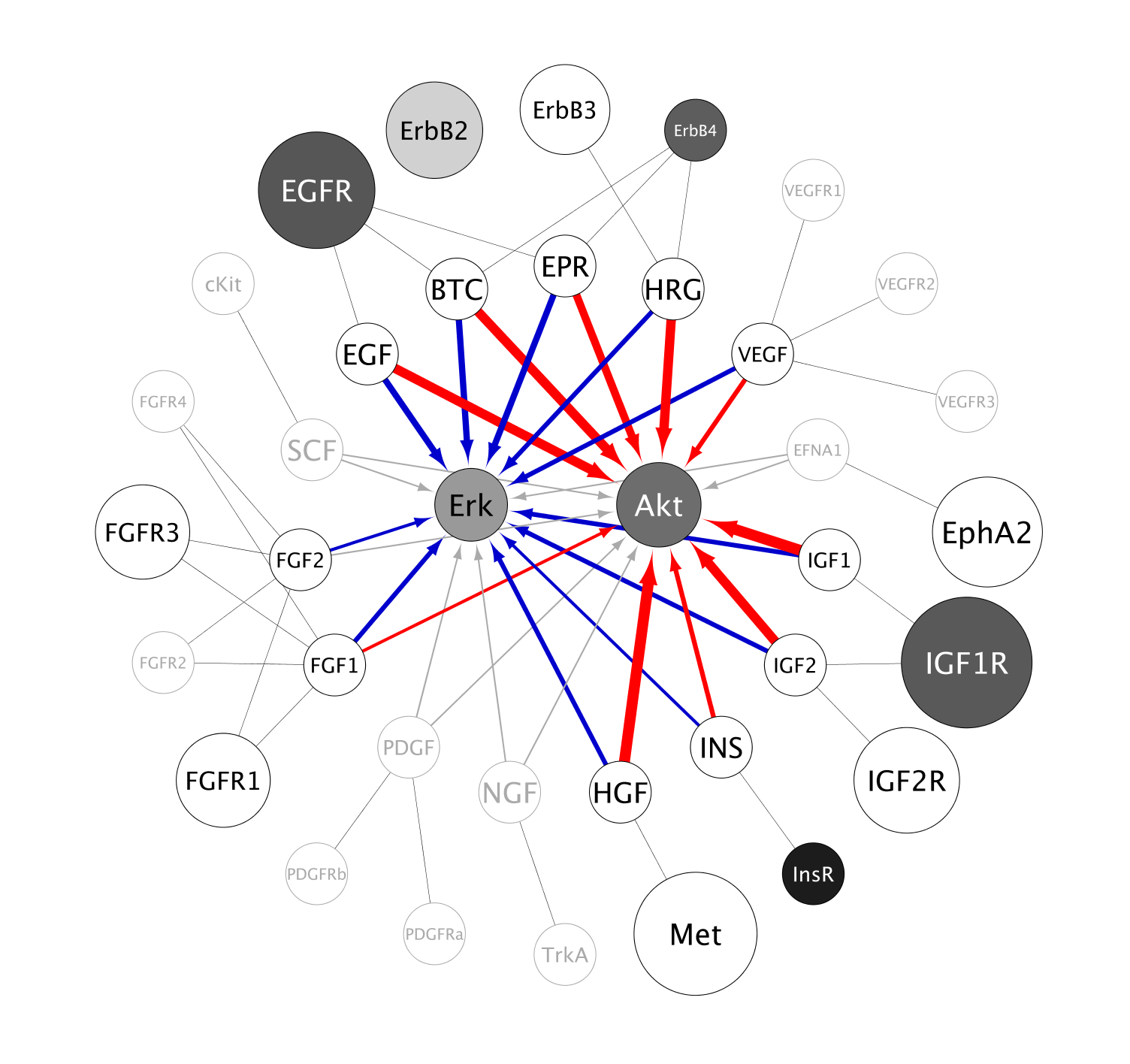

Supplement: Additional file 3 — Network maps of all cell lines used in this study. [file 1741-7007-12-20-S3.zip › MCF 10A.png]

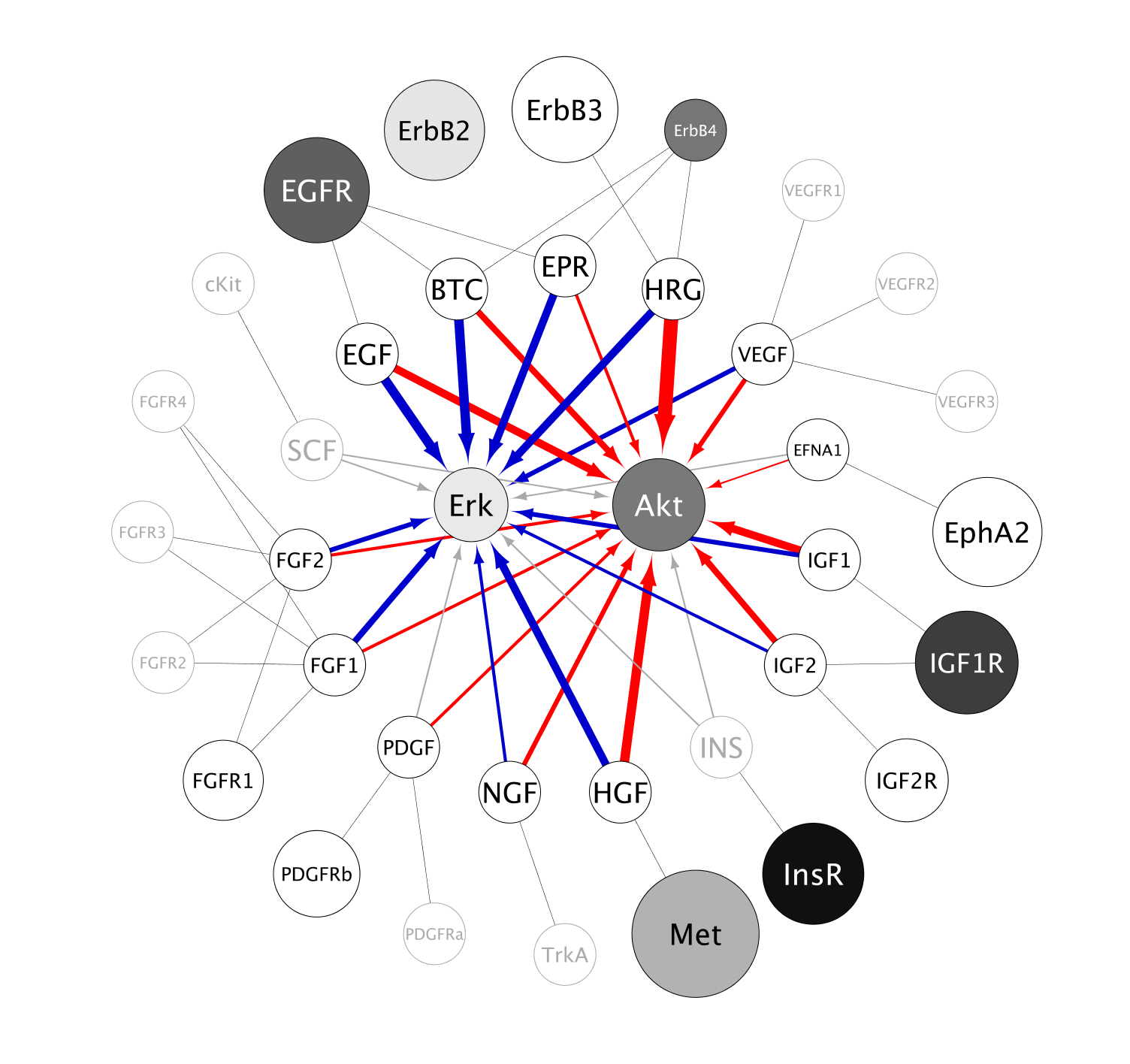

Supplement: Additional file 3 — Network maps of all cell lines used in this study. [file 1741-7007-12-20-S3.zip › MCF 10F.png]

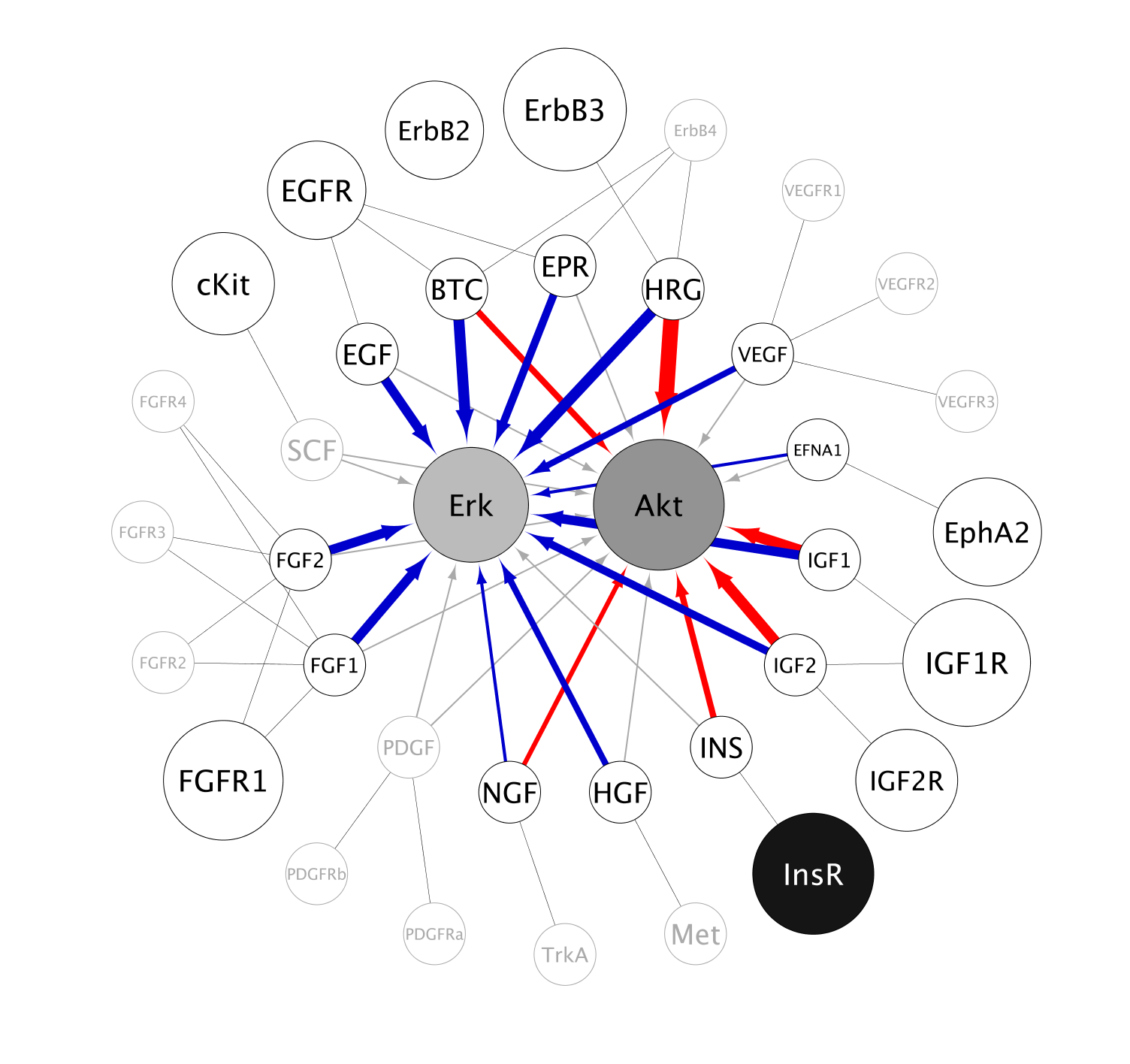

Supplement: Additional file 3 — Network maps of all cell lines used in this study. [file 1741-7007-12-20-S3.zip › MCF7.png]

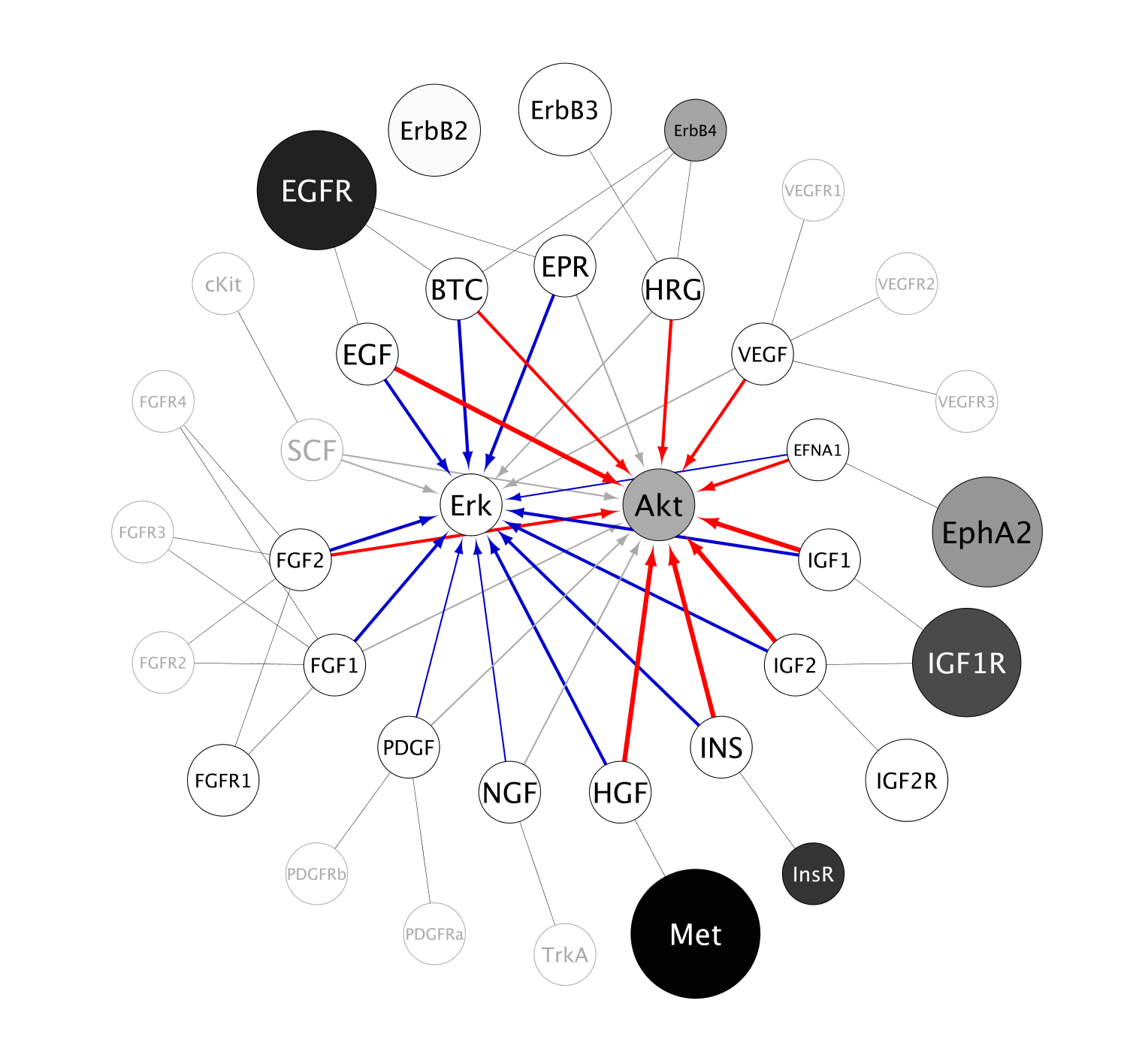

Supplement: Additional file 3 — Network maps of all cell lines used in this study. [file 1741-7007-12-20-S3.zip › MCF-12A.png]

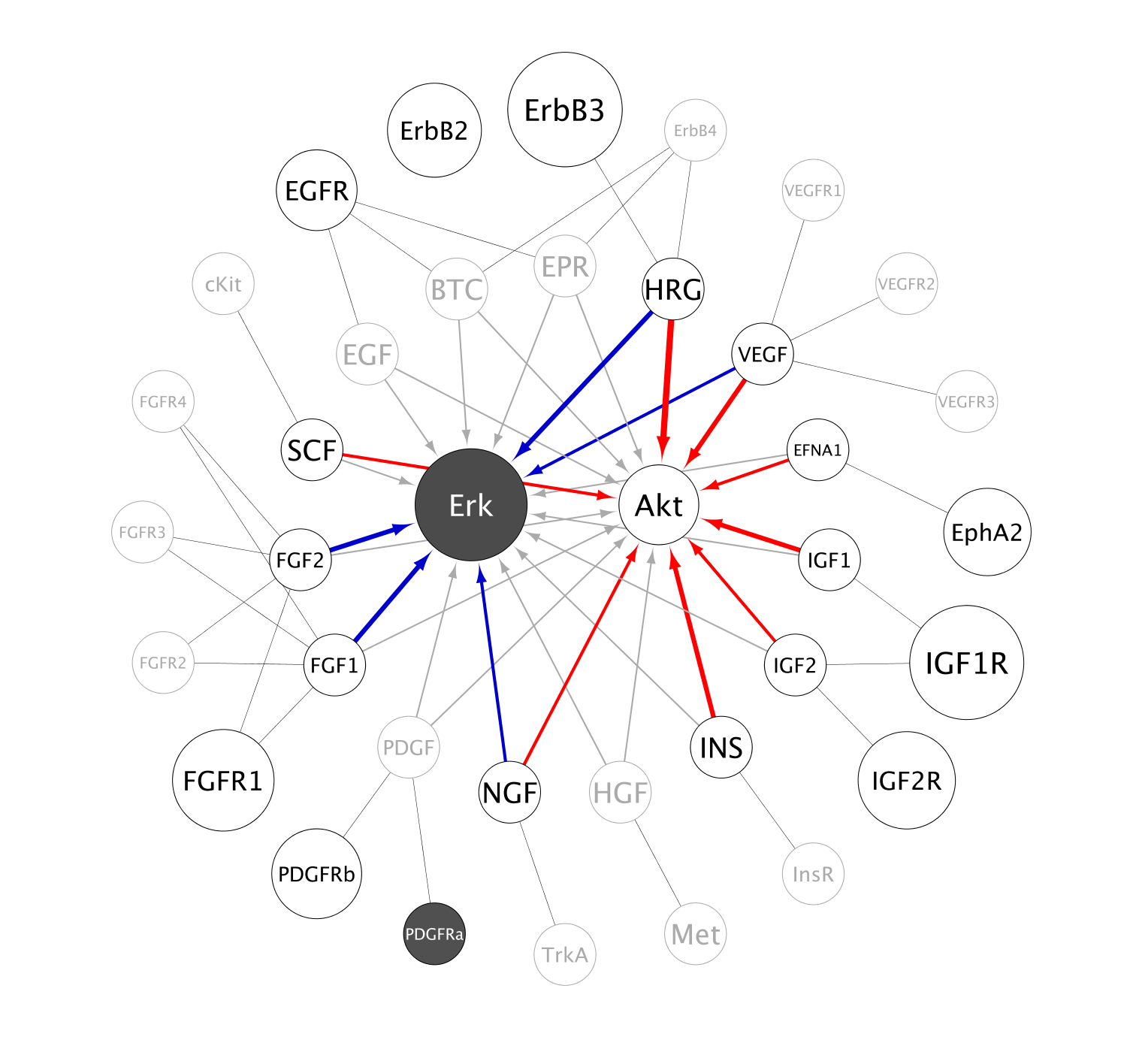

Supplement: Additional file 3 — Network maps of all cell lines used in this study. [file 1741-7007-12-20-S3.zip › MDA-MB-134-VI.png]

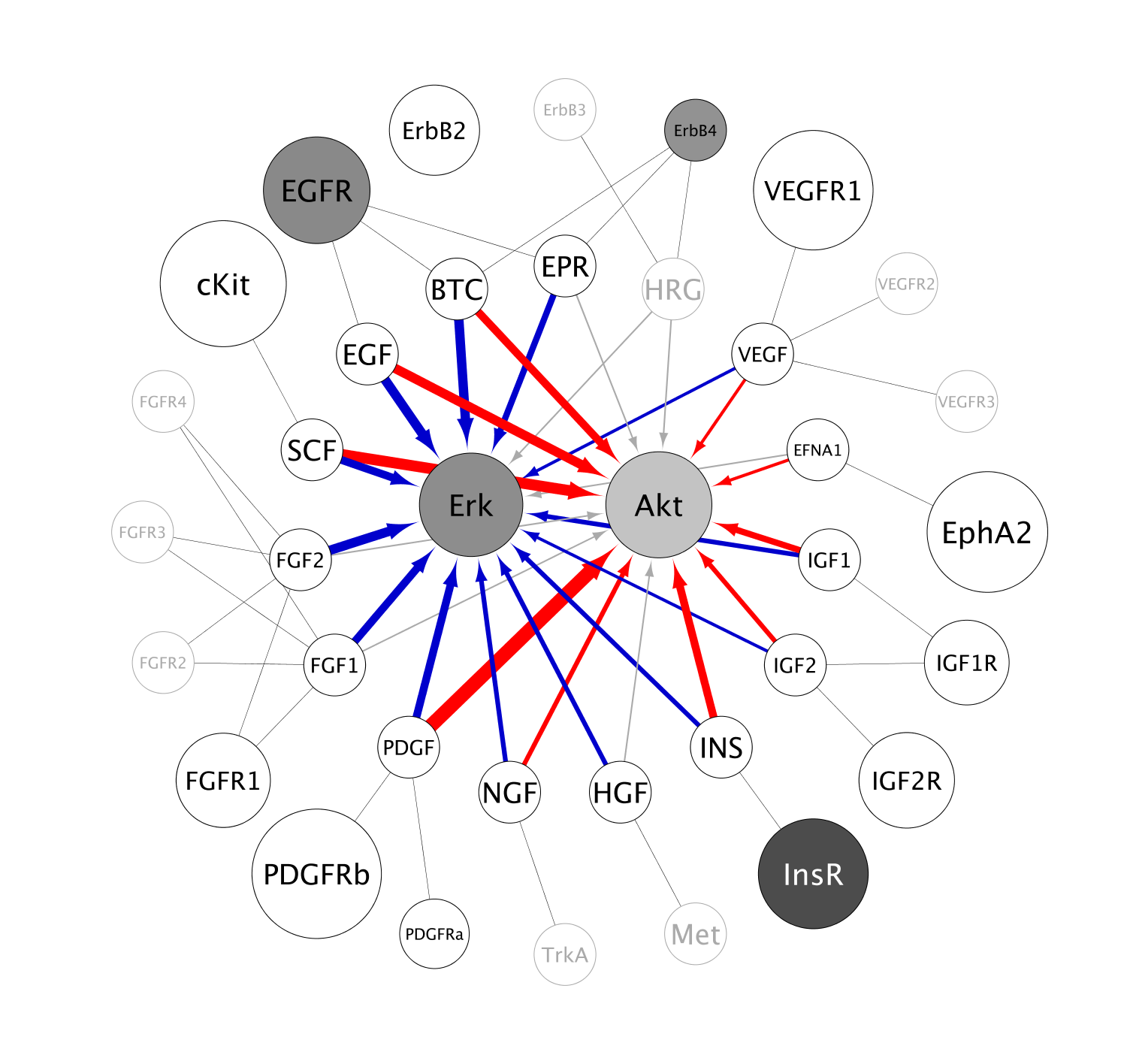

Supplement: Additional file 3 — Network maps of all cell lines used in this study. [file 1741-7007-12-20-S3.zip › MDA-MB-157.png]

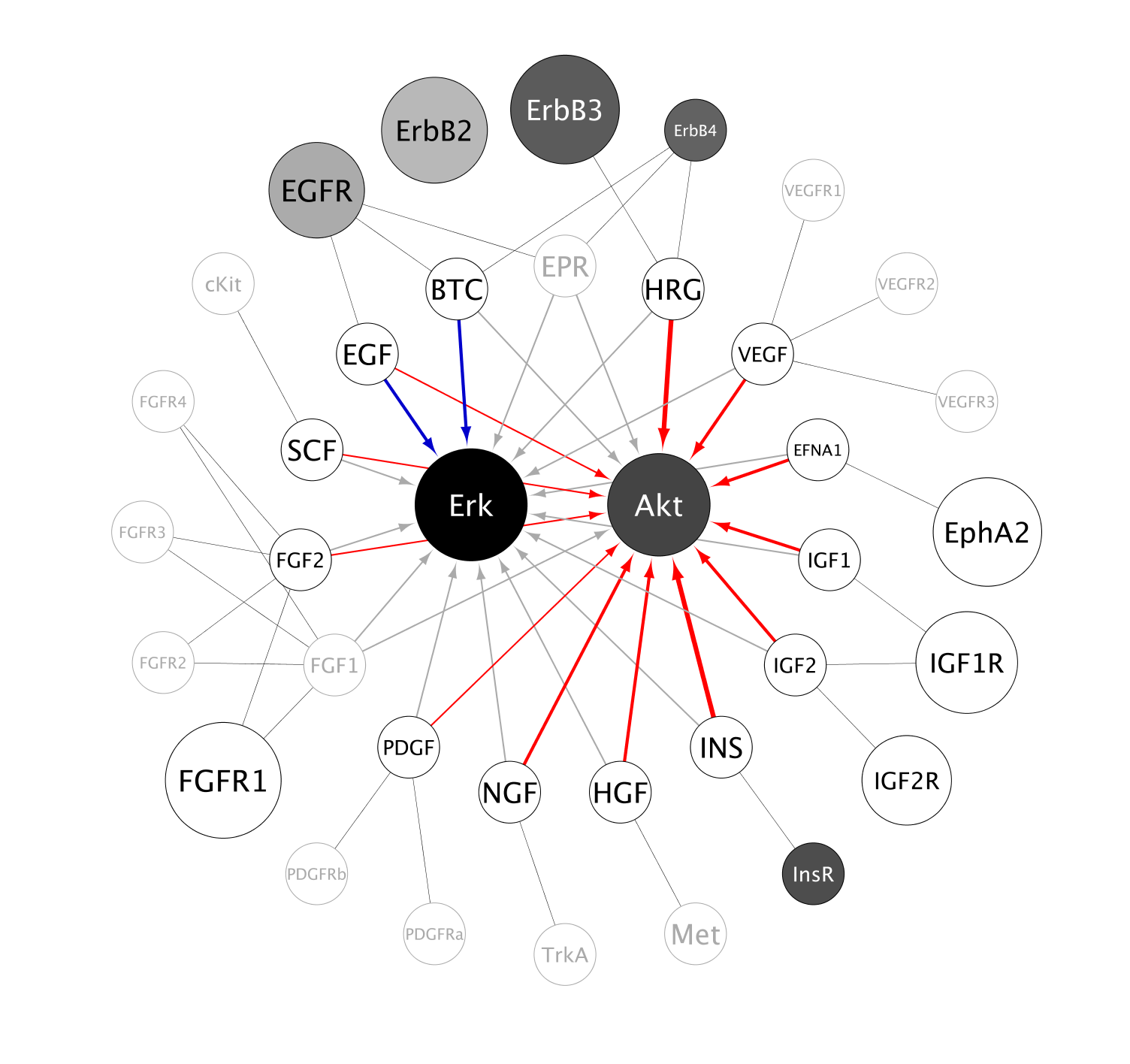

Supplement: Additional file 3 — Network maps of all cell lines used in this study. [file 1741-7007-12-20-S3.zip › MDA-MB-175-VII.png]

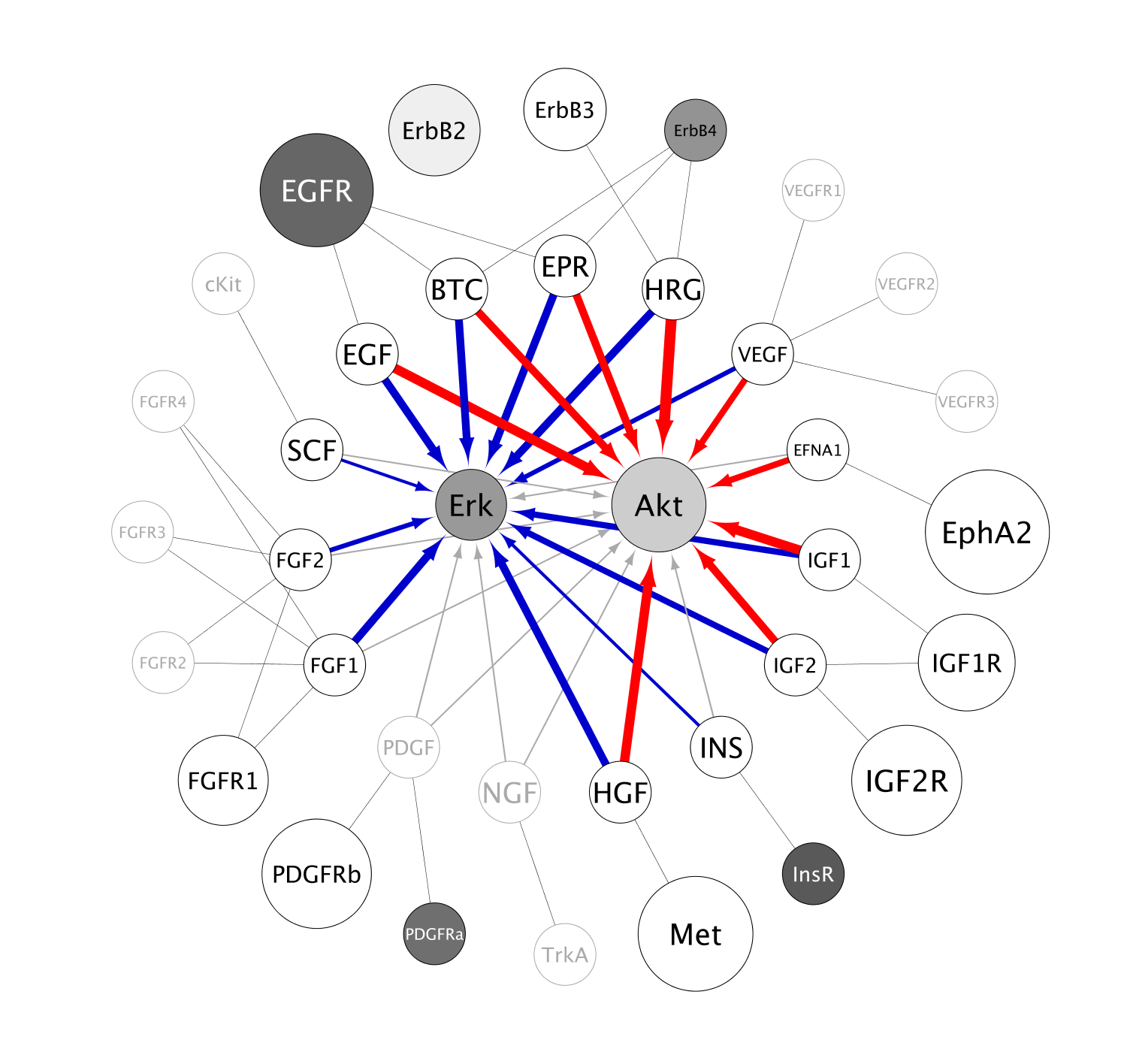

Supplement: Additional file 3 — Network maps of all cell lines used in this study. [file 1741-7007-12-20-S3.zip › MDA-MB-231.png]

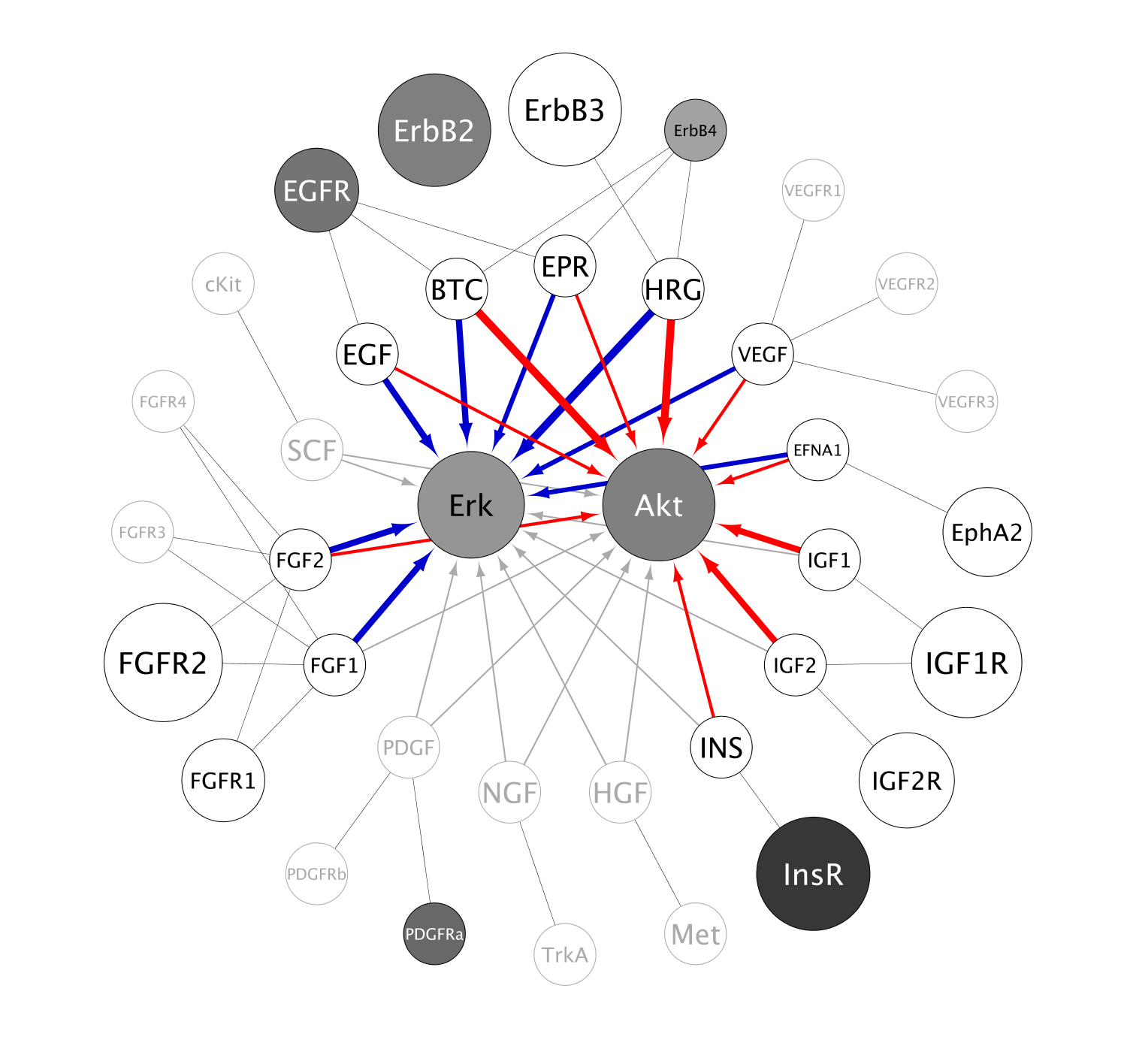

Supplement: Additional file 3 — Network maps of all cell lines used in this study. [file 1741-7007-12-20-S3.zip › MDA-MB-361.png]

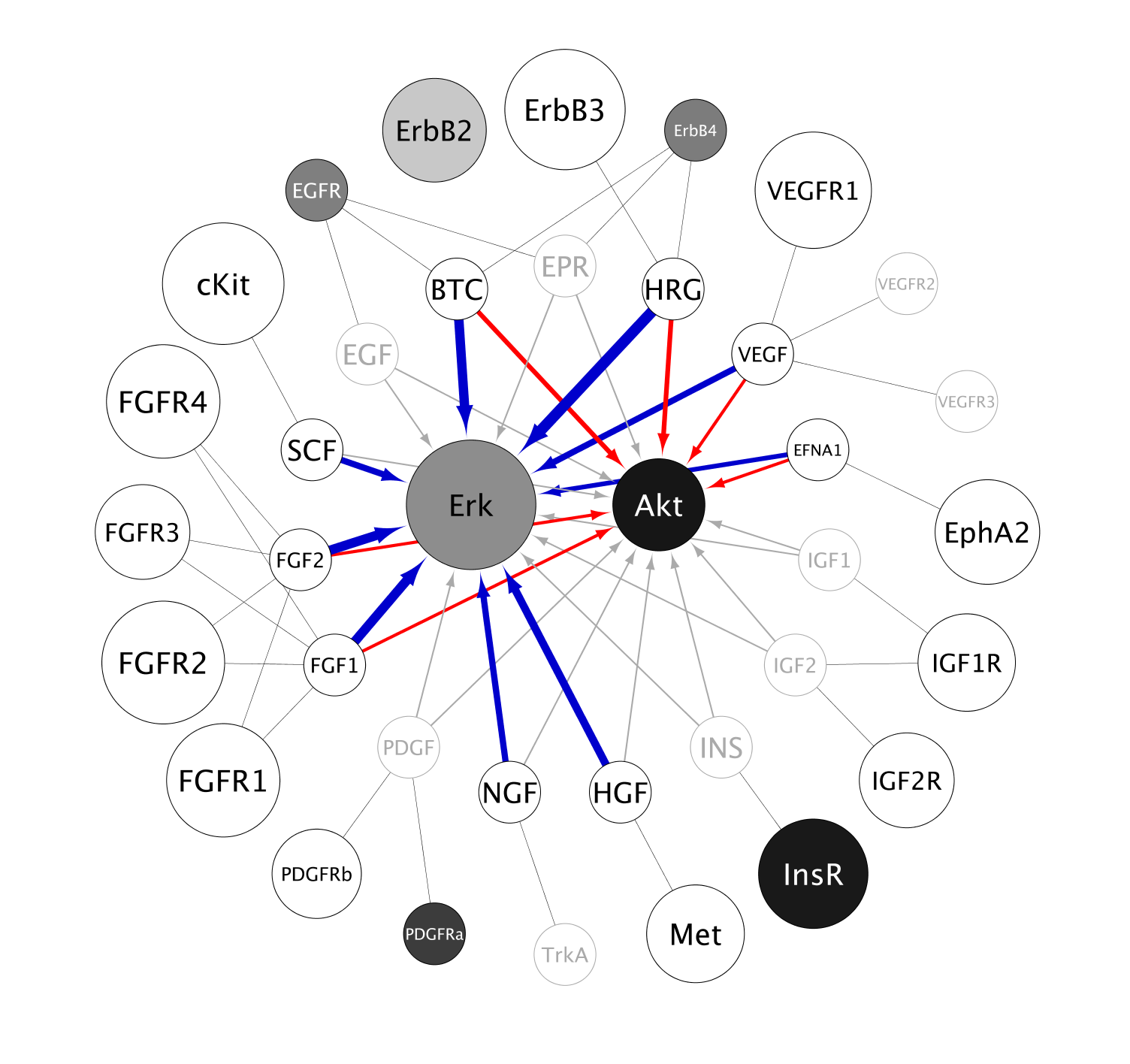

Supplement: Additional file 3 — Network maps of all cell lines used in this study. [file 1741-7007-12-20-S3.zip › MDA-MB-415.png]

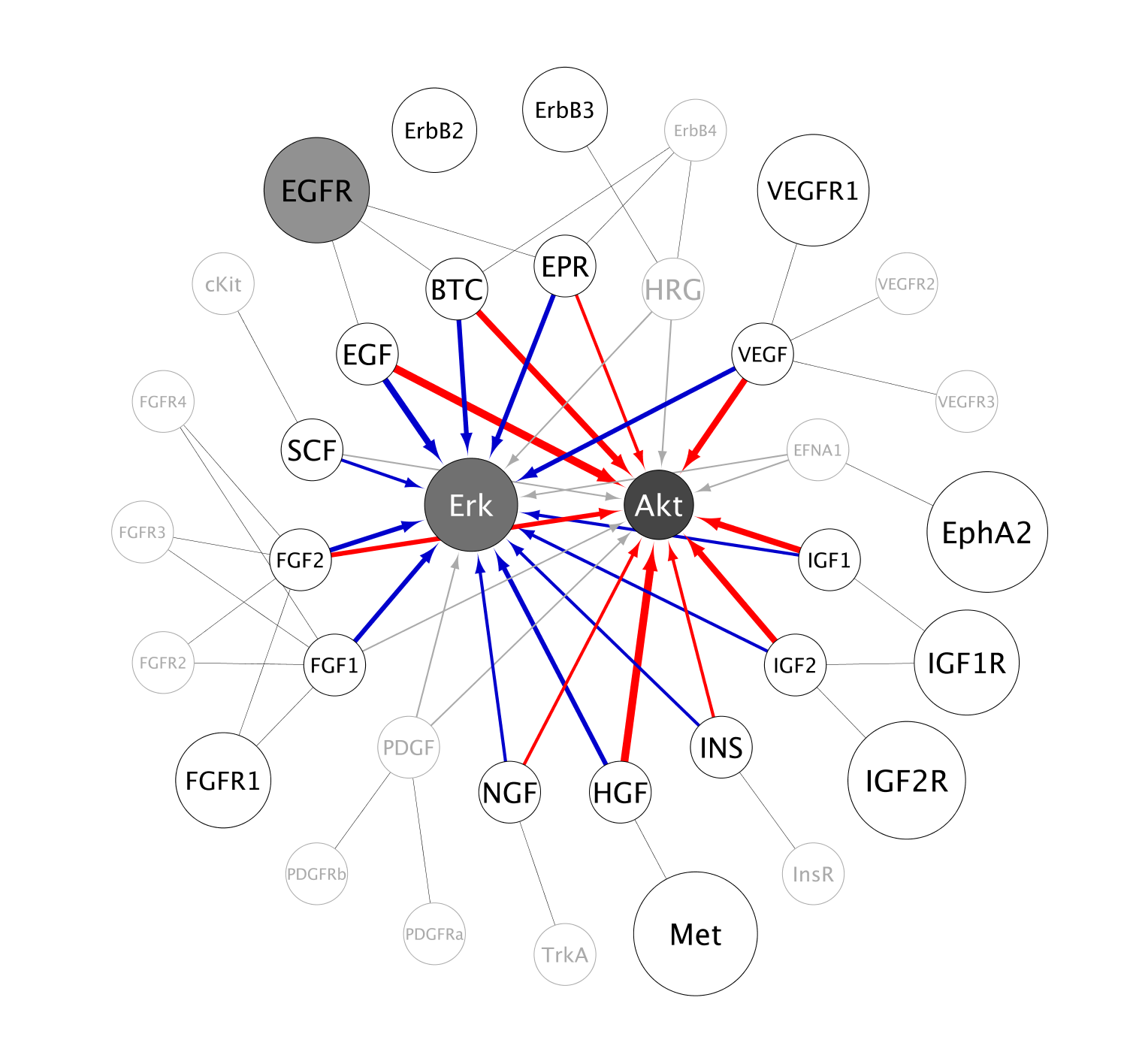

Supplement: Additional file 3 — Network maps of all cell lines used in this study. [file 1741-7007-12-20-S3.zip › MDA-MB-436.png]

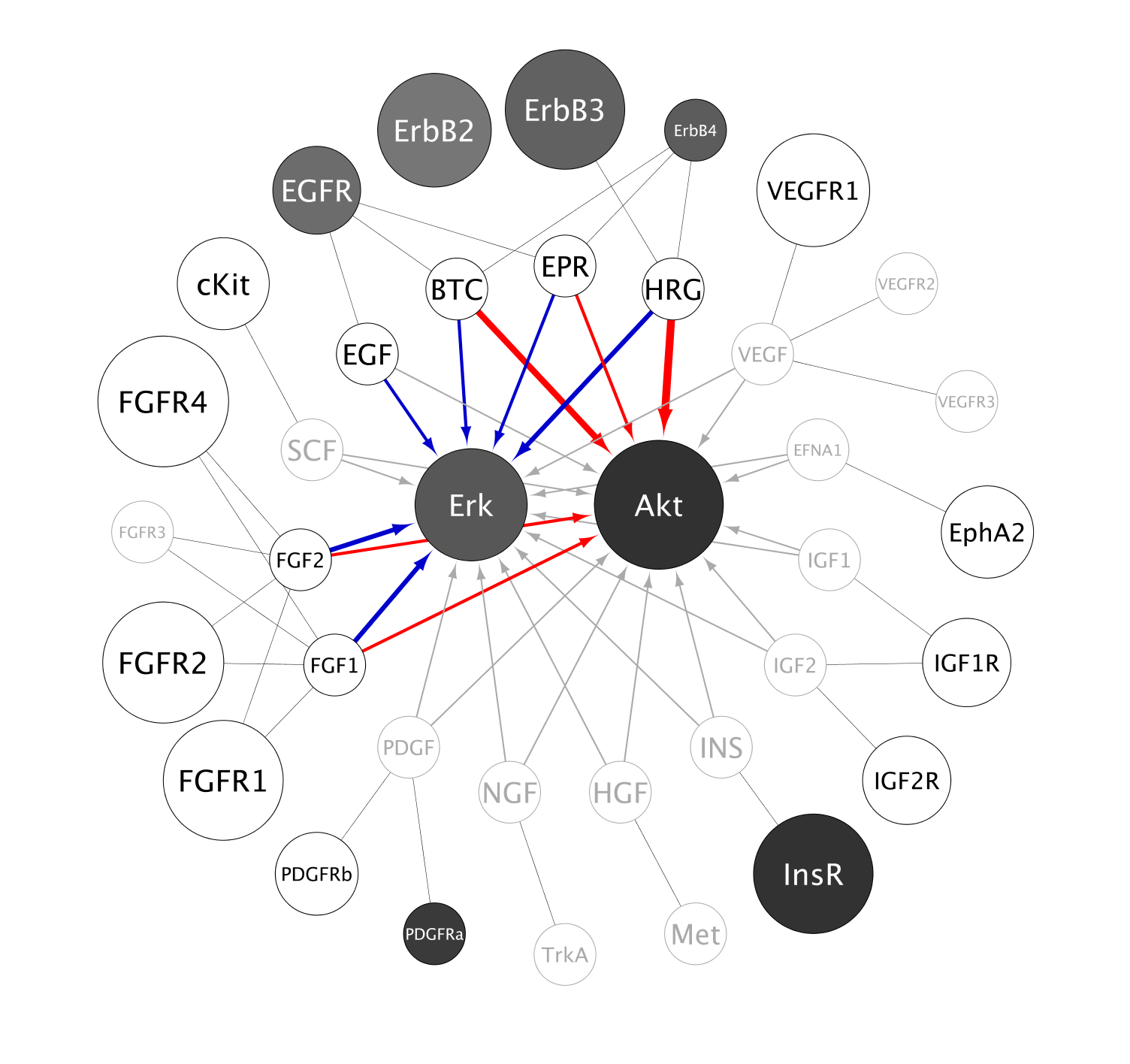

Supplement: Additional file 3 — Network maps of all cell lines used in this study. [file 1741-7007-12-20-S3.zip › MDA-MB-453.png]

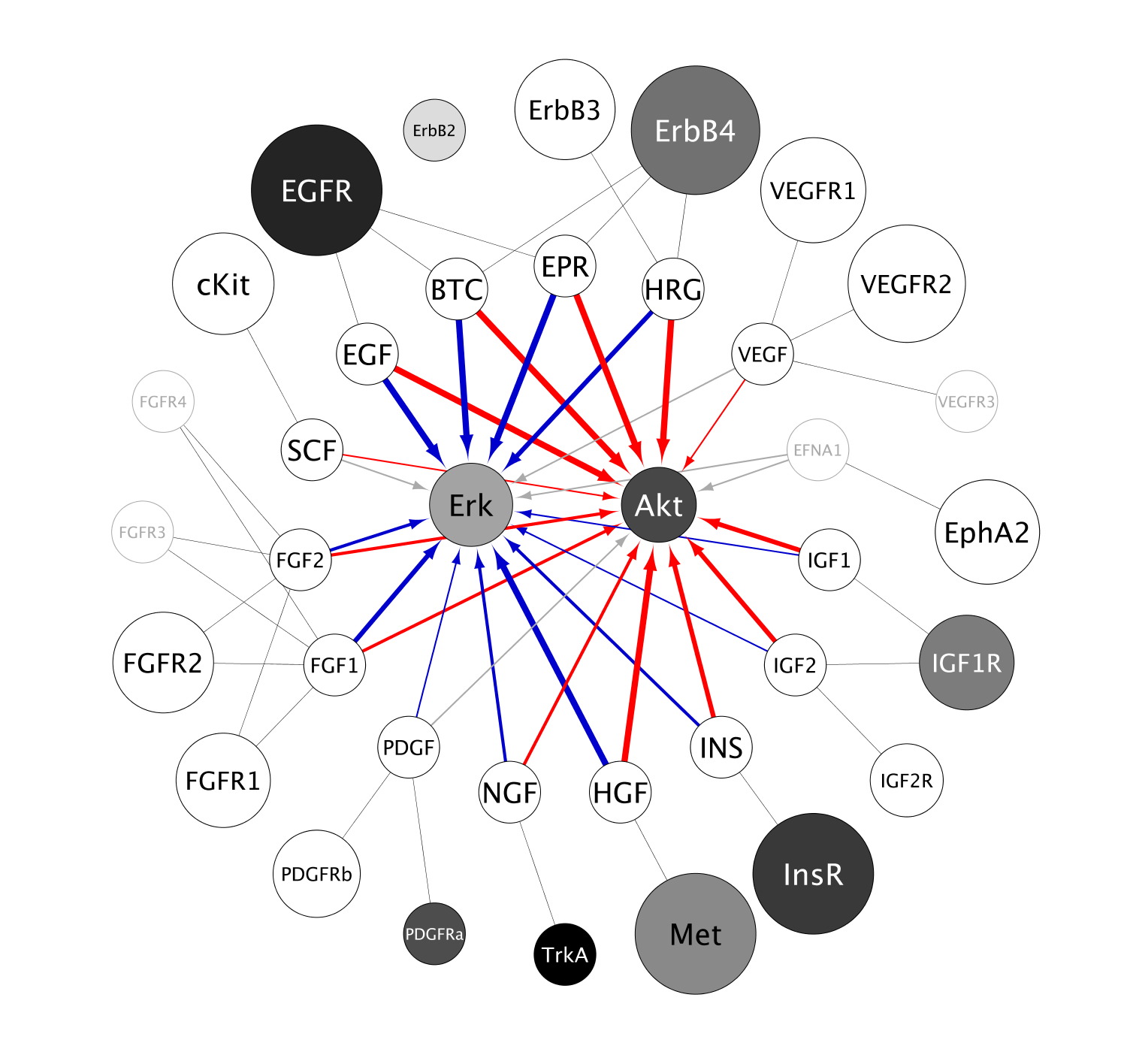

Supplement: Additional file 3 — Network maps of all cell lines used in this study. [file 1741-7007-12-20-S3.zip › MDA-MB-468.png]

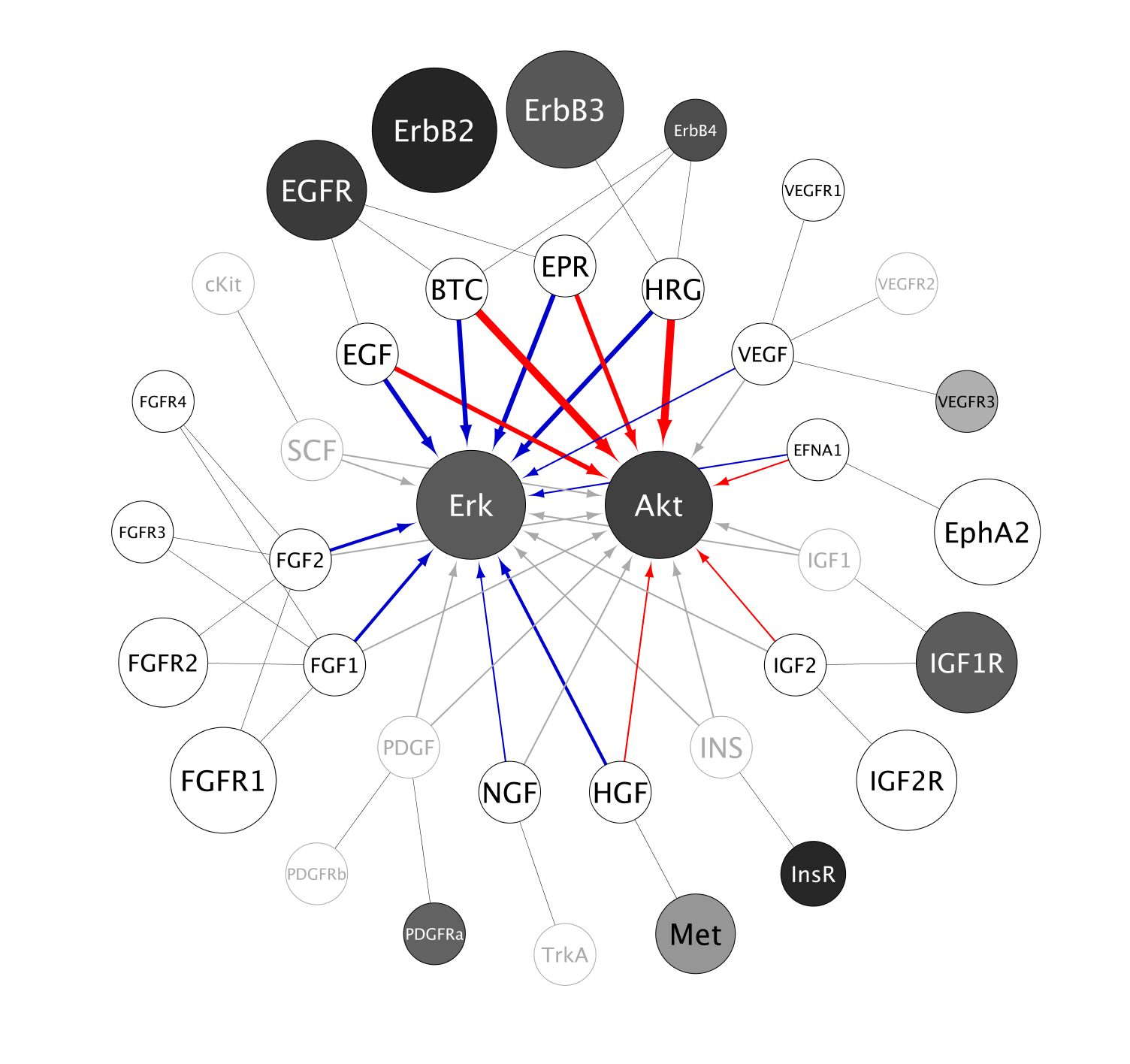

Supplement: Additional file 3 — Network maps of all cell lines used in this study. [file 1741-7007-12-20-S3.zip › NetMap_HER2amp.png]

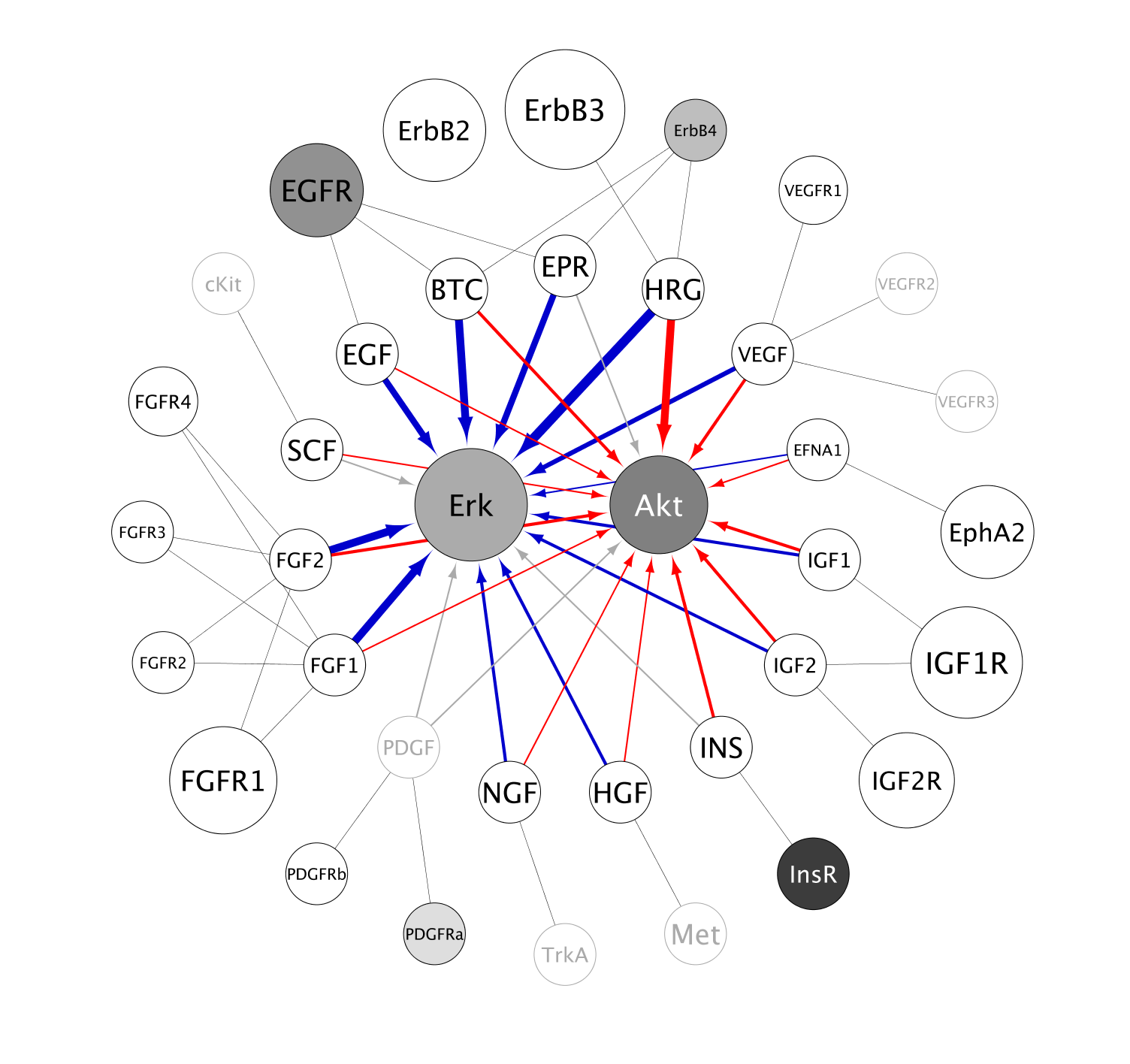

Supplement: Additional file 3 — Network maps of all cell lines used in this study. [file 1741-7007-12-20-S3.zip › NetMap_HR+.png]

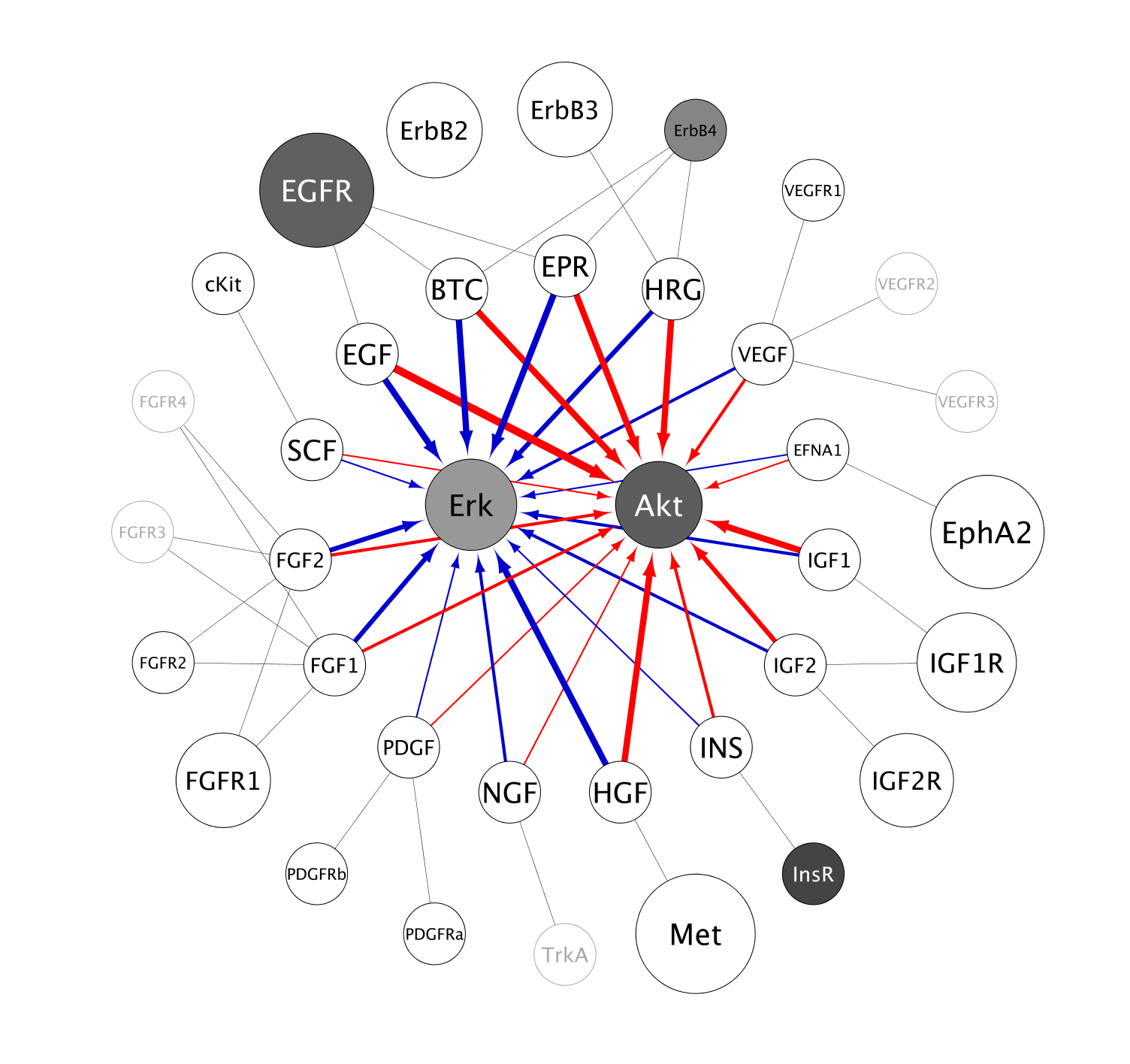

Supplement: Additional file 3 — Network maps of all cell lines used in this study. [file 1741-7007-12-20-S3.zip › NetMap_TNBC.png]

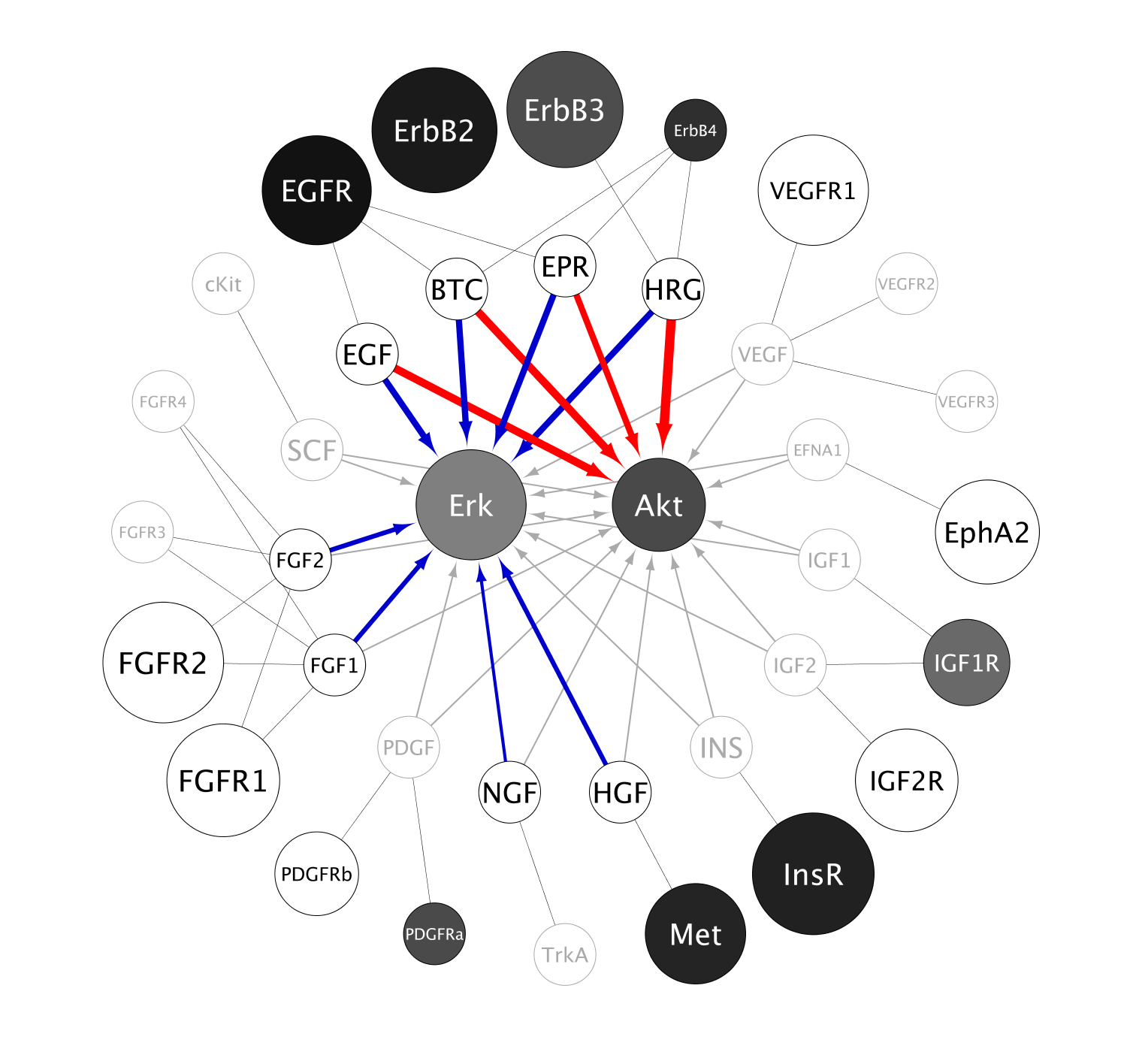

Supplement: Additional file 3 — Network maps of all cell lines used in this study. [file 1741-7007-12-20-S3.zip › SK-BR-3.png]

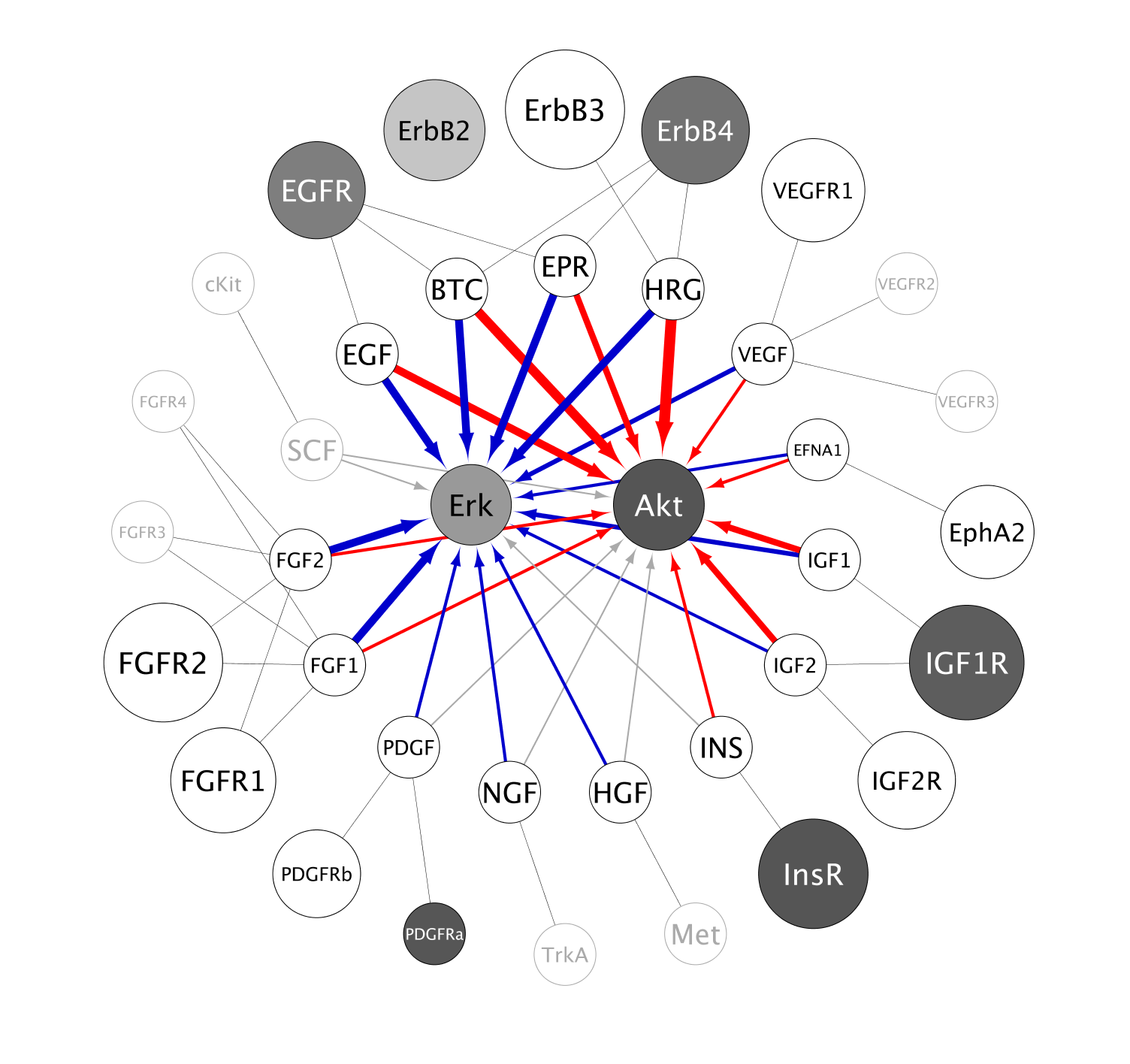

Supplement: Additional file 3 — Network maps of all cell lines used in this study. [file 1741-7007-12-20-S3.zip › T47D.png]

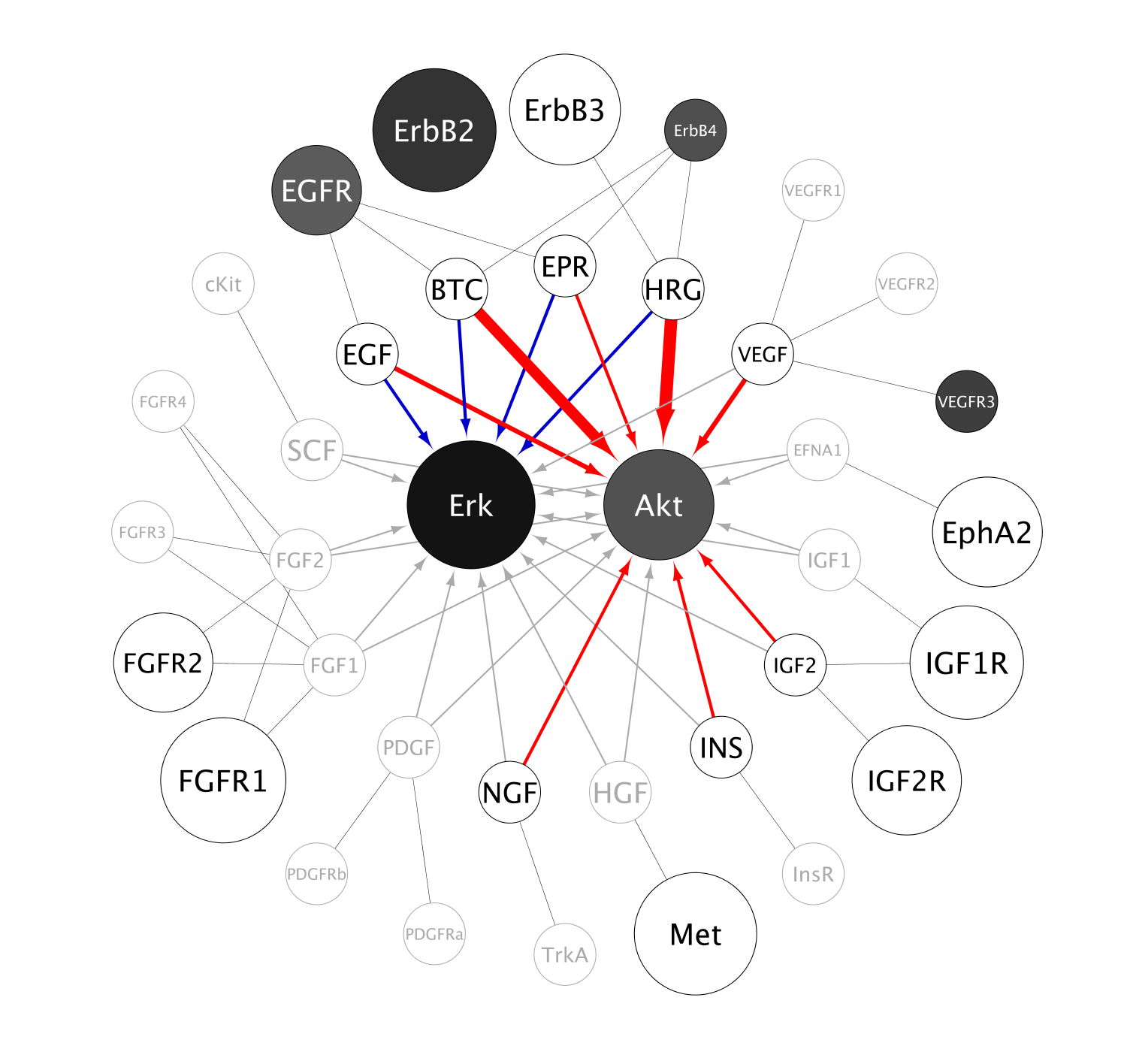

Supplement: Additional file 3 — Network maps of all cell lines used in this study. [file 1741-7007-12-20-S3.zip › UACC-812.png]

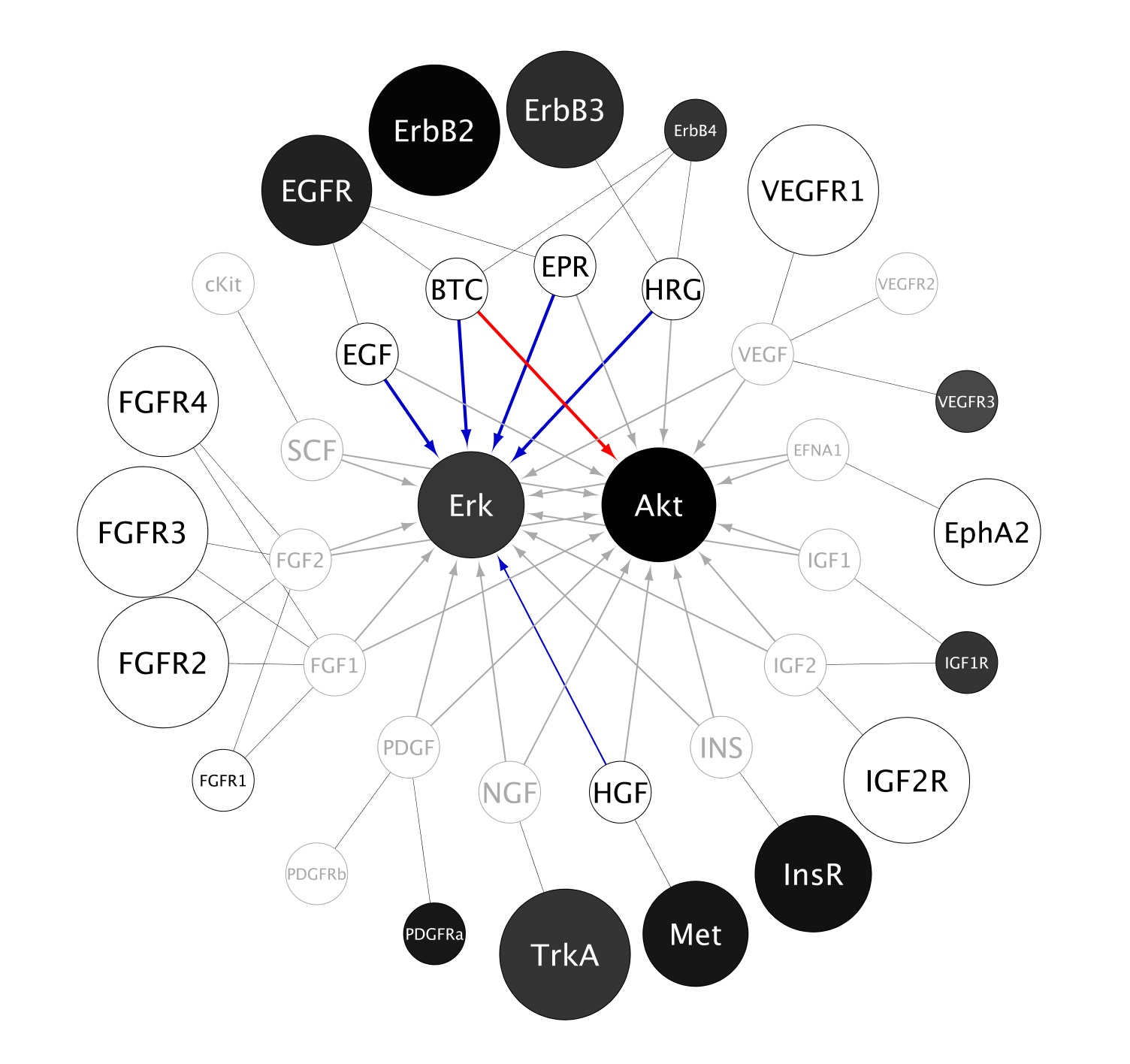

Supplement: Additional file 3 — Network maps of all cell lines used in this study. [file 1741-7007-12-20-S3.zip › UACC-893.png]

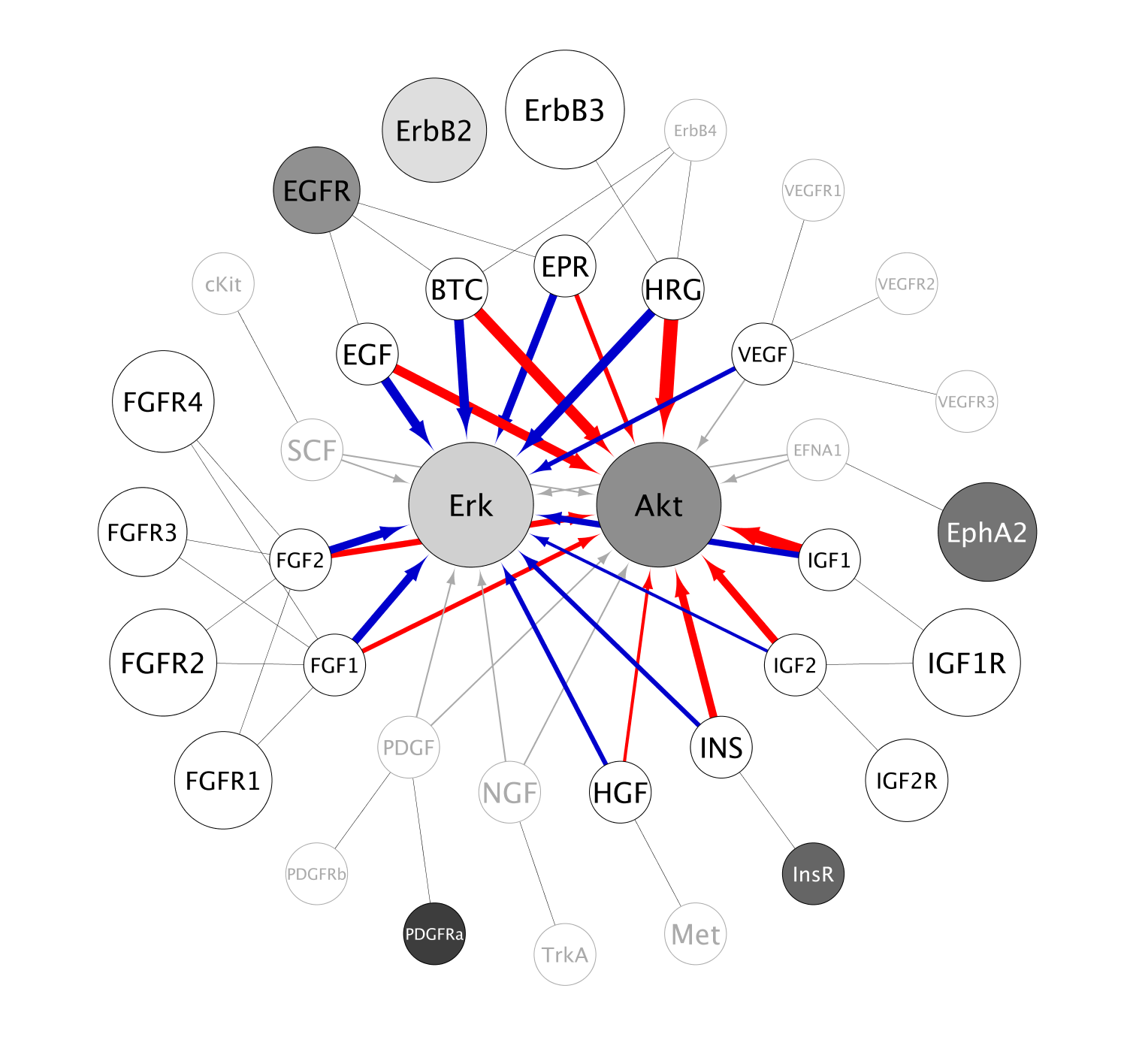

Supplement: Additional file 3 — Network maps of all cell lines used in this study. [file 1741-7007-12-20-S3.zip › ZR-75-1.png]

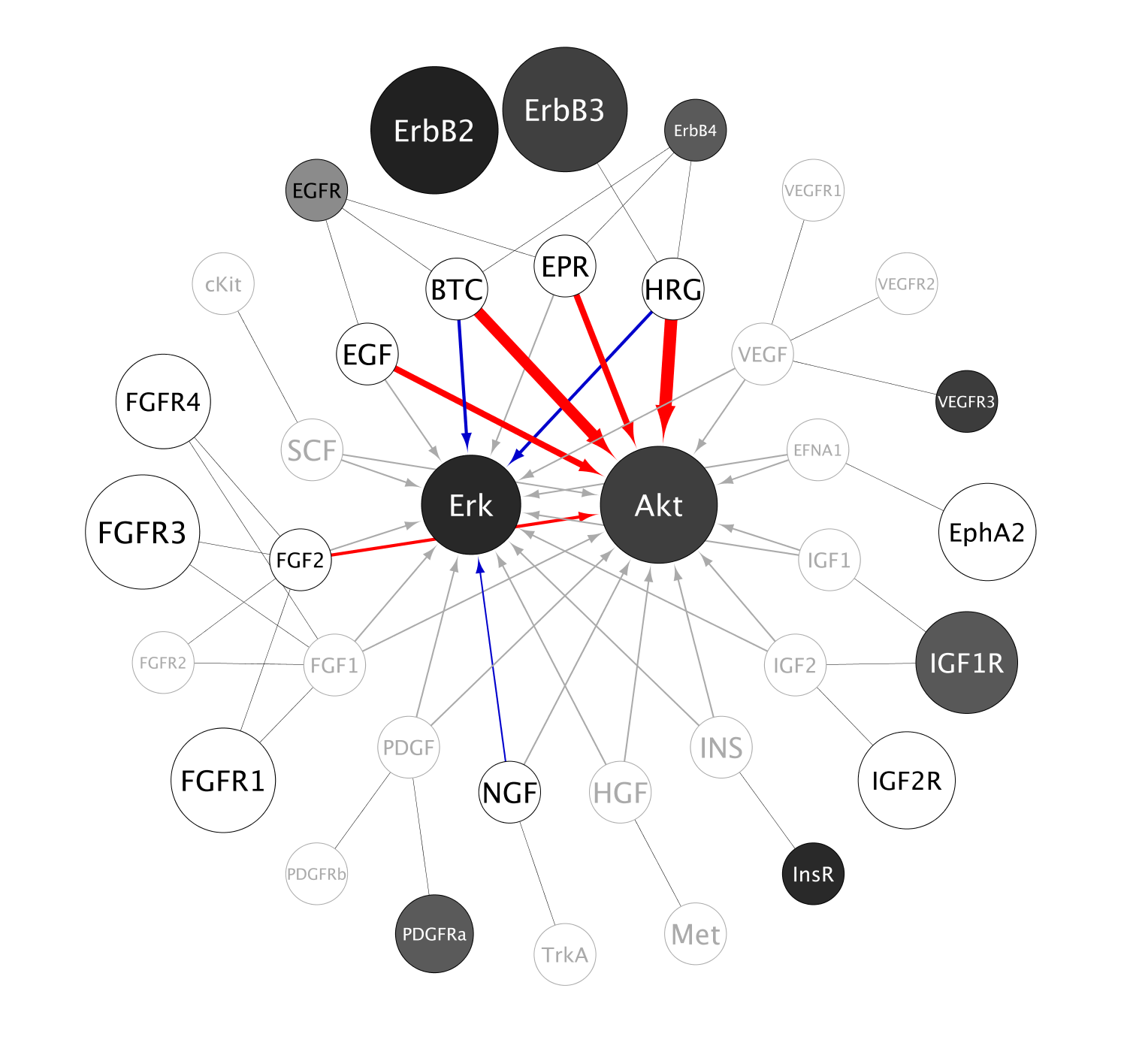

Supplement: Additional file 3 — Network maps of all cell lines used in this study. [file 1741-7007-12-20-S3.zip › ZR-75-30.png]
