# Supplementary material for: Analysis of growth factor signaling in genetically diverse breast cancer lines
Source: BMC Biol. 2014 Mar 21;12:20. doi: 10.1186/1741-7007-12-20 (PMC4234128; doi:10.1186/1741-7007-12-20)
Supplement: Additional file 4: Figure S1 — Correlation between selected basal measurements. [file 1741-7007-12-20-S4.pdf]

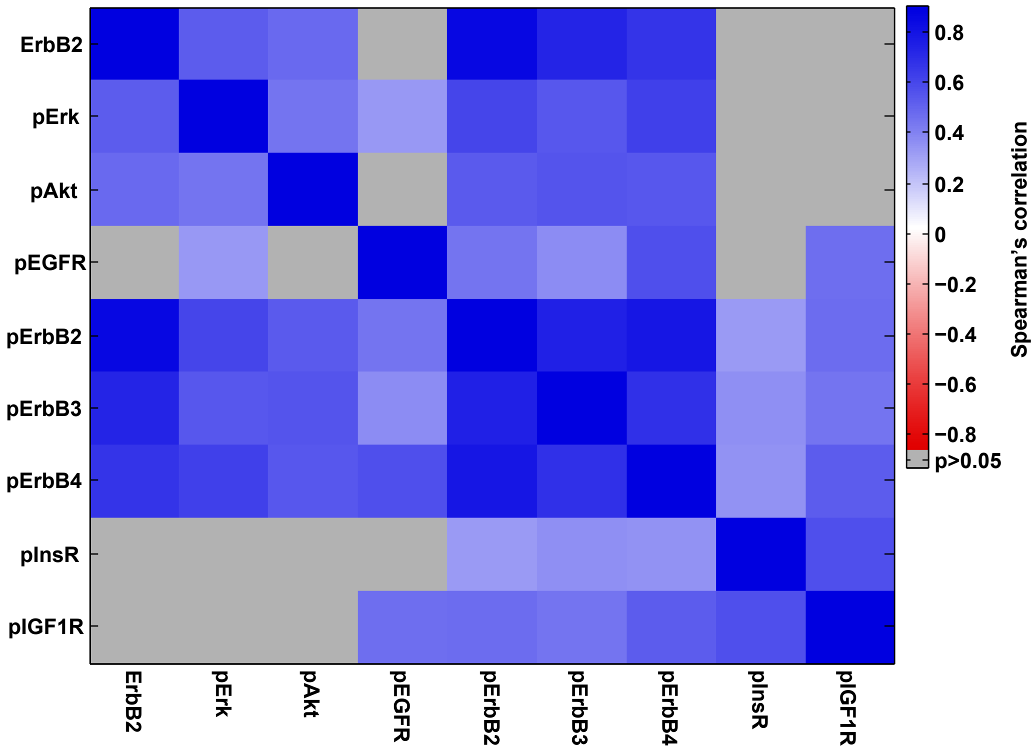

Supplemental Figure S1:

Correlation between selected basal measurements. Correlations between pairs of basal expression or phosphorylation levels of selected RTKs. Blue and red squares denote positive and negative Spearman's correlation respectively, with darker colors denoting a higher correlation coefficient. Gray squares signify non-significant correlations.
