# Supplementary material for: Analysis of growth factor signaling in genetically diverse breast cancer lines
Source: BMC Biol. 2014 Mar 21;12:20. doi: 10.1186/1741-7007-12-20 (PMC4234128; doi:10.1186/1741-7007-12-20)
Supplement: Additional file 6: Figure S2 — Distribution of responses and their fold-change according to the kinetic clusters and dose-dependence and distribution among sensitivity classes of response fold-change. [file 1741-7007-12-20-S6.pdf]

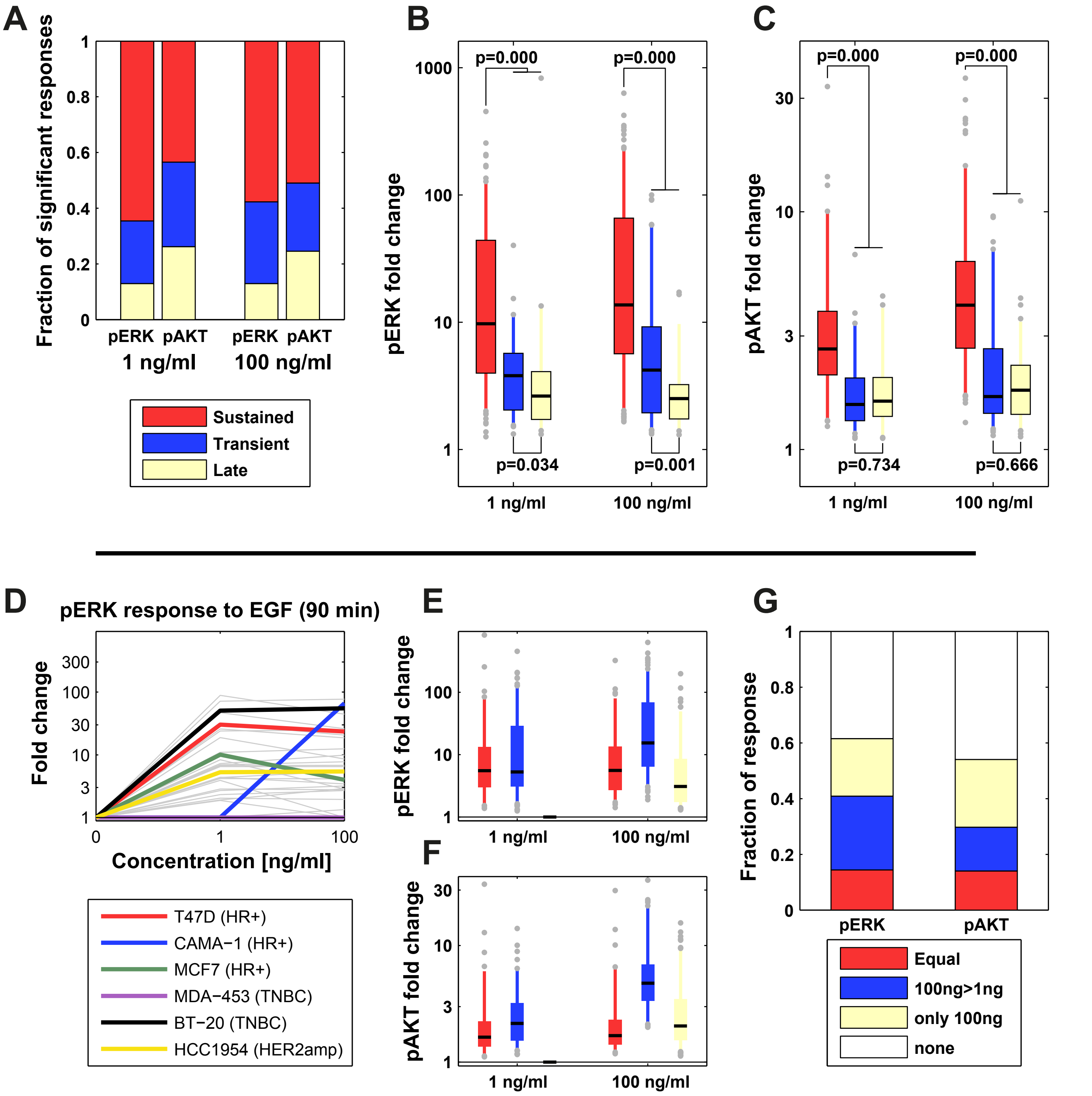

SUPPLEMENTAL FIGURE S2:

Distribution of responses and their fold-change according to the kinetic clusters and dose-dependence. (A) Fraction of the response for pAKT and pERK for the three different kinetic clusters across all responses at 1 ng/ml (left) and 100 ng/ml (right). **(B-C)** Distribution of the fold change of the responses for the different kinetic clusters at 1 ng/ml and 100 ng/ml for (B) pERK response and (C) pAKT. (red) sustained cluster, (blue) transient cluster, and (off-white) late cluster. **(D)** Fold change pERK response across the two measured doses of EGF at 90 min. The thick colored lines represent the six cell lines shown in Figure 1D-F; remaining cell lines are plotted in light gray. **(E-F)** Distribution of fold change for pAKT (E) and pERK (F) across the different classes of response. The black bar is the median, the box span over the interquartile, the line is the 5-95% quantile and values outside this range are plotted as individual dots. **(G)** Fraction of responses by class for pERK and pAKT. (red) "equal", low dose response is at least 75% of the high dose one; (blue) "100 ng > 1 ng", response at low dose is less than 75% of high dose but still significant; (off-white) "only 100 ng", responses are significant at 100 ng/ml but not at 1 ng/ml; (white) "none", neither high nor low dose responses are significant.
