# Supplementary material for: Analysis of growth factor signaling in genetically diverse breast cancer lines
Source: BMC Biol. 2014 Mar 21;12:20. doi: 10.1186/1741-7007-12-20 (PMC4234128; doi:10.1186/1741-7007-12-20)
Supplement: Additional file 7: Figure S3 — Enrichment for basal RTK levels among cell lines with significant responses. [file 1741-7007-12-20-S7.pdf]

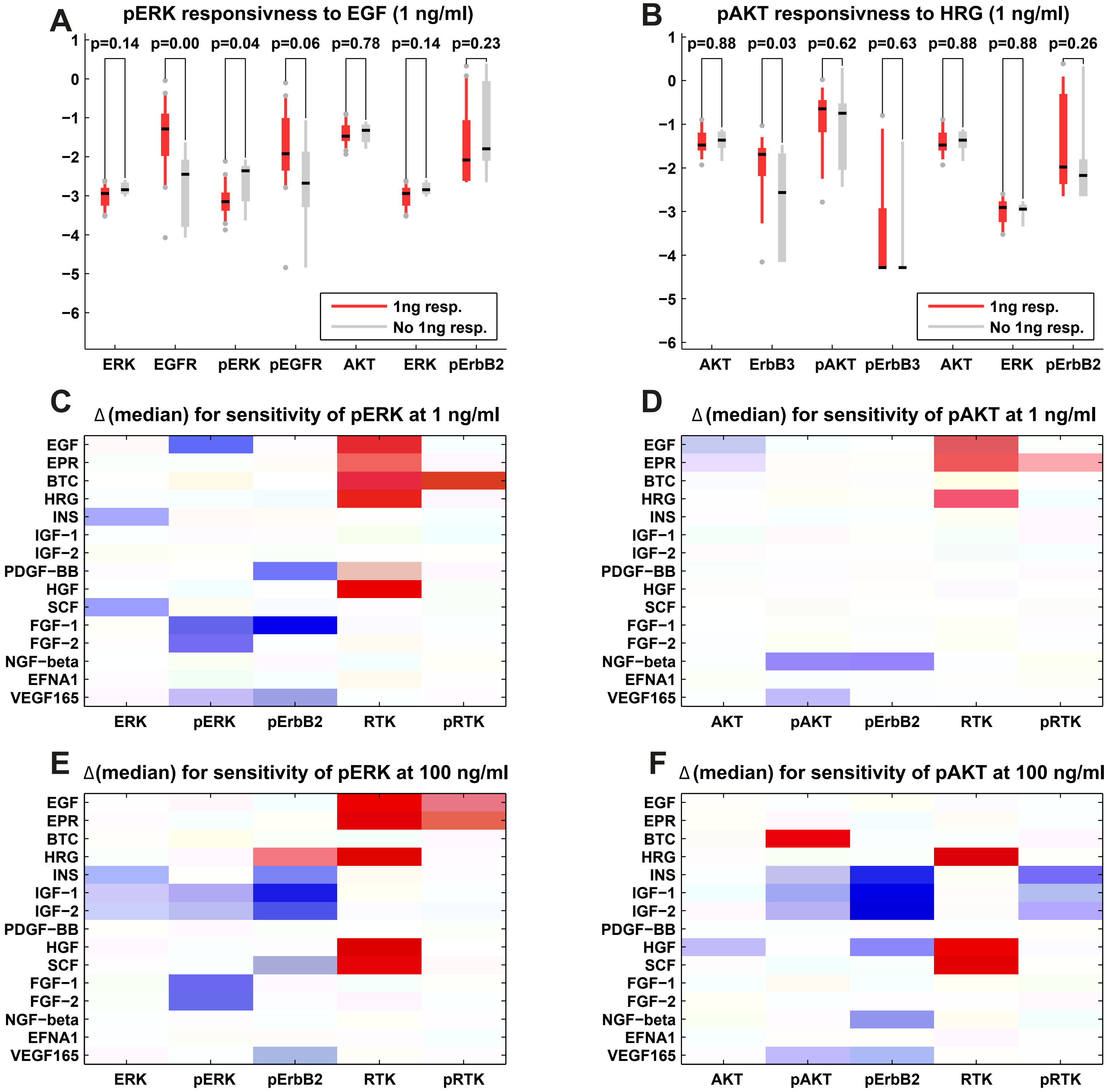

SUPPLEMENTAL FIGURE S3:

Enrichment for basal RTK levels among cell lines likely to respond. (A) Distributions of relevant basal RTK levels (expression or phosphorylation) for cell lines treated with 1ng/ml EGF that (red) show a significant pERK response and (gray) the ones that do not. Significance of the difference between the two distributions is assessed by Wilcoxon rank-sum test. **(B)** Distribution of relevant basal RTK levels (expression or phosphorylation) for cell lines that show a significant pAKT response following 1 ng/ml HRG stimulation or not – plotted as in (A). **(C-F)** Enrichment in basal and phosphorylated target levels, pErbB2 levels, and basal and phosphorylated cognate receptor levels for the cell lines that respond to ligands on pERK (C,E) or pAKT (D,F) for 1 ng/ml (C-D) and 100 ng/ml (E-F). (red) positive and (blue) negative enrichment in the responding lines, (white) non-significant correlation ($p > 0.05$) assessed with a Wilcoxon rank-sum test. Color intensity corresponds to the difference in the median between the responsive and the non-responsive lines.
