# Supplementary material for: Analysis of growth factor signaling in genetically diverse breast cancer lines
Source: BMC Biol. 2014 Mar 21;12:20. doi: 10.1186/1741-7007-12-20 (PMC4234128; doi:10.1186/1741-7007-12-20)
Supplement: Additional file 8: Figure S4 — Relationship between basal pErbB2, PI3K/PTEN mutational status and the basal levels of pAKT and pERK. [file 1741-7007-12-20-S8.pdf]

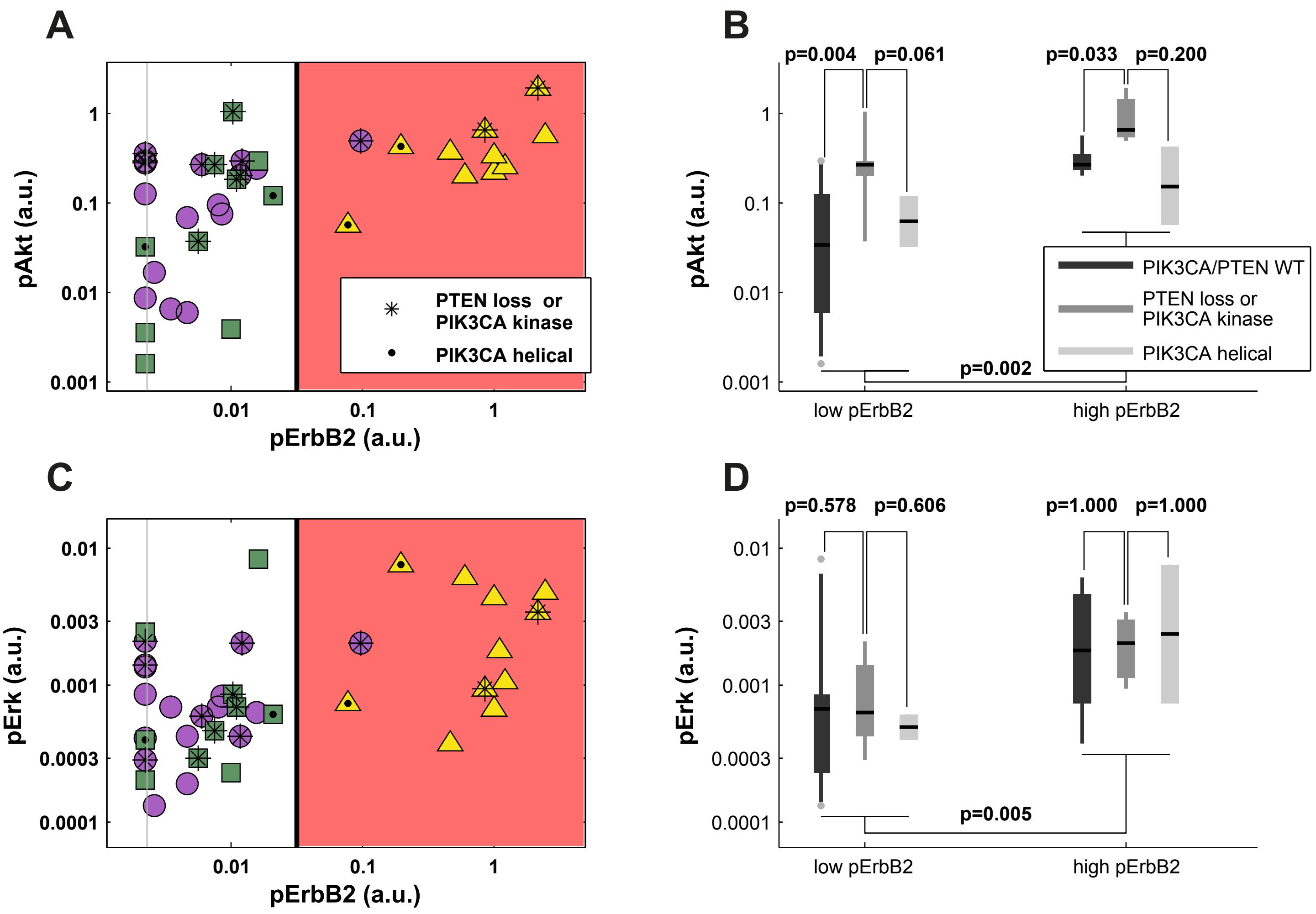

Supplemental Figure S4:

Relationship between basal pErbB2, PI3K/PTEN mutational status and the basal levels of pAKT and pERK. **(A)** Cell lines with high pErbB2 (red zone) have high pAKT levels ($p = 0.002$, Wilcoxon rank-sum test) independently of their PIK3CA mutation status (star for PTEN loss of function or PIK3CA kinase domain mutation; dot for PIK3CA helical domain mutation). **(B)** Cell lines with a PTEN loss of function or a PIK3CA kinase domain mutation have higher pAKT levels than the wild type ones in both groups of low and high pErbB2 level ($p = 0.004$ and $p = 0.033$ respectively, Wilcoxon rank-sum test). **(C)** Cell lines with high pErbB2 (red zone) have high pERK ($p = 0.005$, Wilcoxon rank-sum test). **(D)** PTEN or PIK3CA mutation status has no impact of the level of pERK ($p > 0.05$, Wilcoxon rank-sum test).
